# Cytoskeletal Imbalance and Axonal Vulnerability in Sporadic PSP-RS: Early Changes in a Human iPSC-Derived Neuronal Model with Altered mTOR Signaling

**DOI:** 10.3390/cells15090754

**Published:** 2026-04-23

**Authors:** Raffaele Covello, Giorgia Lucia Benedetto, Stefania Scalise, Caterina Gabriele, Desirèe Valente, Clara Zannino, Barbara Puccio, Andrea Quattrone, Pietro Hiram Guzzi, Marco Gaspari, Aldo Quattrone, Giovanni Cuda, Elvira Immacolata Parrotta

**Affiliations:** 1 Department of Experimental and Clinical Medicine, University Magna Graecia of Catanzaro, 88100 Catanzaro, Italy; raffaele.covello@studenti.unicz.it (R.C.);; 2 Department of Medical and Surgical Sciences, University Magna Graecia of Catanzaro, 88100 Catanzaro, Italy; giorgialucia.benedetto@studenti.unicz.it (G.L.B.); parrotta@unicz.it (E.I.P.); 3 Department of Experimental Medical Science, Lund University, 22100 Lund, Sweden; 4 Neuroscience Research Center, University Magna Graecia of Catanzaro, 88100 Catanzaro, Italy

**Keywords:** progressive supranuclear palsy, axonal degeneration, cytoskeletal dysregulation, Tau pathology, dopaminergic neurons, mTOR signaling

## Abstract

**Highlights:**

**What are the main findings?**

- iPSC-derived PSP-RS neurons recapitulate early disease features, including Tau hyperphosphorylation, neurofilament accumulation, and progressive loss of dopaminergic neurons and synaptic identity.
- Multi-omics profiling reveals a coordinated shift from synaptic function to cytoskeletal remodeling and axonal vulnerability.
- mTOR signaling is upregulated in PSP-RS neurons, and its inhibition reduces Tau phosphorylation and neurofilament accumulation.

**What are the implications of the main findings?**

- Cytoskeletal dysfunction in PSP-RS reflects an early axonopathic process driven by converging structural and signaling alterations.
- mTOR acts as a modulatory pathway that sustains cytoskeletal imbalance rather than initiating pathology.
- This iPSC-based model provides a human platform to investigate early disease mechanisms and to explore therapeutic strategies targeting axonal homeostasis.

**Abstract:**

Progressive supranuclear palsy-Richardson’s syndrome (PSP-RS) is a primary 4R tauopathy in which early axonal dysfunction may precede overt neurodegeneration; however, the mechanisms linking Tau dysregulation to cytoskeletal vulnerability remain poorly defined. Here, we generated induced pluripotent stem cell (iPSC)-derived midbrain dopaminergic neurons from individuals with sporadic PSP-RS and matched healthy controls and performed integrated transcriptomic and proteomic analyses. PSP-RS neurons exhibited coordinated suppression of dopaminergic and synaptic programs alongside activation of cytoskeletal remodeling and stress-related pathways. These changes were accompanied by increased Tau phosphorylation, neurofilament accumulation, and structural alterations of the axonal compartment, consistent with an early axonopathic phenotype. Notably, mechanistic target of rapamycin (mTOR) signaling significantly increased. Pharmacological inhibition of mTOR reduced Tau phosphorylation and neurofilament levels, indicating that mTOR activity contributes to the maintenance of cytoskeletal imbalance. In conclusion, our findings support a model in which early cytoskeletal dysfunction in PSP-RS arises from the convergence of Tau dysregulation, impaired structural homeostasis, and altered signaling pathways. Rather than acting as a primary driver, mTOR appears to function as a pathogenic amplifier that sustains axonal stress. This study provides a human cellular framework to investigate early axonopathic mechanisms in sporadic PSP-RS.

## 1. Introduction

Progressive supranuclear palsy (PSP), particularly the Richardson’s syndrome subtype (PSP-RS), is a primary 4-repeat (4R) tauopathy clinically characterized by early postural instability, vertical gaze palsy, and progressive motor and cognitive decline [1]. Among PSP phenotypes, PSP-RS represents the most common and clinically aggressive form, whereas PSP-parkinsonism (PSP-P) follows a more indolent course, suggesting mechanistic heterogeneity [2]. Neuropathologically, PSP-RS is characterized by widespread degeneration of subcortical and cortical structures, reflecting profound disruption of neuronal connectivity and axonal integrity [3]. A central pathological hallmark of PSP-RS is the accumulation of hyperphosphorylated Tau in neurons and glial cells [4,5]. Under physiological conditions, Tau stabilizes microtubules and supports axonal transport [6,7,8]; however, its pathological modification disrupts cytoskeletal integrity and intracellular trafficking [9,10,11]. Increasing evidence indicates that axonal dysfunction is not merely a late consequence of neurodegeneration but an early and potentially initiating event in tauopathies [12,13,14]. Despite this, the cellular mechanisms linking Tau dysregulation to early cytoskeletal vulnerability in sporadic PSP-RS remain incompletely defined. In particular, it is unclear whether additional structural and signaling pathways cooperate with Tau pathology to drive early axonal dysfunction [15]. Neurofilaments (NFs), major components of the axonal cytoskeleton [16,17], are widely used as biomarkers of axonal injury in PSP [18,19,20,21]. However, whether their intracellular accumulation directly contributes to cytoskeletal imbalance, rather than reflecting downstream damage, remains unresolved. Consistent with observations in other neurodegenerative disorders, emerging evidence suggests that intracellular neurofilament dysregulation may actively contribute to axonal pathology [22,23,24,25]. Recent literature indicates that these mechanisms differ between PSP clinical phenotypes. PSP-RS exhibits more pronounced alterations in iron metabolism and inflammatory signaling, while the Parkinsonian variant (PSP-P) shows distinct neurotrophic responses [26,27,28]. This phenotypic heterogeneity suggests that dysregulation of the GDNF/hepcidin axis may differentially affect disease progression depending on PSP subtype, emphasizing the need for subtype-specific interpretation of biomarkers and integration of clinical phenotypes with molecular signatures in future PSP research. Moreover, pathways regulating proteostasis and structural remodeling, such as the mechanistic target of rapamycin (mTOR) signaling, have been implicated in neurodegeneration [29], although their specific role in PSP-RS remains poorly defined. Midbrain dopaminergic (mDA) neurons are particularly vulnerable in PSP-RS due to their complex axonal architecture and high metabolic demand [30,31]. However, mechanistic investigation of early disease processes has been limited by the lack of human models that faithfully recapitulate pre-degenerative stages. Here, we use iPSC-derived mDA neurons from individuals with sporadic PSP-RS to model early disease-associated alterations. We identify a coordinated phenotype characterized by Tau hyperphosphorylation, neurofilament accumulation, and axonal structural remodeling. Furthermore, we show that mTOR signaling contributes to maintaining this cytoskeletal imbalance. These findings support a model in which early axonopathy in PSP-RS emerges from the convergence of structural and signaling alterations rather than from Tau pathology alone.

## 2. Materials and Methods

### 2.1. Generation and Differentiation of iPSC-Derived Midbrain Dopaminergic Neurons

Midbrain dopaminergic neurons (mDAs) were derived from induced pluripotent stem cell (iPSC) lines obtained from three healthy control (HC) donors (hiPSC-1, HC_002, and HC_003) and three individuals diagnosed with sporadic PSP-RS (PSP_002, PSP_003, and PSP_004). All cell lines had been previously established and characterized [32,33]. The clinical characteristics of PSP-RS patients were previously described [33]. Differentiation into mDAs was performed using the protocol of Kriks et al. (2011) [34], with minor modifications. Cells were maintained on hESC-qualified Matrigel (Corning, Corning, NY, USA) mTeSR™ Plus medium (Stem Cell Technologies, Vancouver, BC, Canada) under standard culture conditions (37 °C and 5% CO_2_). Neural differentiation was initiated upon reaching confluence by replacing the maintenance medium with KnockOut Serum Replacement Medium (SRM), composed of KnockOut DMEM supplemented with 15% KnockOut Serum Replacement, GlutaMAX™, MEM non-essential amino acids (NEAA), 0.2% penicillin–streptomycin, and 55 µM 2-mercaptoethanol (Thermo Fisher Scientific, Waltham, MA, USA). During the initial patterning phase (DIV 0–4), cultures were treated with 100 nM LDN193189 and 10 µM SB431542 to induce dual SMAD inhibition, in combination with 100 ng/mL SHH (C24II), 100 ng/mL FGF8 (Miltenyi Biotec, Bergisch Gladbach, Germany), and 2 µM purmorphamine (Sigma-Aldrich, St. Louis, MO, USA) to promote ventral midbrain identity. From DIV 3 to DIV 11, cells were treated with 3 µM CHIR99021 (Miltenyi Biotec) to enhance WNT signaling and reinforce midbrain specification. Starting at DIV 5, SRM was gradually replaced with N2 medium (DMEM/F12 containing HEPES, GlutaMAX™, N2 supplement, and 0.2% penicillin–streptomycin), while maintaining patterning factors. At DIV 11, midbrain floor plate progenitors (mFPPs) were transferred to Neurobasal/B27 (NB/B27) medium (Neurobasal supplemented with GlutaMAX™, penicillin–streptomycin, and B27; Thermo Fisher Scientific). Neuronal maturation was supported by supplementation with 20 ng/mL brain-derived neurotrophic factor (BDNF; Miltenyi Biotec), 20 ng/mL glial cell line-derived neurotrophic factor (GDNF; R&D Systems, Minneapolis, MN, USA), 1 ng/mL TGF-β3 (PeproTech, Cranbury, NJ, USA), 200 µM ascorbic acid, 0.5 mM dibutyryl-cAMP, and 10 µM DAPT (Tocris, Bristol, United Kingdom). At DIV 20, progenitor cells were dissociated and either maintained as individual lines or pooled. For pooled conditions, equal numbers of progenitors from each donor were mixed to ensure balanced representation. Pooling was performed at the committed progenitor stage to minimize donor-specific variability and prevent unbalanced representation, as previously suggested in related human stem cell–based models [35]. Briefly, mFPPs were dissociated using StemPro™ Accutase (Thermo Fisher Scientific) and replated at a density of 75,000 cells/cm^2^ on coverslips or multiwell plates coated with poly-L-ornithine (15 µg/mL) and laminin (20 µg/mL) (Sigma-Aldrich) in NB/B27 medium. The cultures were maintained for up to 60 days in vitro, with medium changes every 48 h. Cells were collected at defined differentiation stages for downstream analyses. A detailed list of the reagents is provided in Supplementary Table S1.

### 2.2. Short Tandem Repeat (STR) Profiling and Line Authentication

To confirm the identity of each donor-derived line and ensure their stable representation within pooled neuronal cultures, short tandem repeat (STR) profiling was performed on both PSP-RS and HC samples at the beginning of differentiation (DIV 0) and at the final stage (DIV 60). STR analysis was carried out by Eurofins Genomics Europe (Biotech Products & Services GmbH, Ebersberg, Germany). Genomic DNA was extracted and subjected to multiplex PCR amplification targeting 16 STR loci using the AmpFlSTR Identifiler^®^ Plus Kit (Thermo Fisher Scientific), following the manufacturer’s instructions. The panel included the following markers: D8S1179, D21S11, D7S820, CSF1PO, D3S1358, TH01, D13S317, D16S539, D2S1338, AMEL (amelogenin), D5S818, FGA, D19S433, vWA, TPOX, and D18S51. STR profiles obtained at DIV60 were directly compared with those generated from the corresponding parental iPSC profiles at DIV 0 to verify sample identity and exclude potential cross-contamination or disproportionate expansion of individual donor-derived lines within pooled cultures.

### 2.3. Immunofluorescence Analysis

At selected time points, neuronal cultures were processed for immunofluorescence staining. Cells were fixed in 4% formaldehyde (FA; Sigma-Aldrich) for 15 min at room temperature (RT) and subsequently washed with PBS (+/+) (Corning). To permeabilize and block non-specific binding, samples were incubated in PBS (+/+) containing 10% goat serum (Thermo Fisher Scientific) and 0.1% Triton X-100 (Sigma-Aldrich). Primary antibodies were applied overnight at 4 °C in a reduced-serum blocking solution. Following washing steps, cells were incubated with fluorophore-conjugated secondary antibodies for 1 h at RT. Nuclei were counterstained with DAPI (1:1000; Carl Roth, Karlsruhe, Germany), and coverslips were mounted using DAKO Fluorescent Mounting Medium (Agilent, Santa Clara, CA, USA). For the in situ detection of apoptosis, 150,000 neurons were seeded onto 24-well slides and processed using the Click-iT™ Plus TUNEL Assay Kit Alexa Fluor™ 594 (Thermo Fisher Scientific, Waltham, MA, USA), following the manufacturer’s instructions. Images were acquired using a Leica DMi8 inverted microscope (LAS X v3.7.4.23463) and a Leica MICA microscope (LAS X v6.2.2.28360; Leica Microsystems CMS GmbH, Wetzlar, Germany). Quantitative analysis was performed using ImageJ software (v1.54s) [36,37]. Colocalization analysis was conducted using the JaCoP plugin [38]. A list of the antibodies used is provided in Supplementary Table S1.

### 2.4. Protein Extraction and Western Blot Analysis

Cells were collected in 1× PBS (−/−) (Corning) and lysed in RIPA buffer (50 mM Tris-HCl, pH 7.5; 150 mM NaCl; 1% Triton X-100; 0.5% sodium deoxycholate; 0.1% SDS; Sigma-Aldrich, St. Louis, MO, USA) supplemented with protease and phosphatase inhibitors (Thermo Fisher Scientific). Lysates were sonicated using a Diagenode Bioruptor (Diagenode, Seraing, Belgium, 3 cycles of 20 s on/off), incubated on ice for 30 min, and clarified by centrifugation at 21,000× *g* for 1 h at 4 °C. Protein concentration was determined using the Bradford assay (Bio-Rad, Hercules, CA, USA). Equal amounts of protein (20 µg) were mixed with LDS sample buffer and reducing agent (Bolt™ or NuPAGE™ Sample Reducing Agent; Thermo Fisher Scientific), denatured at 70 °C for 10 min, separated by SDS–PAGE, and transferred onto nitrocellulose membranes (Bio-Rad, Hercules, CA, USA). Membranes were blocked in 5% non-fat milk prepared in TBS-T for 1 h at room temperature (RT) and incubated overnight at 4 °C with primary antibodies. After washing, membranes were incubated with HRP-conjugated secondary antibodies (1:10,000; Jackson ImmunoResearch, West Grove, PA, USA) and developed using chemiluminescence reagents (Clarity™ ECL substrate, Bio-Rad). Signal detection was performed using an Alliance™ Q9-Atom imaging system, and band intensities were quantified with ImageJ. GAPDH and histone H3 served as loading controls. A list of primary and secondary antibodies is in Supplementary Table S1. Uncropped Western blot images are available in Supplementary File S1.

### 2.5. Transcriptome Profiling by Bulk RNA-Sequencing

At DIV 60, total RNA was extracted from pooled mDA neurons from HC and PSP-RS iPSC lines using TRIzol™ Reagent (Thermo Fisher Scientific), following standard protocols. RNA quantity and integrity were evaluated using the Agilent 2100 Bioanalyzer (Agilent). Polyadenylated transcripts were selectively enriched with poly(T) magnetic beads and subsequently fragmented prior to cDNA synthesis. First-strand cDNA synthesis was generated using random hexamer primers, followed by second-strand synthesis incorporating dUTP to maintain strand specificity. Library preparation quality and concentration were assessed using Qubit fluorometry (Thermo Fisher Scientific), quantitative PCR (qPCR), and Bioanalyzer analysis (Agilent). Sequencing was carried out on an Illumina NextSeq 1000 platform. Sequencing reads were aligned to the human reference genome (GRCh38) using HISAT2 (v2.0.5). Transcript reconstruction was performed with StringTie (v1.3.3b), and gene-level read counts were generated using FeatureCounts (v1.5.0-p3). Expression values were normalized as fragments per kilobase of transcript per million mapped reads (FPKM). Differential expression analysis was conducted on raw counts using DESeq2 (v1.20.0). Statistical significance was determined after adjustment for multiple testing using the Benjamini–Hochberg false discovery rate (FDR) method, with genes meeting the criteria of adjusted *p* ≤ 0.05 and |log_2_ fold change| ≥ 1 considered significantly differentially expressed. Functional enrichment and Gene Ontology (GO) analyses were performed using the ClusterProfiler R package (*p* < 0.05). Gene Set Enrichment Analysis (GSEA) was carried out using a local implementation of the Gene Set Analysis (GSA) tool, applying a significance threshold of *p* < 0.05.

### 2.6. Proteomic Sample Preparation and Mass Spectrometry

At DIV 60, quantitative proteomic analysis was performed using four independent replicates per condition. Cell lysates were prepared as described above. Protein samples were first subjected to reduction with 100 mM dithiothreitol (DTT) for 1 h at 37 °C, followed by alkylation with 200 mM iodoacetamide under the same conditions. Residual iodoacetamide was neutralized by an additional incubation with 100 mM DTT for 20 min at 37 °C. For downstream processing, 10 µg of protein per sample were handled using the Protein Aggregation Capture (PAC) method [39]. Protein binding to MagReSyn^®^ Hydroxyl beads was induced by adjusting the acetonitrile to 70% and incubating for 10 min at 1100 rpm. The beads were subsequently washed three times with acetonitrile and once with 70% ethanol, then resuspended in 50 mM triethylammonium bicarbonate (TEAB). Proteolytic digestion was performed overnight at 37 °C using Trypsin/Lys-C at a 1:50 (*w*/*w*) enzyme-to-substrate ratio. Peptides were eluted from the beads using 0.1% formic acid. Peptide mixtures were analyzed by liquid chromatography–tandem mass spectrometry (LC–MS/MS) using an Orbitrap Exploris 480 mass spectrometer (Thermo Fisher Scientific) equipped with a 17 cm × 75 µm capillary column packed with 3 µm C18 silica particles. Separation was achieved using a 140 min binary gradient at a flow rate of 300 nL/min, with mobile phase B (80% acetonitrile, 0.1% formic acid) increasing from 3% to 40%, followed by a high-organic wash. Data acquisition was performed in data-independent acquisition (DIA) mode, using 30 variable isolation windows spanning an m/z range of 350–1010. Full MS scans were acquired at a resolution of 60,000, while DIA scans were collected at a resolution of 30,000. Raw data were processed with Spectronaut (v18.7) using the Human 1 Protein 1 Gene database (March 2022 release). Only protein groups detected in at least 40% of runs were retained (thus, at least 4 runs out of 8), and missing values were imputed based on the background signal. Signal intensities were normalized using the default local normalization setting in Spectronaut. Further statistical analysis was conducted in Perseus (v2.0.11.0). Protein intensities were log_2_-transformed and filtered to retain proteins quantified in at least three replicates per group. Differential expression was assessed using a two-sample *t*-test with permutation-based false discovery rate (FDR ≤ 0.05; S_0_ = 0.2), resulting in the identification of 1479 significantly regulated proteins. Gene Ontology (GO) enrichment analysis was performed using the ClusterProfiler R package (version R.4.4.1) (enrichGO function; keyType = “SYMBOL”), with multiple testing corrections applied using the Benjamini–Hochberg method. Enrichment terms were classified into Biological Process (BP), Cellular Component (CC), and Molecular Function (MF) categories. Functional networks were visualized using Metascape [40], where nodes represent enriched terms and edges indicate gene overlap (similarity score > 0.3), allowing clustering of related biological processes. Proteomics data have been deposited in the ProteomeXchange Consortium [41] via the PRIDE repository [42]. Unless otherwise stated, all reagents were obtained from Sigma-Aldrich (St. Louis, MO, USA).

### 2.7. mTOR Inhibition Assays

To investigate the functional role of mTOR signaling, PSP-RS–derived midbrain dopaminergic (mDA) neurons were treated with pharmacological inhibitors for 24 h. Cells were treated with either 100 nM Torin-1 (Sigma-Aldrich, #475991) or 1 µM rapamycin (Selleck Chemicals, #S1039, Houston, TX, USA). Following treatment, neuronal cultures were harvested and subjected to protein extraction for subsequent Western blot analysis, as described above. Activation of the mTOR pathway was evaluated by measuring the phosphorylation status of mTOR at Ser2448, which serves as an established readout of pathway activity [43].

### 2.8. Statistical Analysis

All statistical analyses were carried out using GraphPad Prism software (version 10.6.1; GraphPad Software Inc., San Diego, CA, USA). In this study, replicates correspond to independent differentiation experiments performed at separate time points using the same pooled iPSC lines. Details regarding the statistical tests employed, sample size (N), definition of replicates, and measures of variability are provided in the corresponding figure legends. For transcriptomic and proteomic analyses, the statistical approaches are described in the corresponding sections of the Materials and Methods.

## 3. Results

### 3.1. PSP-RS Neurons Exhibit Dopaminergic Neuron Loss and Early Synaptic Vulnerability

**Figure 1:**
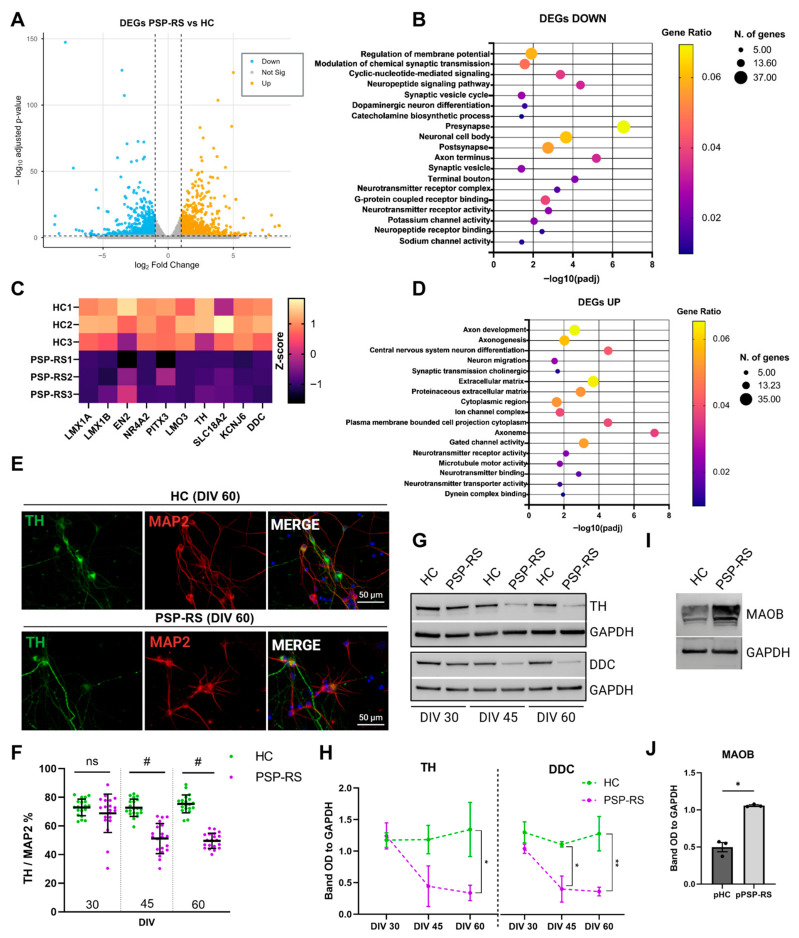
Transcriptional dysregulation and dopaminergic impairments in PSP-RS midbrain neurons. (**A**), Volcano plot showing differentially expressed genes (DEGs) between PSP-RS and HC mDA neurons (DIV 60). Thresholds: |log_2_FC| ≥ 1 and adjusted *p* ≤ 0.05 (DESeq2). (**B**), Gene Ontology (GO) enrichment analysis of significantly downregulated DEGs in PSP-RS versus HC mDA neurons. Dot size indicates the number of genes per term, and the color scale represents the gene ratio. (**C**), Heatmap showing normalized expression (Z-score) of key dopaminergic markers across individual replicates (independent differentiation experiments) of HC and PSP-RS neurons. (**D**), Gene Ontology (GO) enrichment analysis of significantly upregulated DEGs in PSP-RS versus HC mDA neurons. Dot size indicates the number of genes per term, and the color scale represents the gene ratio. (**E**), Representative immunofluorescence images of TH (green) and MAP2 (red) in HC and PSP-RS mDA neurons (DIV 60). Nuclei are counterstained with DAPI (blue). Scale bar, 50 μm. (**F**), Quantification of TH^+^/MAP2^+^ neurons during differentiation (DIV 30–60). Data are presented as mean ± s.e.m.; *N* = 20 images (dots) per group derived from three independent differentiation experiments; a minimum of 300 neurons were analyzed per condition; ns = *p* > 0.05, # *p* ≤ 0.0001 (unpaired *t*-test with Welch’s correction). (**G**), Representative Western blots of TH and DDC protein levels in HC and PSP-RS mDA neurons at DIV 30, 45 and 60; GAPDH was used as a loading control. (**H**), Densitometric quantification of TH and DDC bands shown in (**G**). Data are presented as mean ± s.e.m. of three independent differentiation experiments; * *p* ≤ 0.05, ** *p* ≤ 0.01 (unpaired *t*-test with Welch’s correction). (**I**), Representative Western blots of MAOB protein levels in HC and PSP-RS mDA neurons at DIV 60; GAPDH was used as a loading control. (**J**), Densitometric quantification of MAOB bands normalized to GAPDH. Data are presented as mean ± s.e.m. of three independent differentiation experiments; * *p* ≤ 0.05 (unpaired *t*-test with Welch’s correction).

Midbrain dopaminergic (mDA) neurons were generated from hiPSCs derived from three individuals with sporadic PSP-RS and three matched healthy controls (Supplementary Table S2). All lines produced comparable proportions of FOXA2/LMX1A-positive midbrain floor plate progenitors at DIV 11 (Supplementary Figure S1A–D) and generatedTH/DDC-positive and TH/GIRK2-positive neurons at later differentiation stages (DIV 45 and DIV 60) (Supplementary Figure S1E). To further assess differentiation dynamics, we monitored TH expression relative to DAPI at multiple time points (DIV 20, DIV 30, DIV 45, and DIV 60). While control lines showed a progressive increase in TH/DAPI ratios over time, PSP-RS neurons exhibited a decline at later stages, despite comparable levels at earlier time points (DIV 20 and DIV 30), indicating dopaminergic neuron loss (Supplementary Figure S1F). Given the comparable differentiation efficiency within each group, equal numbers of progenitors from each donor were pooled at DIV 20 to generate PSP-RS and control populations. Replicates consisted of independent differentiation experiments derived from the same pooled progenitor mixture. STR profiling confirmed stable representation of each donor line throughout differentiation (Supplementary Table S3). Pooled cultures recapitulated the expected stage-specific progression of mDA markers, including NURR1 at DIV 30, DDC at DIV 45, and GIRK2 at DIV 60 (Supplementary Figure S2A–C), consistent with observations in individual lines. While PSP-RS and control neurons exhibit comparable differentiation efficiency at early stages, they undergo progressive divergence during maturation, as evidenced by the distinct transcriptomic segregation revealed by bulk RNA sequencing (Supplementary File S2). Principal component analysis (PCA) and Pearson correlation analyses confirmed distinct group-specific expression profiles (Supplementary Figure S2D,E). Among 35,602 detected transcripts, 1415 were differentially expressed, including 678 downregulated and 737 upregulated genes in PSP-RS neurons (Figure 1A). Downregulated genes were enriched in biological processes related to membrane potential regulation, synaptic transmission, dopaminergic differentiation, and catecholamine metabolism, with significant depletion of components localized to axon terminals and synaptic compartments. Additionally, pathways associated with neuronal signaling and neuroprotection, including GPCR and neuropeptide receptor interactions, were reduced (Figure 1B). Notably, key regulators of dopaminergic identity and function, including LMX1A/1B, EN2, NR4A2 (NURR1), PITX3, LMO3, TH, SLC18A2 (VMAT2), KCNJ6 (GIRK2), and DDC, were significantly downregulated in PSP-RS neurons (Figure 1C). In parallel, upregulated transcripts were enriched in pathways associated with axonal development, extracellular matrix organization, and microtubule-related processes, consistent with altered structural homeostasis (Figure 1D). At the protein level, immunofluorescence analysis revealed a progressive reduction in TH-positive neurons relative to MAP2-positive cells in PSP-RS cultures (Figure 1E,F). This observation was further supported by Western blot analysis showing comparable TH and DDC levels between HC and PSP-RS neurons at DIV 30, followed by a decrease in PSP-RS neurons at DIV 45 and DIV 60, and increased expression of MAOB at DIV 60 in PSP-RS neurons (Figure 1G–J). To investigate whether the reduction in neuronal number involved apoptosis, we performed TUNEL assay analysis, which confirmed increased TUNEL-positive cells in PSP-RS dopaminergic neurons compared to controls (Supplementary Figure S2F). Together, these findings demonstrate that, despite normal acquisition of dopaminergic fate, PSP-RS neurons undergo selective dopaminergic neuron loss due to cell death and exhibit early features of synaptic dysfunction, accompanied by a shift toward cytoskeletal and structural remodeling.

### 3.2. Proteomic Profiling Reveals Coordinated Synaptic Depletion and Structural Remodeling in PSP-RS Neurons

**Figure 2:**
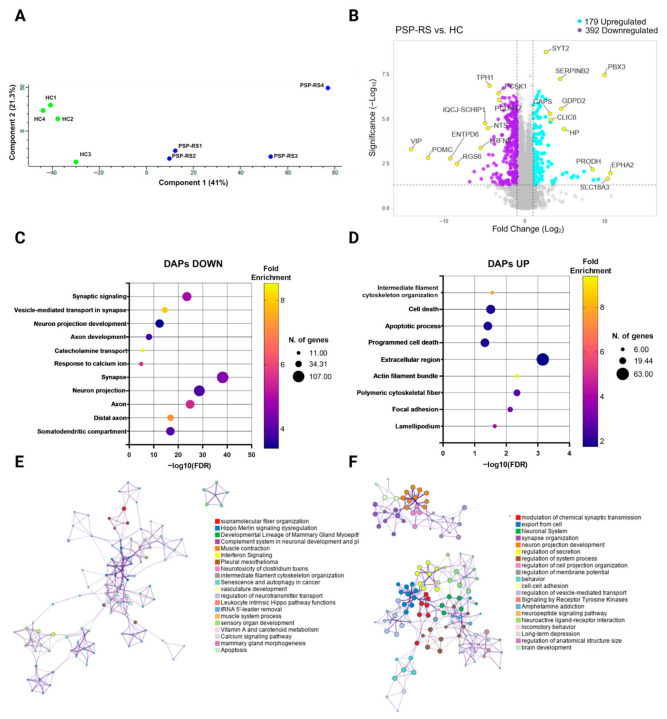
Proteomic profiling reveals molecular alterations in PSP-RS midbrain dopaminergic neurons. (**A**), Principal component analysis (PCA) of proteomic profiles from HC and PSP-RS mDA neurons at DIV 60. (**B**), Volcano plot of differentially abundant proteins (DAPs) between PSP-RS and HC mDA neurons. Thresholds: |log_2_ fold change| ≥ 1 and q ≤ 0.05. (**C**,**D**), Gene Ontology (GO) enrichment analysis of significantly downregulated (**C**) and upregulated (**D**) DAPs in PSP-RS versus HC mDA neurons. Dot size represents the number of proteins per GO term; color scale indicates fold enrichment. (**E**,**F**), Network visualization of enriched GO terms for upregulated (**E**) and downregulated (**F**) DAPs, illustrating functional clustering of related biological processes.

Proteomic profiling at DIV 60 confirmed and extended transcriptomic findings, revealing a coordinated shift from synaptic maintenance toward structural remodeling in PSP-RS neurons. Quantitative proteomic analysis identified a total of 6418 proteins across all samples (Supplementary File S3). Principal component analysis (PCA) demonstrated a clear separation between PSP-RS and HC groups (Figure 2A), indicating distinct proteomic profiles. Among detected proteins, 571 were differentially abundant (|log_2_FC| ≥ 1, *p* ≤ 0.05), including 392 downregulated and 179 upregulated proteins in PSP-RS neurons (Figure 2B). Downregulated proteins were enriched in pathways related to synaptic transmission, neuron projection development, and catecholamine metabolism (Figure 2C), consistent with impaired maintenance of dopaminergic and synaptic programs. In contrast, upregulated proteins were associated with cytoskeletal organization and cell death-associated pathways (Figure 2D). These results are consistent with transcriptomic findings, supporting the robustness of the observed phenotype across independent omics layers. Notably, enrichment of GO terms associated with cell death and apoptotic processes supports the observed reduction in dopaminergic neurons. Network analysis revealed interconnected modules involving supramolecular fiber organization, intermediate filament cytoskeleton assembly, regulation of neurotransmitter transport, calcium signaling, and apoptotic pathways (Figure 2E), supporting activation of structural remodeling and stress-response programs. Conversely, functional clusters associated with synaptic transmission, synapse organization, neuron projection development, and neuropeptide signaling were significantly depleted in PSP-RS neurons (Figure 2F). Although donor-specific variability cannot be resolved due to the pooled experimental design, the convergence of transcriptomic and proteomic alterations supports a coherent multi-omics phenotype characterized by death of dopaminergic neurons and early structural destabilization in PSP-RS neurons. Together, these findings indicate that PSP-RS neurons exhibit an early shift in cellular state, characterized by progressive loss of synaptic and dopaminergic functions and a concomitant increase in cytoskeletal and stress-related processes.

### 3.3. PSP-RS Neurons Exhibit Disease-Relevant Tau Phosphorylation

**Figure 3:**
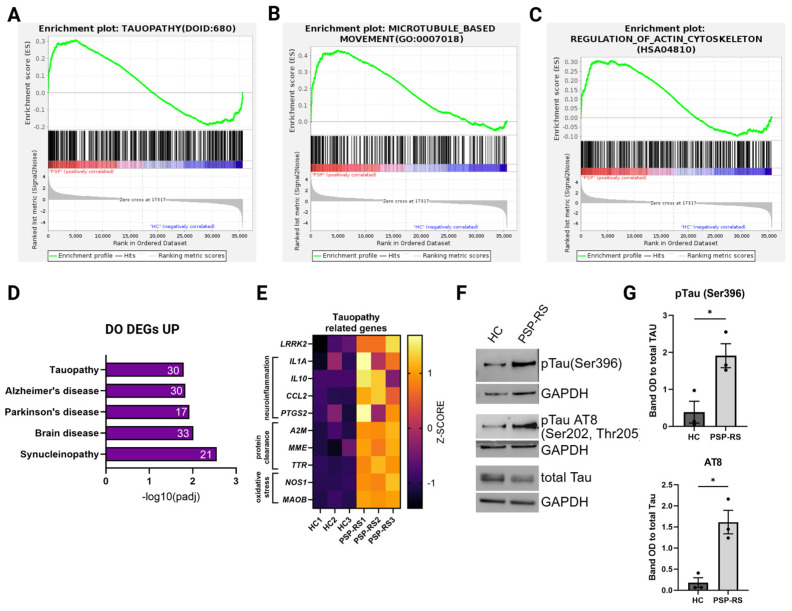
Pathological features of PSP-RS midbrain dopaminergic neurons. (**A**–**C**), Gene Set Enrichment Analysis (GSEA) showing positive enrichment of genes associated with tauopathy (**A**), microtubule −based movement (**B**), and regulation of the actin cytoskeleton (**C**) in PSP −RS compared to HC mDA neurons (DIV 60). (**D**), Disease Ontology (DO) analysis of upregulated DEGs in PSP-RS neurons at DIV 60, highlighting enrichment of pathways linked to multiple neurodegenerative disorders, including Tauopathy, Alzheimer’s, and Parkinson’s disease. (**E**), Heatmap showing normalized expression (Z-score) of tauopathy-related genes, identified by bulk RNA−sequencing, across individual replicates (independent differentiation experiments) of HC and PSP−RS neurons (DIV 60). (**F**), Representative Western blots of total and phosphorylated Tau (pTau Ser396 and pTau AT8 [Ser202/Thr205]) in HC and PSP-RS mDA neurons at DIV 60; GAPDH served as a loading control. (**G**), Densitometric quantification of pTau (Ser396) and pTau (AT8) bands normalized to total Tau level. Data are presented as mean ± s.e.m. of three independent differentiation experiments; * *p* ≤ 0.05 (unpaired *t*-test with Welch’s correction).

To assess whether PSP-RS–derived mDA neurons exhibit disease-associated Tau alterations, we analyzed both transcriptional and protein-level changes related to Tau pathology. GSEA revealed significant enrichment of tauopathy-related signatures (NES = 1.815), together with cytoskeleton-associated pathways, including microtubule-based movement (NES = 1.95) and regulation of the actin cytoskeleton (NES = 1.49) (Figure 3A–C). Disease Ontology analysis further demonstrated enrichment of gene sets associated with neurodegenerative disorders, supporting the presence of disease-relevant molecular signatures (Figure 3D). Among upregulated genes within tauopathy-related categories, LRRK2, a kinase implicated in Tau phosphorylation [44], was significantly increased. Additional transcriptional changes involved genes related to inflammatory signaling (IL1A, IL10, CCL2, and PTGS2), protein clearance (A2M, MME, and TTR), and oxidative stress (NOS1 and MAOB), suggesting activation of complementary stress-response pathways (Figure 3E). At the protein level, PSP-RS neurons exhibited increased phosphorylation of Tau at Ser396 and at the AT8 epitope (Ser202/Thr205), normalized to total Tau, consistent with enhanced disease-relevant Tau modification (Figure 3F,G). Collectively, these findings indicate that PSP-RS–derived neurons recapitulate early, disease-relevant alterations in Tau phosphorylation in the absence of overt degeneration, supporting the use of this model to investigate pre-degenerative Tau dysregulation.

### 3.4. Tau Dysregulation Is Associated with Axonal Structural Remodeling and Neurofilament Accumulation in PSP-RS Neurons

**Figure 4:**
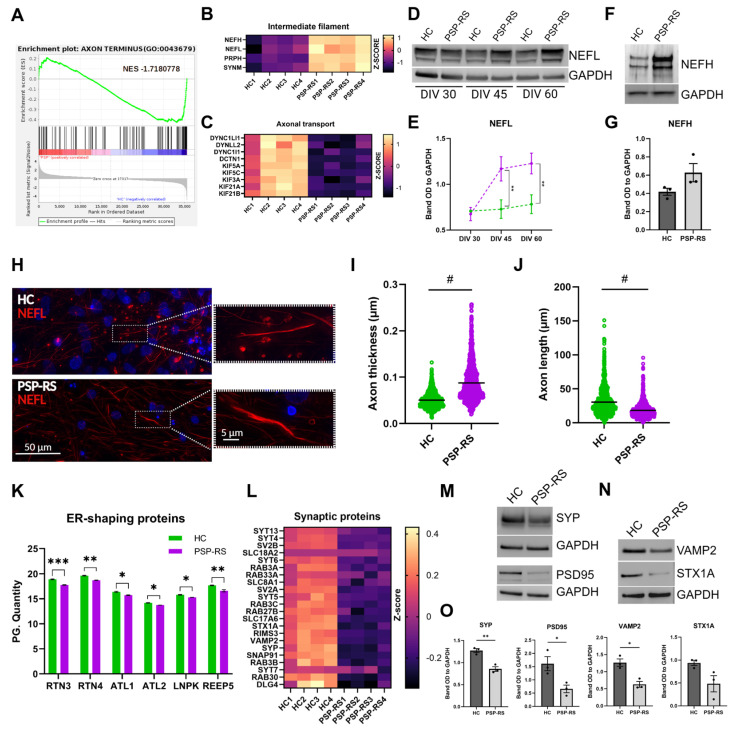
Axonal injury and neurofilament elevation in PSP-RS midbrain dopaminergic neurons. (**A**), Gene Set Enrichment Analysis (GSEA) showing negative enrichment of genes associated with the axon terminus in PSP−RS compared to HC mDA neurons (DIV 60). (**B**,**C**), Heatmaps displaying Z-score-normalized expression of proteins related to intermediate filaments (**B**) and axonal transport (**C**), as identified by proteomic analysis. Each column represents a single replicate (independent differentiation experiments, DIV 60). (**D**), Representative Western blots of NEFL protein levels in HC and PSP−RS mDA neurons at DIV 30, 45 and 60; GAPDH was used as a loading control. (**E**), Densitometric quantification of NEFL bands shown in (**D**). (**F**,**G**), Representative Western blots (**F**) and densitometric quantification (**G**) of NEFH protein levels normalized to GAPDH in HC and PSP−RS neurons at DIV 60. (**H**), Representative immunofluorescence images of NEFL (red) in HC and PSP−RS neurons. Nuclei are counterstained with DAPI (blue). Scale bars, 50 µm (left) and 5 µm (right). (**I**,**J**), Quantification of axonal thickness (**I**) and axonal length (**J**) in HC and PSP−RS neurons at DIV 60, *N* = 1000 axons per group. Data are shown as mean ± s.e.m.; # *p* ≤ 0.0001 (unpaired *t*-test with Welch’s correction). (**K**), Quantification of endoplasmic reticulum (ER)-shaping proteins measured by mass spectrometry proteomic analysis in mDA neurons (DIV 60). Data represent mean ± s.e.m.; * *p* ≤ 0.05, ** *p* ≤ 0.01, *** *p* ≤ 0.001, *N* = 4, two-sample *t*-test with a permutation-based FDR. (**L**), Heatmap of synaptic proteins across replicates, expressed as Z-score-normalized expression, as identified by proteomic analysis. (**M**–**O**), Representative Western blots (**M**,**N**) and densitometric quantification (**O**) of synaptic proteins SYP, PSD95, VAMP2, and STX1A normalized to GAPDH. For (**E**,**G**,**O**), data are presented as mean ± s.e.m. of three independent differentiation experiments; * *p* ≤ 0.05, ** *p* ≤ 0.01 (unpaired *t*-test with Welch’s correction).

Given the established role of hyperphosphorylated Tau in microtubule destabilization [9], we next examined whether Tau alterations in PSP-RS were associated with structural changes in the axonal compartment. GSEA revealed significant depletion of transcripts associated with the “axon terminus”, indicating early vulnerability of distal axonal domains (Figure 4A). Proteomic profiling demonstrated increased abundance of intermediate filament proteins, including neurofilament heavy and light chain (NEFH, NEFL), peripherin (PRPH), and synemin (SYNM) (Figure 4B), consistent with altered cytoskeletal composition [45,46]. In parallel, multiple components of the axonal transport machinery, including dynein, dynactin subunits, and several kinesins, were significantly reduced (Figure 4C), suggesting perturbation of intracellular transport systems. Immunoblot analysis of NEFL revealed a temporal pattern of axonal dysfunction, with significant increases in PSP-RS neurons at DIV 45 and 60 compared to controls (Figure 4D,E), whereas analysis of NEFH at Day 60 showed a trend toward increased levels in PSP-RS neurons, though not reaching statistical significance (Figure 4F,G). Morphometric analysis further revealed increased axonal thickness and reduced axonal length in PSP-RS (Figure 4H–J; Supplementary Figure S3A), consistent with early structural remodeling of axons [47]. Because axonal integrity critically depends on endoplasmic reticulum (ER) architecture, we next examined ER-shaping proteins and observed reduced expression of RTN3, RTN4, ATL1, ATL2, LNPK, and REEP5 (Figure 4K), indicating impaired maintenance of ER structure within neurites. Consistent with axonal dysfunction, dopaminergic synaptic pathways were significantly downregulated (NES = −1.484457, Supplementary Figure S3B), and multiple synaptic proteins were reduced (Figure 4L). Decreased expression of SYP, DLG4 (PSD95), VAMP2, and STX1A was confirmed by immunoblotting, with reductions in SYP, PSD95, and VAMP2 reaching statistical significance (Figure 4M–O). Reduced SYP and PSD95 levels in TH-positive neurons were further confirmed by immunofluorescence (Supplementary Figure S3C–F). Together, these findings indicate that Tau dysregulation in PSP-RS neurons is associated with coordinated alterations in intermediate filaments, axonal transport machinery, ER structural regulators, and synaptic components. Although direct functional impairment of axonal transport and compartment-specific neurofilament dynamics remain to be established, the convergence of these alterations supports the presence of an early axonopathic phenotype characterized by cytoskeletal imbalance and structural vulnerability. Together, these findings suggest early disruption of axonal structural integrity rather than late stage degenerative change.

### 3.5. mTOR Signaling Contributes to Tau Phosphorylation and Neurofilament Accumulation in PSP-RS Neurons

**Figure 5:**
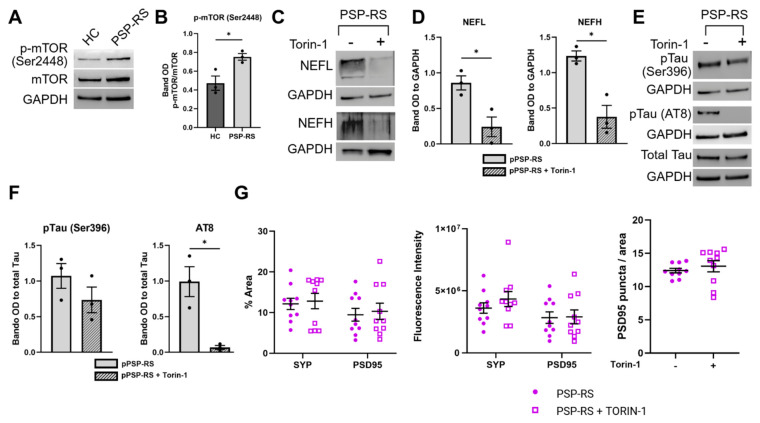
mTOR hyperactivation drives cytoskeletal protein accumulation in PSP-RS mDAs. (**A**,**B**), Representative Western blot (**A**) and densitometric quantification (**B**) of phosphorylated mTOR (p-mTOR Ser2448) normalized to total mTOR in HC and PSP-RS midbrain mDA neurons at DIV 60. (**C**,**D**), Representative Western blot (**C**) and densitometric quantification (**D**) of NEFL, and NEFH bands in PSP-RS mDA neurons at DIV60 after 24 h of Torin-1 treatment at 100 nM. (**E**,**F**), Representative Western blot (**E**) and densitometric quantification (**F**) of phosphorylated Tau (pTau Ser396 and pTau AT8 [Ser202/Thr205]) in PSP-RS mDA neurons at DIV60 after 24 h of Torin-1 treatment at 100 nM. For (**D**,**F**), data are presented as mean ± s.e.m. of three independent differentiation experiments; * *p* ≤ 0.05, (unpaired *t*-test with Welch’s correction). (**G**), Quantification of percentage area coverage, and fluorescence intensity of SYP and PSD95 signals, and PSD95 puncta density (puncta per unit area, a.u.), in PSP-RS mDA neurons at DIV60 after 24 h of Torin-1 treatment. Data are mean ± s.e.m.; *N* = 10 images per group derived from three independent differentiation experiments.

To investigate whether signaling pathways contribute to the observed cytoskeletal alterations, we focused on mTOR signaling, a central regulator of protein synthesis, cytoskeletal dynamics, and neuronal homeostasis [48,49,50]. Although mTOR was not among the top pathways identified in unbiased omics analyses, its known role in coordinating proteostasis and structural homeostasis prompted further functional investigation. Western blot analysis revealed increased phosphorylation of mTOR at Ser2448 in PSP-RS neurons compared with controls (Figure 5A,B), indicating enhanced pathway activity. To assess whether mTOR activity functionally contributes to the cytoskeletal alterations observed in PSP-RS neurons, we performed pharmacological inhibition experiments (Supplementary Figure S4A,B). Treatment with the mTOR inhibitor Torin-1 significantly reduced neurofilament levels, including both NEFL and NEFH, and decreased Tau phosphorylation at Ser396 and at AT8, with the latter reaching statistical significance (Figure 5C–F). While Torin-1 treatment resulted in a slight increase in synaptic proteins (SYP and PSD95), the changes were not statistically significant (Figure 5G). Similar effects were observed following treatment with rapamycin (Supplementary Figure S4C,D), supporting pathway specificity. While these data do not support mTOR as a primary driver of pathology, these findings indicate that mTOR signaling functionally modulates key components of the cytoskeletal phenotype.

## 4. Discussion

In this study, we identify a cytoskeletal stress axis in sporadic PSP-RS that is functionally modulated by mTOR signaling. Using iPSC-derived midbrain dopaminergic neurons, we show that Tau hyperphosphorylation, neurofilament accumulation, and axonal structural remodeling converge early to define a pre-degenerative phenotype. Although PSP is a 4R Tauopathy, iPSC-derived neurons display an immature tau splicing pattern with low 4R isoforms. While this limits modeling of isoform-specific pathology, it does not preclude the investigation of early, potentially isoform-independent mechanisms of Tau-driven axonal dysfunction. Integrated transcriptomic and proteomic analyses revealed coordinated attenuation of dopaminergic and synaptic programs together with increased expression of intermediate filament components and reduced levels of axonal transport machinery. A key finding of this study is that these alterations emerge in the absence of overt neurodegeneration, supporting the concept that axonal dysfunction represents an early and potentially primary event in PSP-RS. This is consistent with growing evidence across tauopathies indicating that disruption of axonal integrity precedes neuronal loss [51,52,53]. Midbrain dopaminergic neurons are intrinsically vulnerable due to their extensive axonal arborization, high metabolic demand, and reliance on tightly regulated intracellular transport [31,54,55]. In this context, even subtle perturbations in cytoskeletal organization or trafficking efficiency may critically lower the threshold for dysfunction. Our multi-omics analyses reveal a coordinated shift toward structural remodeling and stress-response pathways. Within this framework, Tau hyperphosphorylation likely represents an initiating event, as pathological Tau modifications are known to destabilize microtubules and impair axonal transport [56,57] and its accumulation within axonal compartments is supported by neuropathological evidence [58,59]. In our model, increased Tau phosphorylation occurred alongside coordinated alterations in axonal transport components, including kinesin, dynein, and dynactin subunits [14], as well as accumulation of intermediate filament proteins, suggesting a broader disruption of cytoskeletal homeostasis. Importantly, our data suggest that neurofilament accumulation may represent more than a passive biomarker of axonal injury. While NEFL is widely used as a clinical marker of neurodegeneration [18,19,20,60], the intracellular accumulation observed here indicates an active remodeling of axonal cytoskeletal composition. Whether these alterations represent primary drivers or secondary responses remains to be determined; however, impaired proteostatic mechanisms may contribute to their persistence and exacerbate transport instability [61,62,63,64]. In this context, we identify mTOR signaling as a modulatory pathway contributing to the maintenance of cytoskeletal imbalance. While mTOR activity is essential for neuronal growth and synaptic plasticity under physiological conditions [65,66], sustained activation has been linked to impaired proteostasis and protein aggregation in neurodegenerative diseases [67]. Mechanistically, excessive mTOR activity can promote Tau hyperphosphorylation through activation of Tau kinases such as p70S6K and inhibition of phosphatases including PP2A [67,68,69,70]. Consistent with this, increased mTOR pathway activity correlates with phospho-Tau burden in human neurodegenerative disease brains [70], and mTOR inhibition attenuates aggregate-associated pathology in experimental models [71,72,73]. In our model, increased mTOR activity is associated with enhanced Tau phosphorylation and neurofilament accumulation, and its pharmacological inhibition attenuates both processes. These findings do not support mTOR as a primary driver of pathology but instead indicate that it acts as a pathogenic amplifier, sustaining cytoskeletal stress and reinforcing axonal vulnerability. Overall, our findings support a model in which early axonopathy in PSP-RS arises from the convergence of Tau dysregulation and impaired axonal homeostasis. By linking signaling dysregulation to structural axonal imbalance in a human patient-derived system, this study provides mechanistic insight into pre-degenerative processes in sporadic PSP-RS. These data highlight mTOR-related pathways as potential modulators of early axonal disease progression. Notably, the potential relevance of mTOR modulation may extend beyond PSP-RS, as accumulating evidence suggests that different PSP subtypes, including PSP-P, share core Tau-related pathogenic mechanisms despite their clinical heterogeneity, raising the possibility that targeting mTOR-dependent pathways could have broader therapeutic applicability across the PSP spectrum [74]. Future studies will be required to define the temporal hierarchy of these events and to determine whether modulation of mTOR signaling can effectively mitigate axonal vulnerability in disease-relevant contexts.

## 5. Conclusions

In summary, PSP-RS–derived midbrain dopaminergic neurons exhibit a coordinated molecular and structural phenotype characterized by increased Tau phosphorylation, neurofilament accumulation, and cytoskeletal remodeling, accompanied by enhanced mTOR signaling activity. These alterations define an early axonopathic state in a human cellular model of sporadic PSP-RS. Our findings support a model in which cytoskeletal dysfunction arises from the convergence of Tau dysregulation and axonal imbalance. In this context, mTOR signaling emerges as a modulatory pathway associated with these alterations and contributing to their maintenance, rather than acting as a primary driver of pathology. Overall, this study provides mechanistic insight into early disease-associated processes and identifies cytoskeletal homeostasis as a critical axis in PSP-RS pathogenesis, with potential implications for therapeutic targeting.

## 6. Limitations

A key methodological aspect of this study is the use of pooled midbrain floor plate progenitors derived from multiple PSP-RS and control donors. While this strategy reduces clonal variability and enhances the detection of shared disease-associated signals, it limits the ability to resolve donor-specific heterogeneity and inter-individual variability. In our experimental design, replicates correspond to independent differentiation experiments performed at separate time points using the same pooled iPSC lines. Future studies using individual iPSC lines will be required to assess the reproducibility and variability of the observed phenotypes across distinct PSP-RS donors. In addition, although our data reveal coordinated alterations in cytoskeletal composition and axonal transport–related proteins, direct functional assessment of axonal transport dynamics and compartment-specific protein distribution was not performed. Such analyses will be essential to establish the functional consequences of the observed molecular changes and to further define their mechanistic relationships. Finally, while pharmacological modulation of mTOR signaling impacts Tau phosphorylation and neurofilament accumulation, the causal hierarchy between mTOR activation and cytoskeletal alterations remains to be fully established. Future studies will be necessary to determine whether mTOR represents a viable and disease-modifying therapeutic target in PSP-RS. Additionally, as iPSC-derived neurons retain a developmentally immature Tau splicing profile with limited 4R isoform expression, this model may not fully capture isoform-specific aspects of PSP pathology.

## Data Availability

The raw RNAseq dataset supporting the conclusions of this article has been deposited in the Gene Expression Omnibus (GEO) database, under accession numbers GSE304324 (HC) and GSE313924 (PSP-RS). The proteomics data are available via ProteomeXchange with identifiers PXD066765 (HC) and PXD072104 (PSP-RS). The other datasets are available from the corresponding author on reasonable request.

## Supplementary Materials

The following supporting information can be downloaded at: https://www.mdpi.com/article/10.3390/cells15090754/s1, File S1: Uncropped Western blot images; File S2: RNA-seq data; File S3: Mass spectrometry-based data; Table S1: Antibodies, medium and small molecules; Table S2: Demographic information of cell line donors; Table S3: STR profile; Figures and legends (Figures S1–S4).

## Abbreviations

Abbreviation	Meaning
BP	Biological Process
CC	Cellular Component
MF	Molecular Function
DEGs	Differentially expressed genes
DAPs	Differentially abundant proteins
ER	Endoplasmic reticulum
GSEA	Gene set enrichment analysis
iPSC	Induced pluripotent stem cell
mDA	Midbrain dopaminergic neuron
mTOR	Mechanistic target of rapamycin
NEFL	Neurofilament light chain
NEFH	Neurofilament heavy chain
PRPH	Peripherin
SYNM	Synemin
PSP-RS	Progressive Supranuclear Palsy-Richardson’s Syndrome
