# Supplementary material for: Cytoskeletal Imbalance and Axonal Vulnerability in Sporadic PSP-RS: Early Changes in a Human iPSC-Derived Neuronal Model with Altered mTOR Signaling
Source: Cells. 2026 Apr 23;15(9):754. doi: 10.3390/cells15090754 (PMC13162785; doi:10.3390/cells15090754)
Supplement: Supplementary file 1 [file cells-15-00754-s001.zip › Supplementary Figures and Legends.pdf]

Cytoskeletal imbalance and axonal vulnerability in sporadic PSP-RS: early changes in a human iPSC-derived neuronal model with altered mTOR signaling

Raffaele Covello[1,5], Giorgia Lucia Benedetto[2,5], Stefania Scalise[1,*], Caterina Gabriele[1], Desirèe Valente[1], Clara Zannino[3], Barbara Puccio[2], Andrea Quattrone[2,4], Pietro Hiram Guzzi[2], Marco Gaspari[1], Aldo Quattrone[4], Giovanni Cuda[1,*], Elvira Immacolata Parrotta[2]

[1] Department of Experimental and Clinical Medicine, University Magna Graecia of Catanzaro, Catanzaro, Italy.

[2] Department of Medical and Surgical Sciences, University Magna Graecia of Catanzaro, Catanzaro, Italy.

[3] Department of Experimental Medical Science, Lund University, Lund, Sweden.

[4] Neuroscience Research Center, University Magna Graecia of Catanzaro, Catanzaro, Italy.

[5] These authors contributed equally to this work

[*] Correspondance: Stefania Scalise, stefania.scalise@unicz.it; Giovanni Cuda, cuda@unicz.it

Supplementary Figures and Legends

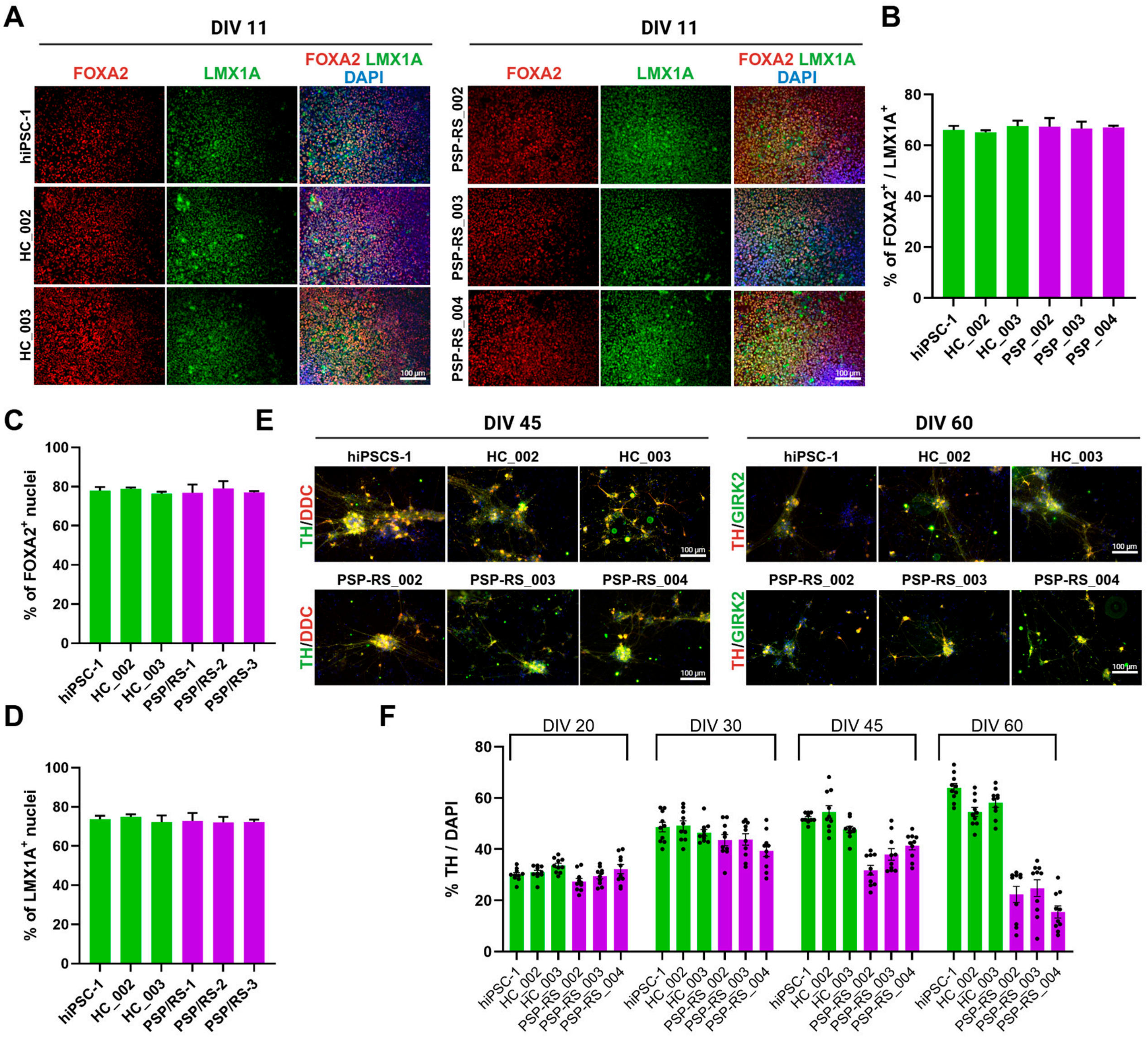

Supplementary Figure S1. Stage-specific expression of midbrain dopaminergic markers in individual HC and PSP-RS lines. A, Representative immunofluorescence images showing expression of FOXA2 (red) and LMX1A (green) in midbrain floor-plate progenitors (mFPPs) at DIV11 in the individual cell lines. Nuclei were counterstained with DAPI (blue). Scale bar, 100 µm. **B**, Quantification of FOXA2 and LMX1A double-positive cells. **C, D**, Quantification of FOXA2 and LMX1A positive nuclei. For (B), (C), and (D), at least 1,200 nuclei were counted per condition. **E,** Representative immunofluorescence images showing expression of TH (green) and DDC (red) at DIV45 and TH (red) and GIRK2 (green) at DIV60 in individual HC and PSP-RS lines. Nuclei were counterstained with DAPI (blue). Scale bar, 100 µm. **F,** Quantification of TH^{+} nuclei (counterstained with DAPI) during differentiation (DIV 20–60) in HC and PSP-RS neurons. Data are presented as mean ± s.e.m.; $N = 30$ images per group derived from two independent differentiation experiments; a minimum of 1000 nuclei were analyzed per condition.

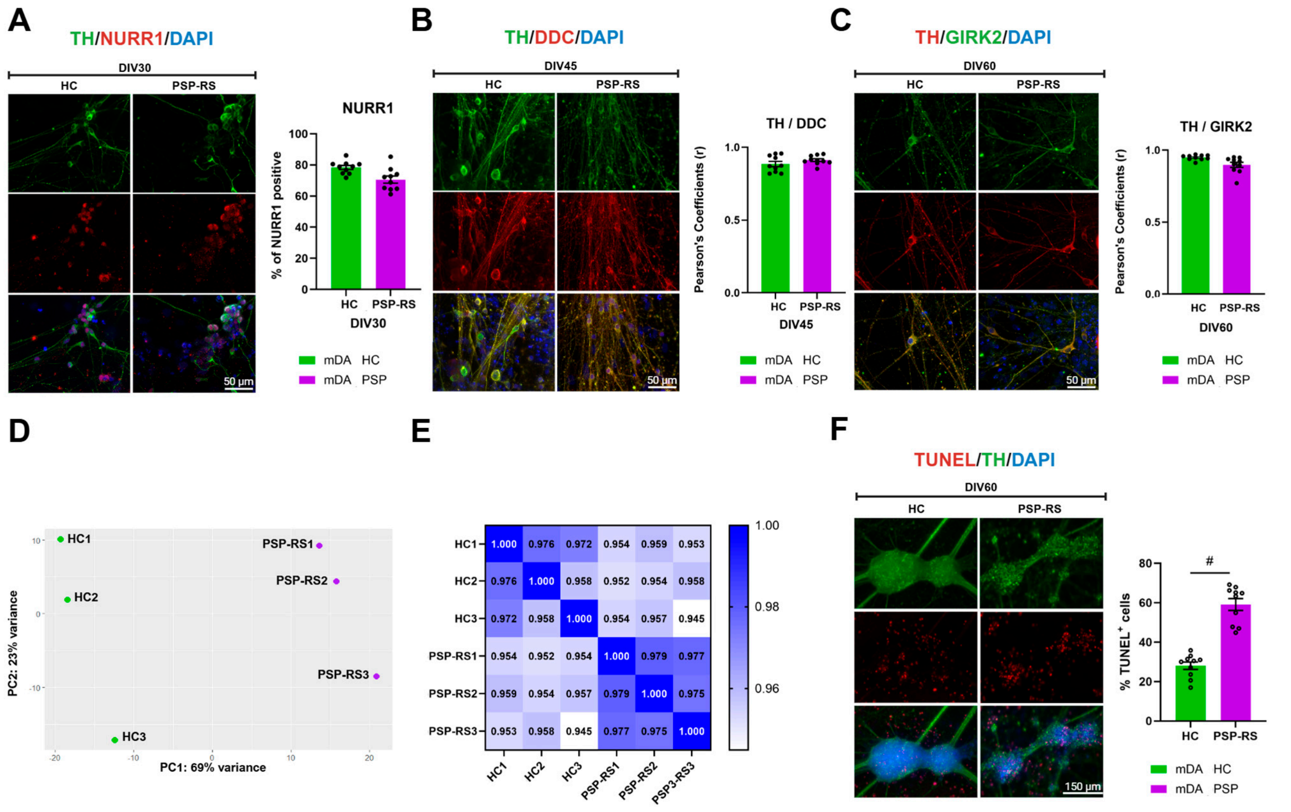

Supplementary Figure S2. Differentiation of pooled PSP-RS and control mFPPs into midbrain dopaminergic neurons. A, B, C Representative immunofluorescence images and quantification showing stage-specific expression of NURR1 (DIV 30), DDC (DIV 45), and GIRK2 (DIV 60) in pooled mDA neuron cultures derived from HC and PSP-RS patients. Scale bar, 50 µm. For quantification of NURR1⁺ neurons, data are presented as mean ± s.e.m.; *N* = 10 images per group derived from three independent differentiation experiments. For colocalization analysis of TH⁺/DDC⁺ at DIV 45 and TH⁺/GIRK2⁺ at DIV 60 in pooled mDA neurons, Pearson's correlation coefficient was quantified. Data are mean ± s.e.m.; *N* = 10 images per group derived from three independent differentiation experiments. **D, E** Principal component analysis (PCA) and Pearson correlation matrix of RNA-seq data from HC and PSP-RS mDA neurons at DIV 60. **F** Representative immunofluorescence images and quantification of TUNEL-positive cells (red) in mDA PSP-RS and HC neurons at DIV60. Neurons were marked by Tyrosine hydroxylase (TH) staining in green. Magnification: 63X. Each dot represents a single image analyzed; data are represented as mean ± s.e.m., #p ≤ 0.0001 (unpaired t-test with Welch's correction).

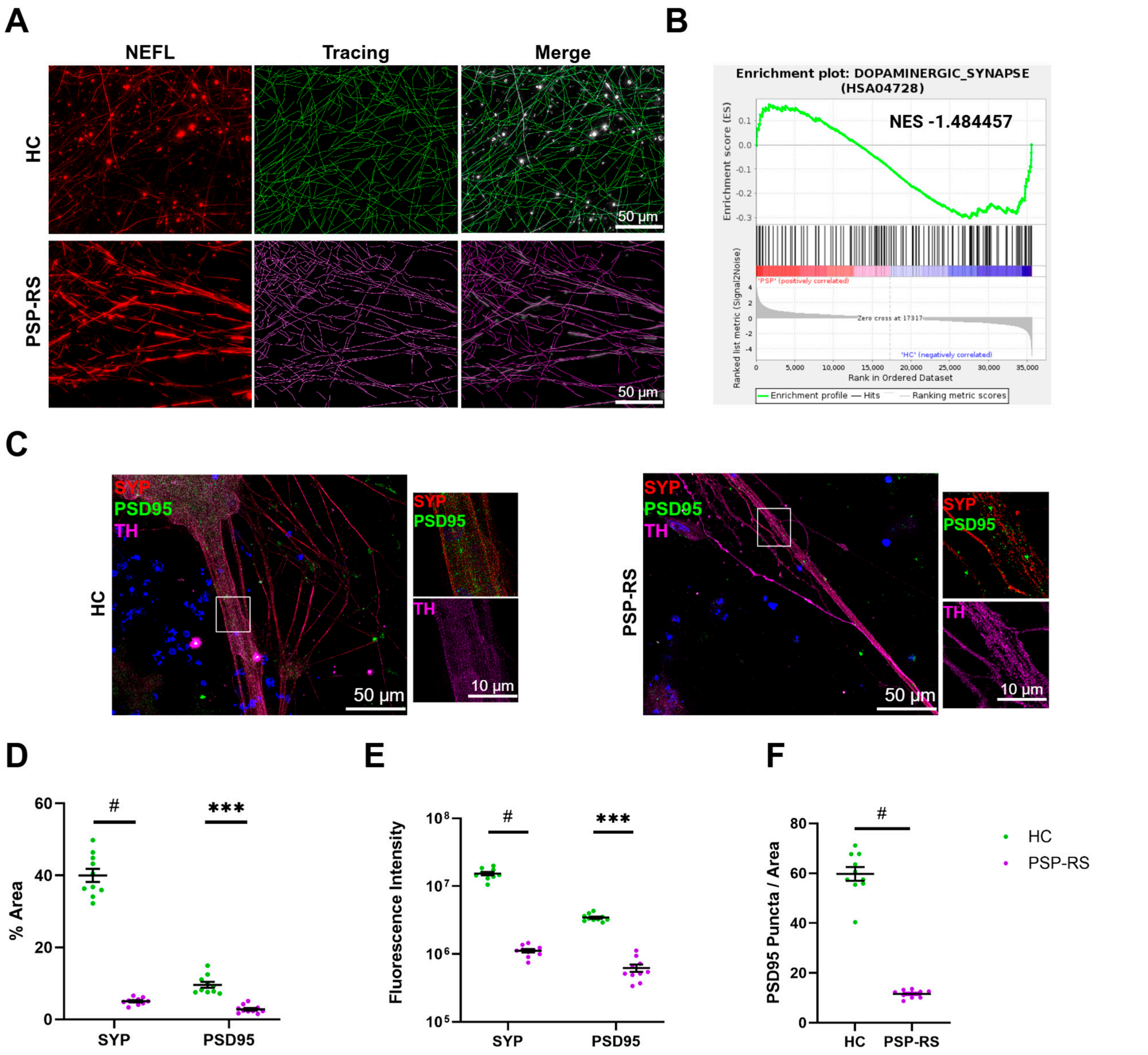

Supplementary Figure S3. Reduction of axonal length and synaptic markers in PSP-RS midbrain dopaminergic neurons. A, Representative immunofluorescence images of NEFL^{+} neurons from HC and PSP-RS cultures at DIV 60, with neuronal length traced using the NeuronJ plugin (ImageJ). Scale bar, 50 µm. **B**, GSEA showing negative enrichment of proteins associated with the dopaminergic synapse in PSP-RS mDA neurons. **C**, Representative immunofluorescence images of tyrosine hydroxylase TH (magenta), and synaptic markers SYP (red) and PSD95 (green) in HC and PSP-RS mDA neurons at DIV 60. Nuclei were counterstained with DAPI (blue). Scale bars, 50 µm (overview) and 10 µm (insets). **D-F**, Quantification of percentage area coverage (D) and fluorescence intensity (E) of SYP and PSD95 signals, and PSD95 puncta density (puncta per unit area, a.u.) (F), in HC and PSP-RS dopaminergic neurons at DIV 60. Data are mean ± s.e.m.; $N = 10$ images per group derived from three independent differentiation experiments; ***$p < 0.001$, $^{\#}p < 0.0001$ (unpaired *t*-test with Welch's correction).

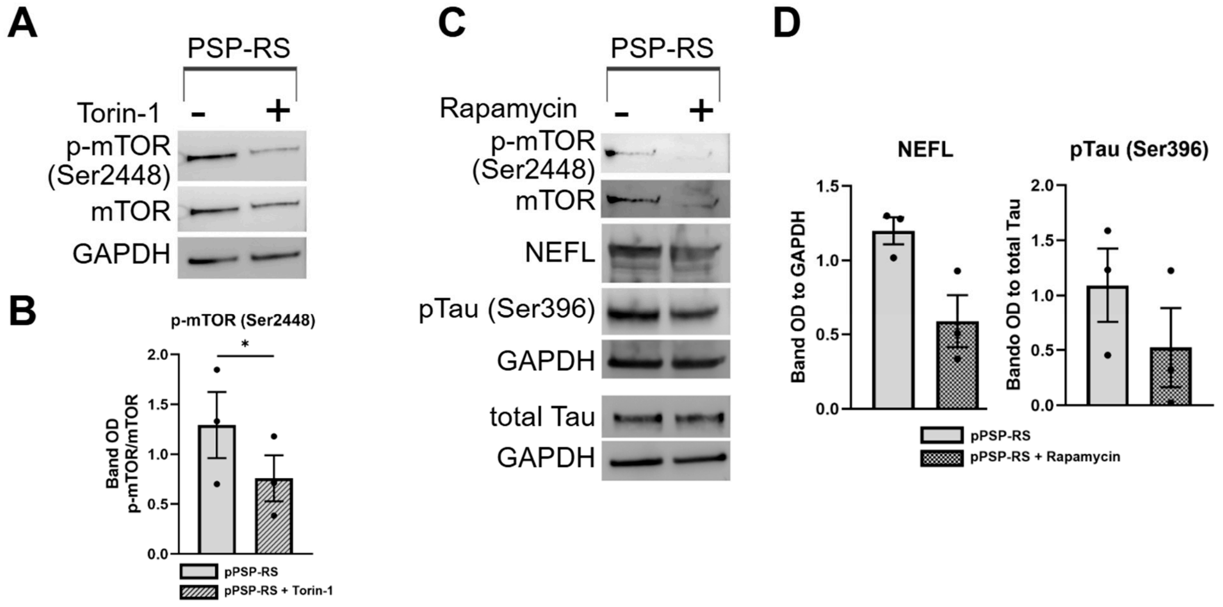

Supplementary Figure S4. Torin-1 and rapamycin treatments in PSP-RS mDA neurons. A, B, Representative Western blot (A) and densitometric quantification (B) of phosphorylated mTOR (p-mTOR Ser2448) normalized to total mTOR in PSP-RS mDA neurons at DIV 60, following 24 h treatment with Torin-1 (100 nM). **C**, **D**, Representative Western blot (A) and densitometric quantification (B) of NEFL normalized to GAPDH, and phosphorylated Tau (p-Tau Ser396) normalized to total Tau, in PSP-RS mDA neurons at DIV 60, following 24 h treatment with Rapamycin (1 μM). For (B) and (D), data are presented as mean ± s.e.m. of three independent differentiation experiments; *$p \leq 0.05$, (unpaired t-test with Welch's correction).
