# Supplementary material for: Cytoskeletal Imbalance and Axonal Vulnerability in Sporadic PSP-RS: Early Changes in a Human iPSC-Derived Neuronal Model with Altered mTOR Signaling
Source: Cells. 2026 Apr 23;15(9):754. doi: 10.3390/cells15090754 (PMC13162785; doi:10.3390/cells15090754)
Supplement: Supplementary file 1 [file cells-15-00754-s001.zip › Supplementary File S1_pdf.pdf]

Figure 1G - TH

Marker: Precision Plus Protein #161-0374

TH - 60kDa

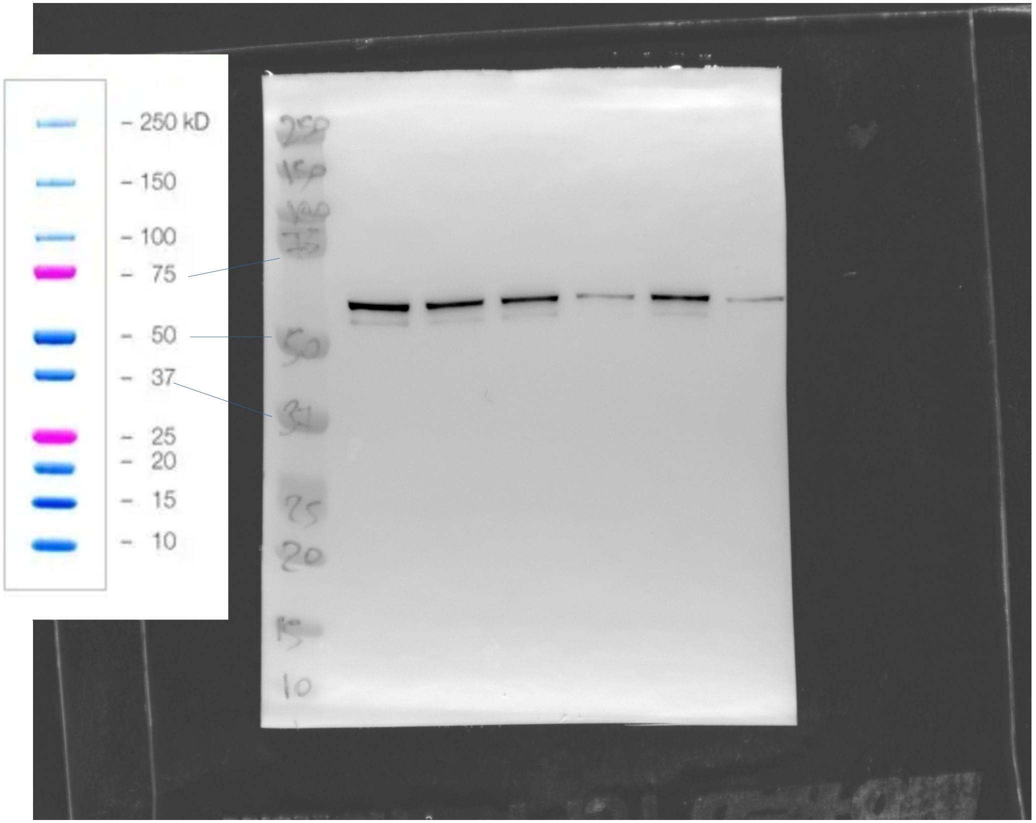

GAPDH - 37kDa

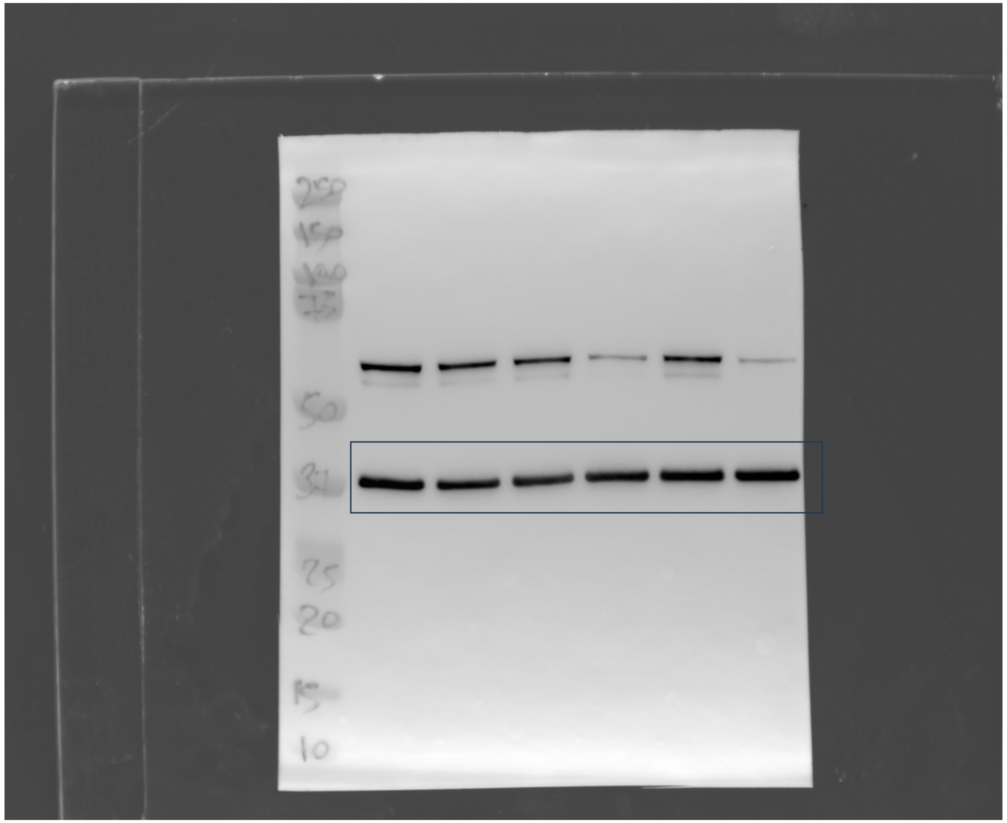

Figure 1G - DDC

Marker: Precision Plus Protein #161-0374

DDC - 54kDa

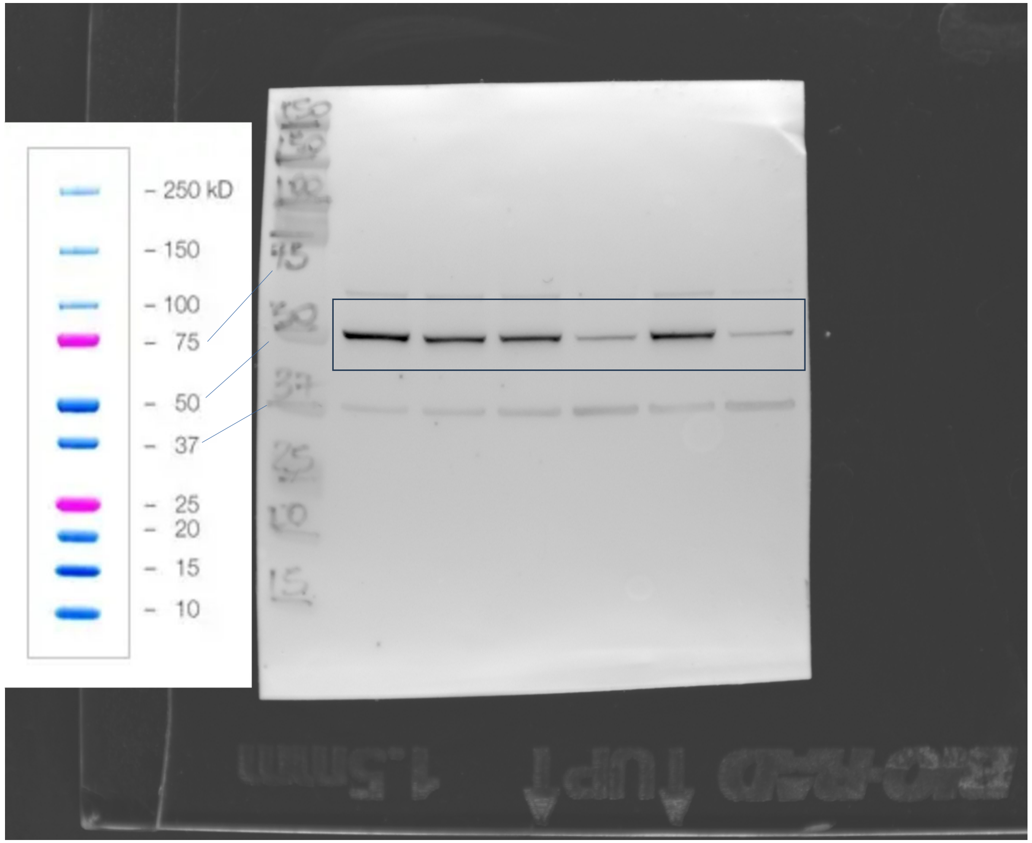

GAPDH - 37kDa

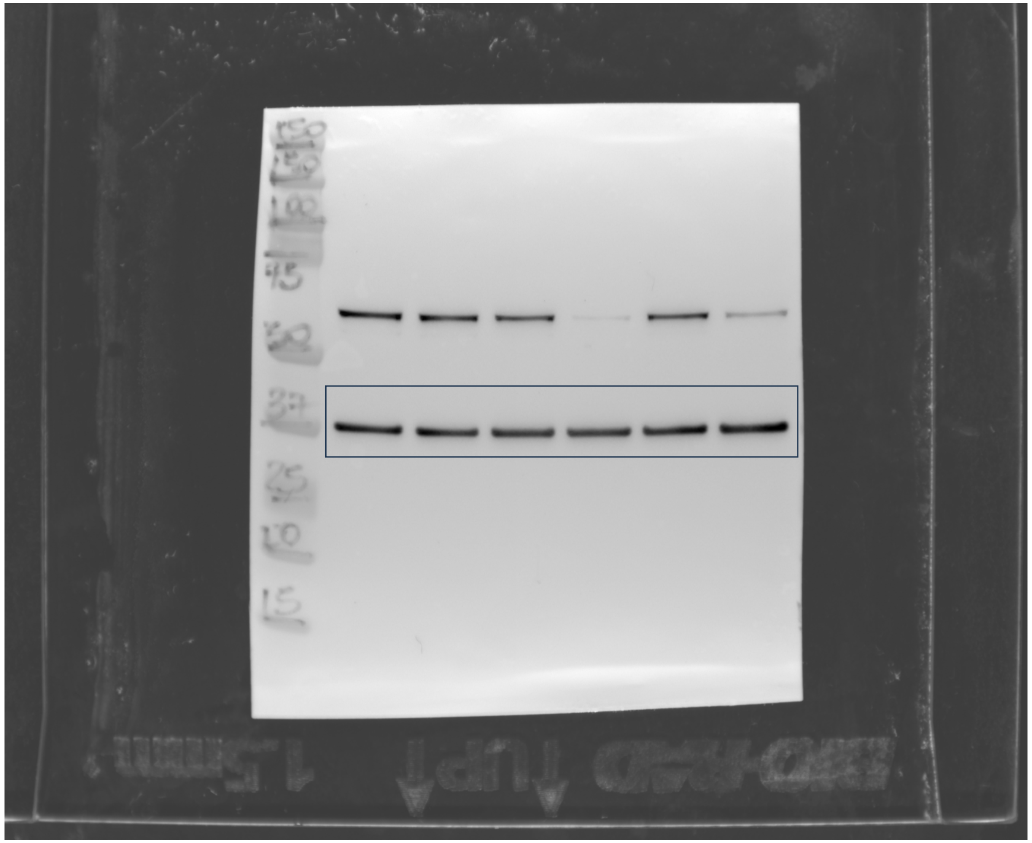

Figure 1I - MAOB
Marker: Precision Plus Protein #161-0374

MAOB - 62kDa

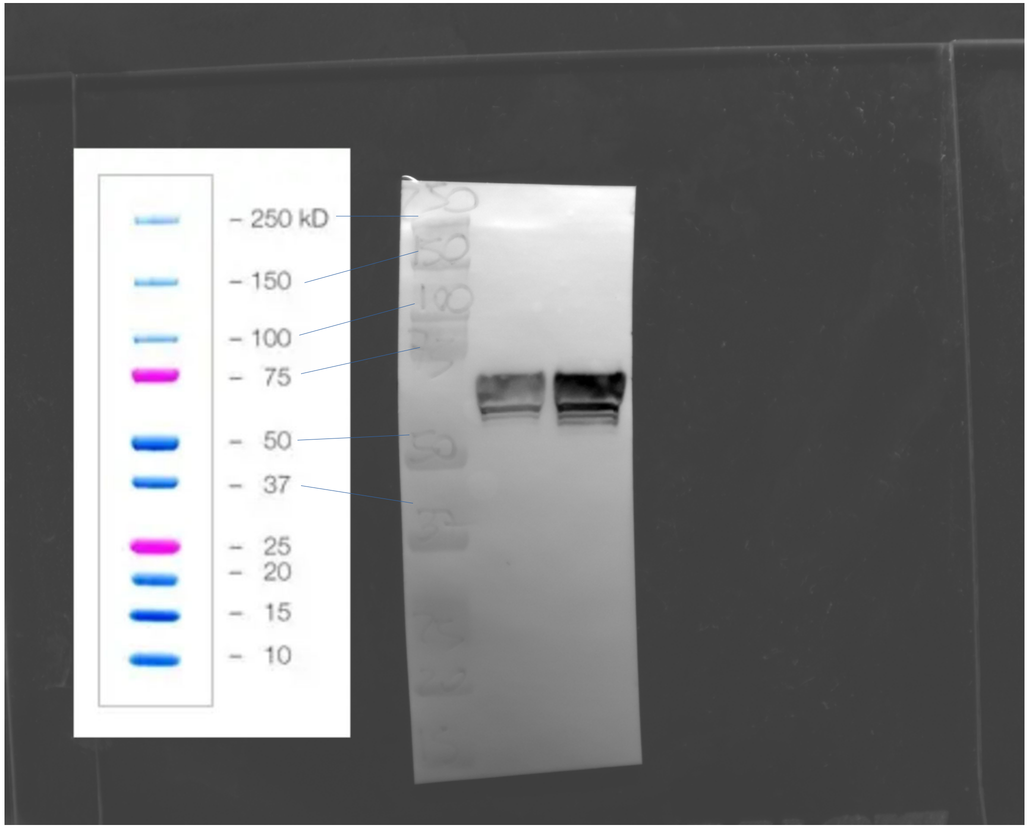

GAPDH - 37kDa

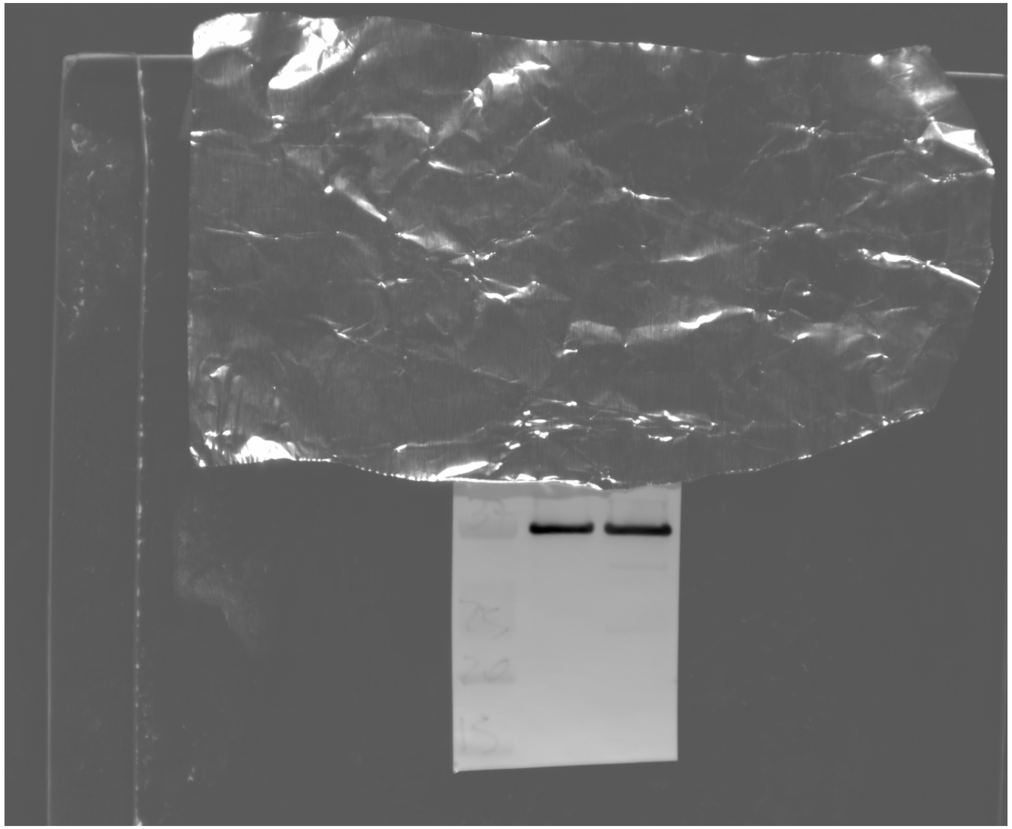

Figure 3F – pTau (Ser396)

Marker: SeeBlue Plus2 #LC5925

pTau(Ser396) - 50kDa

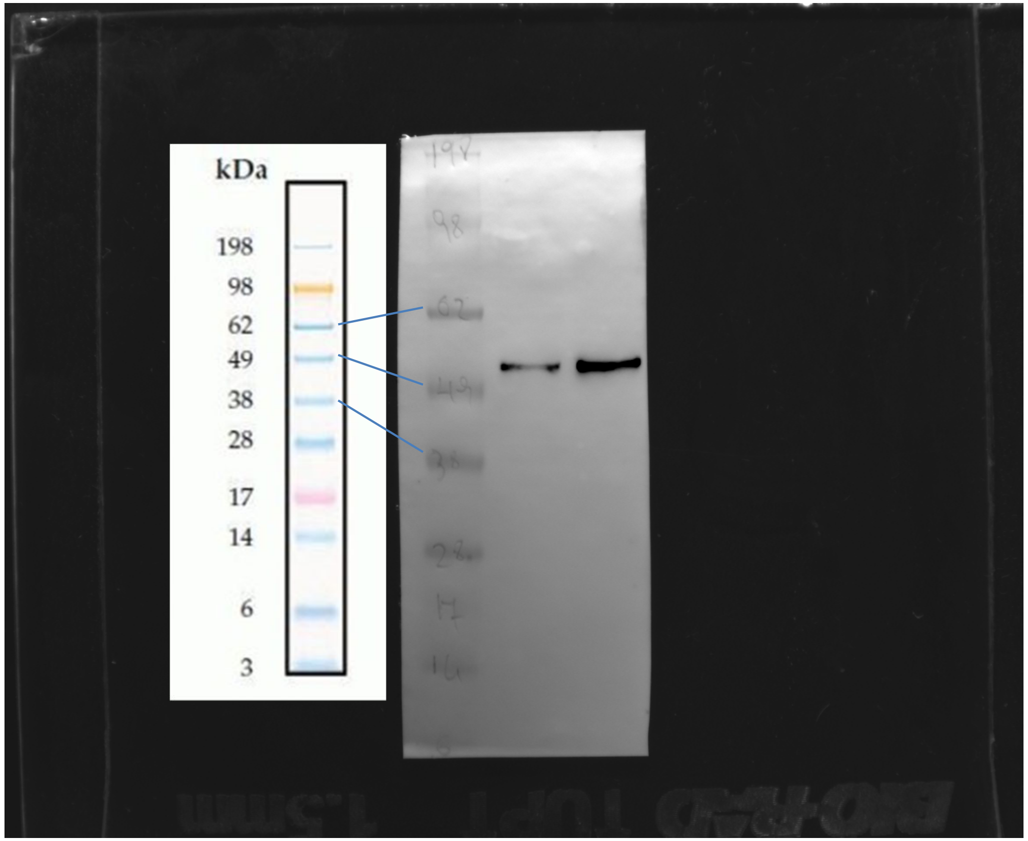

GAPDH - 37 kDa

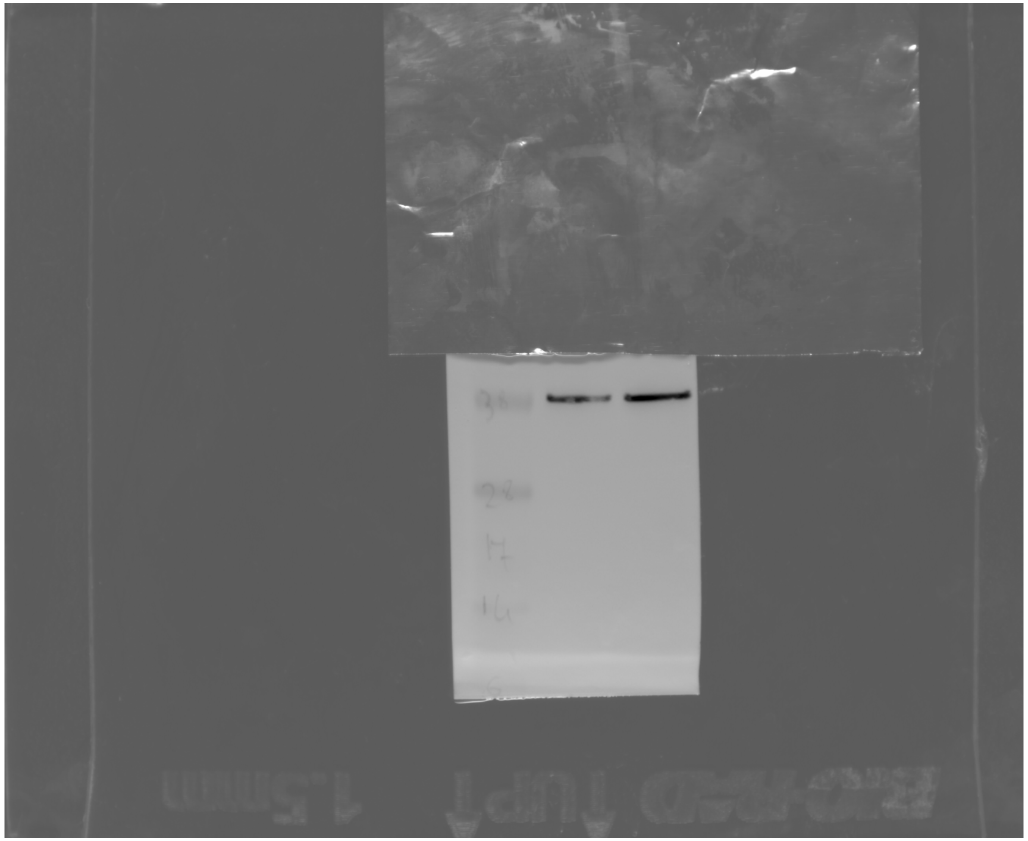

Figure 3F – pTau (AT8)

Marker: SeeBlue Plus2 #LC5925

pTau(AT8) - 50kDa

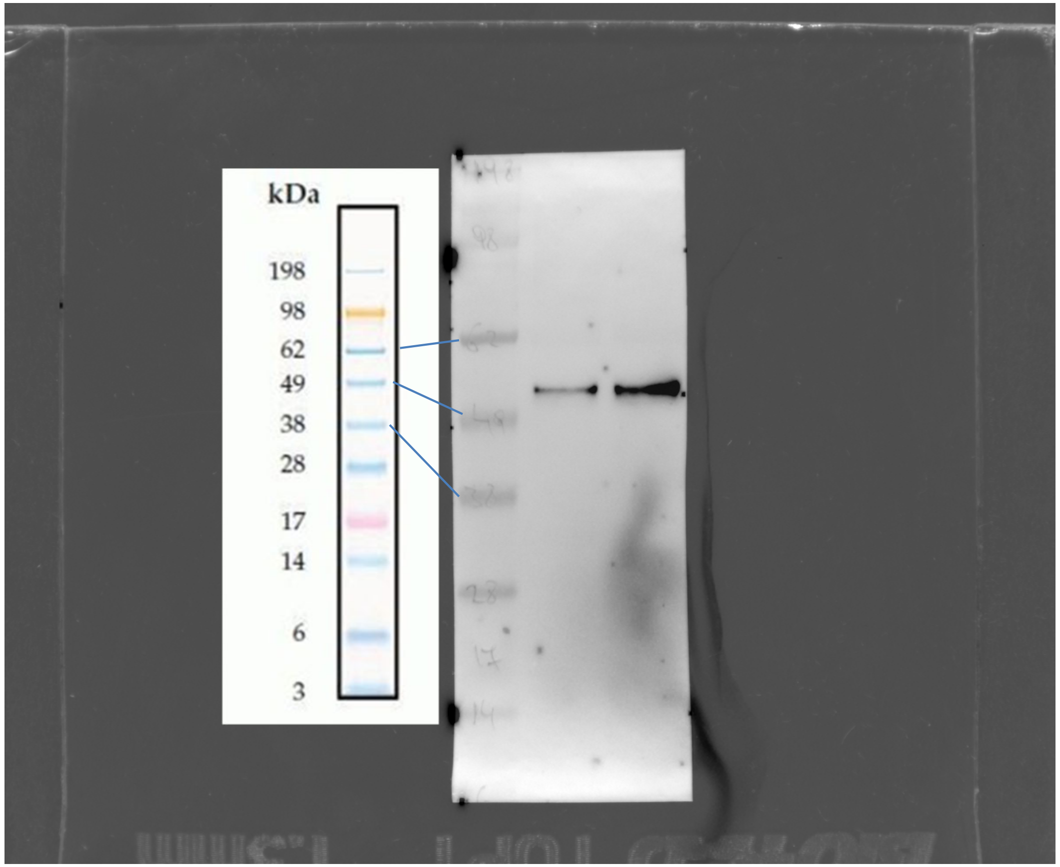

GAPDH - 37 kDa

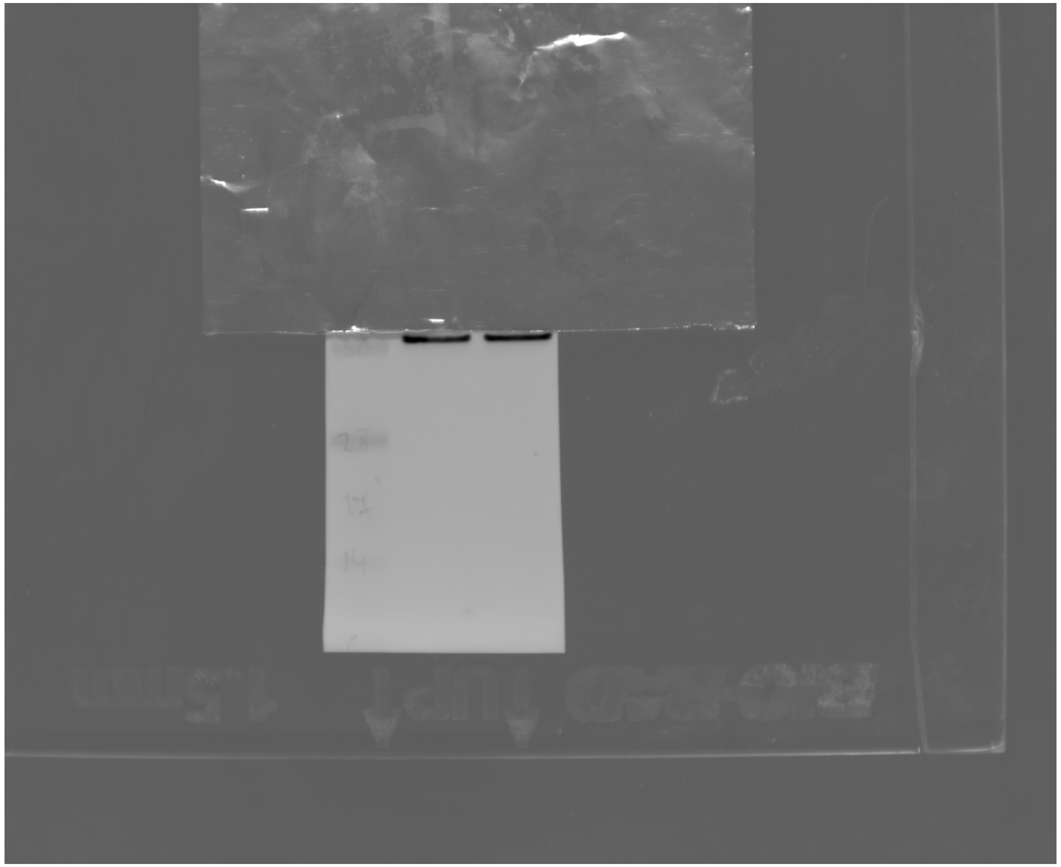

Figure 3F – total Tau

Marker: SeeBlue Plus2 #LC5925

Total Tau- 50kDa

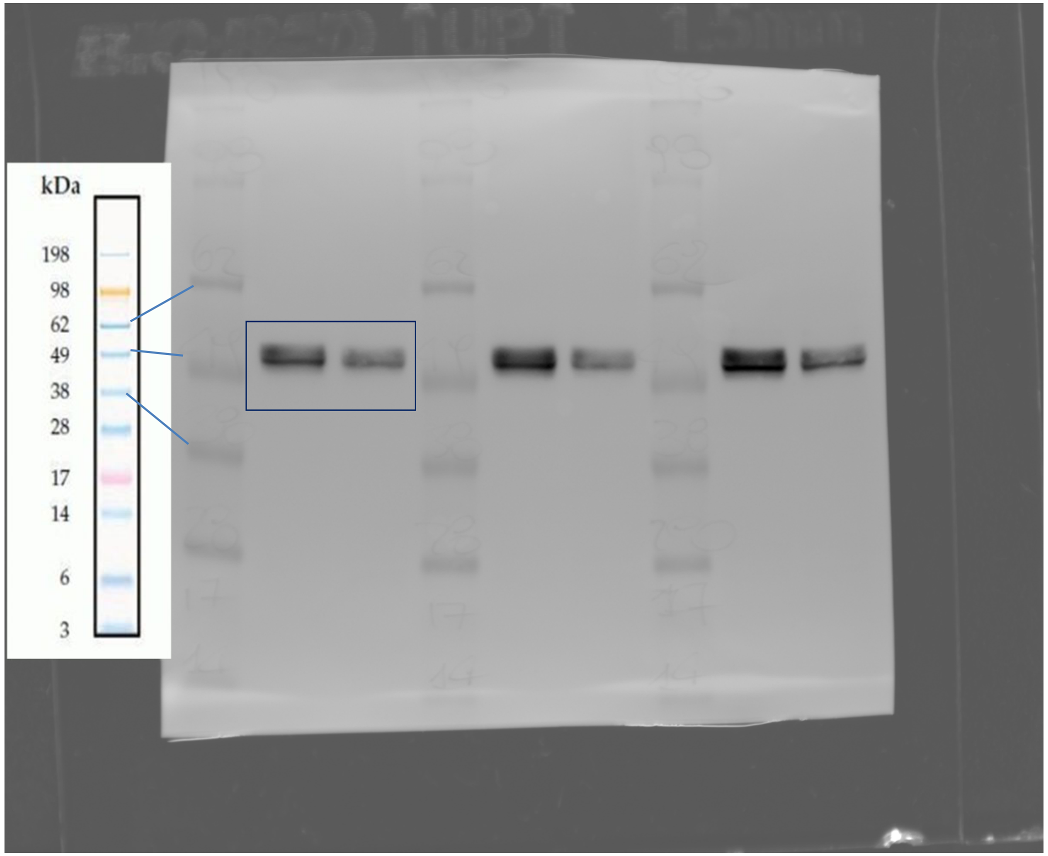

GAPDH - 37kDa

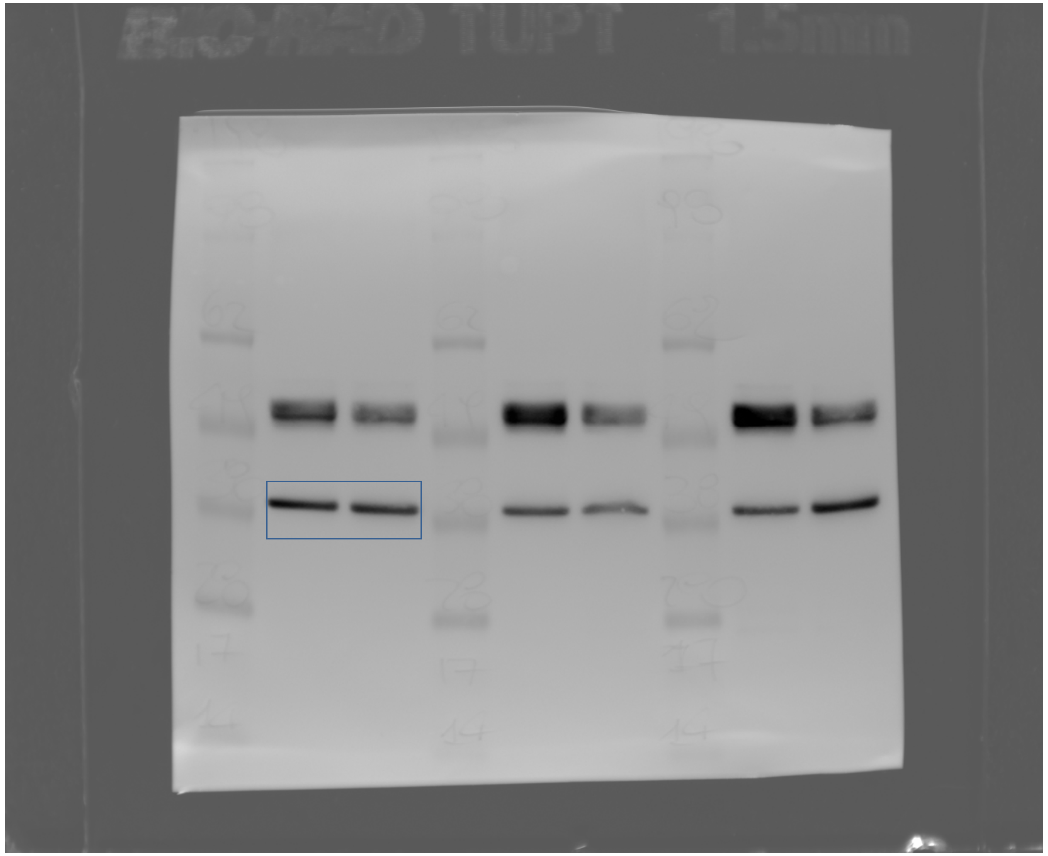

Figure 4D - NEFL

Marker: Precision Plus Protein #161-0374

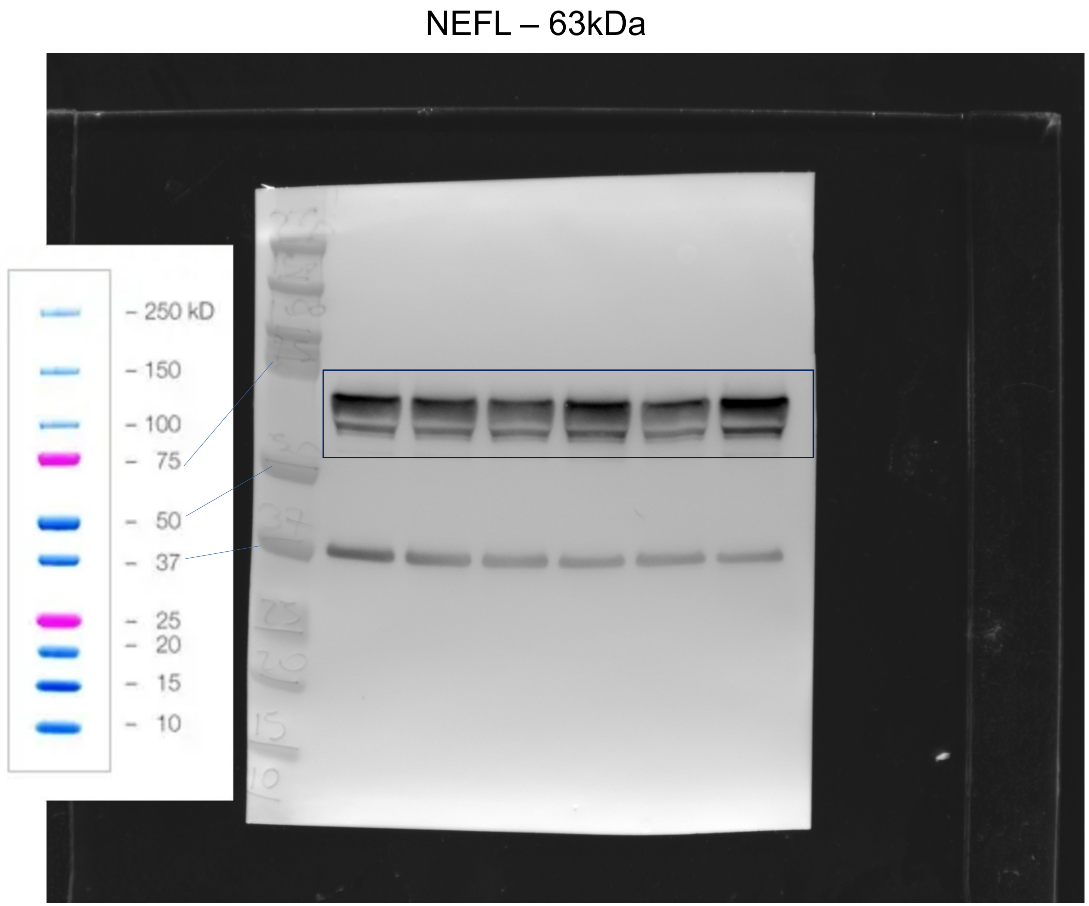

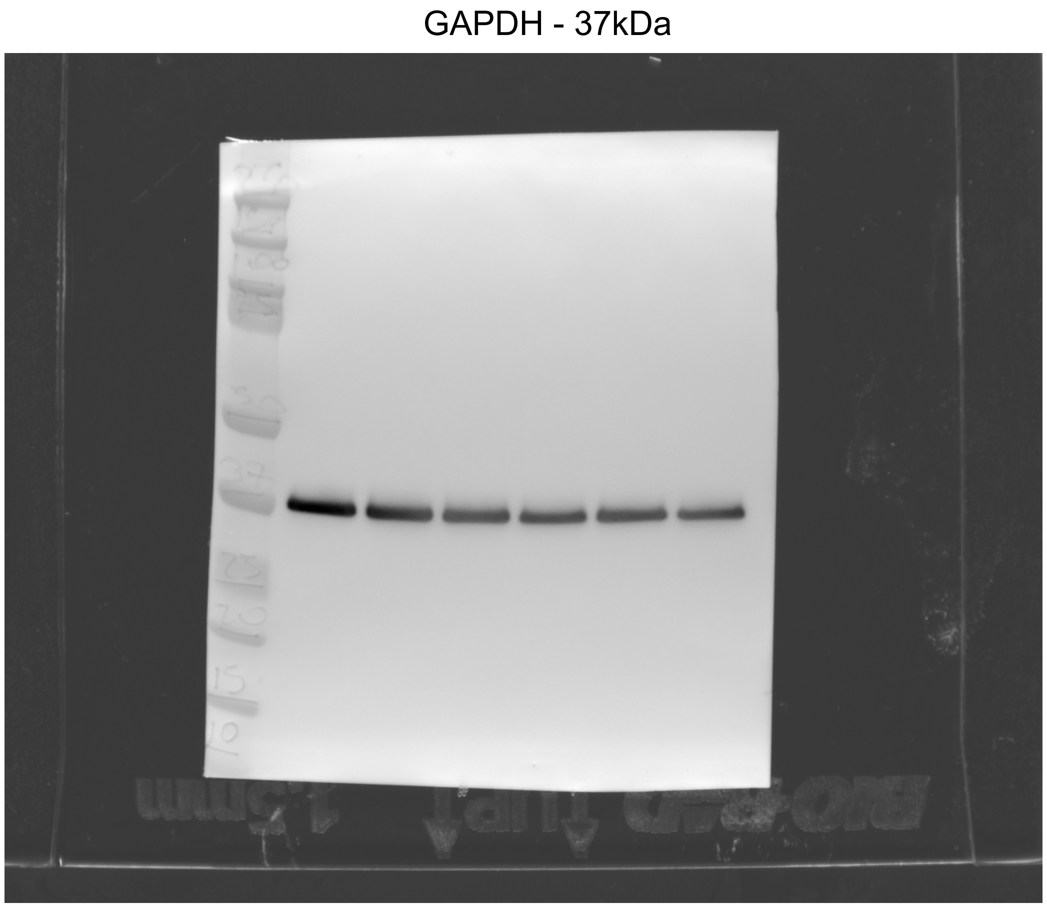

Figure 4F - NEFH
Marker: Himark pre-stained #LC5699

NEFH - 200kDa

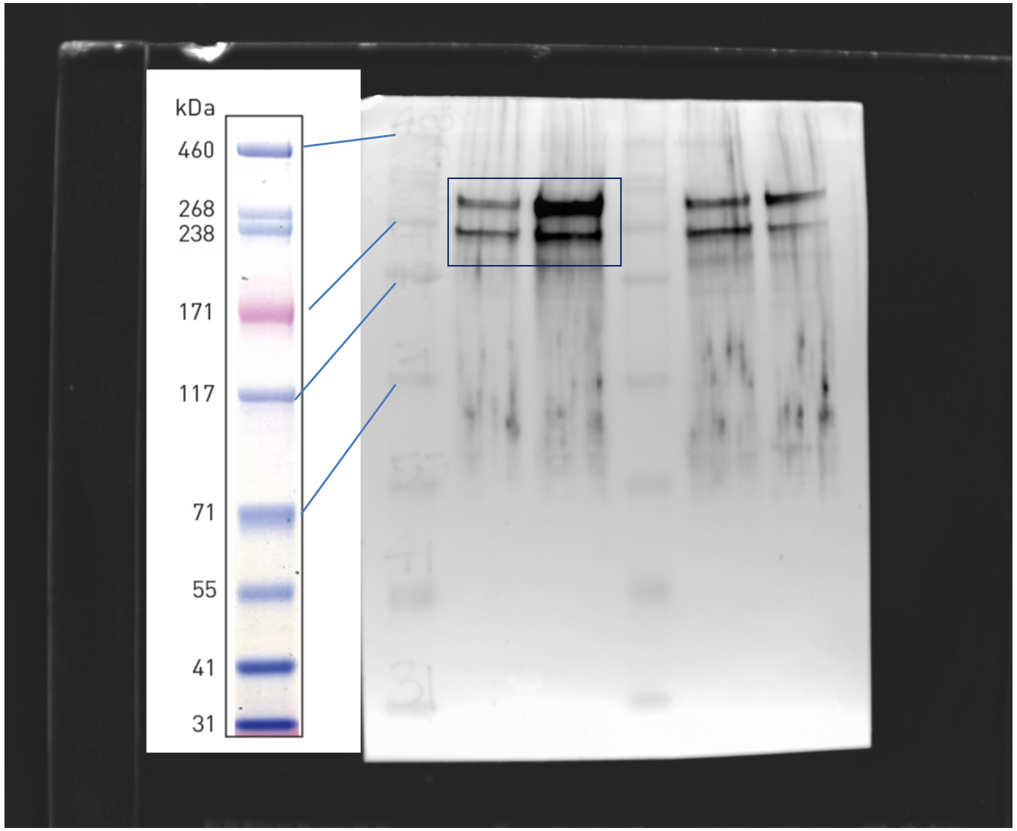

GAPDH - 37kDa

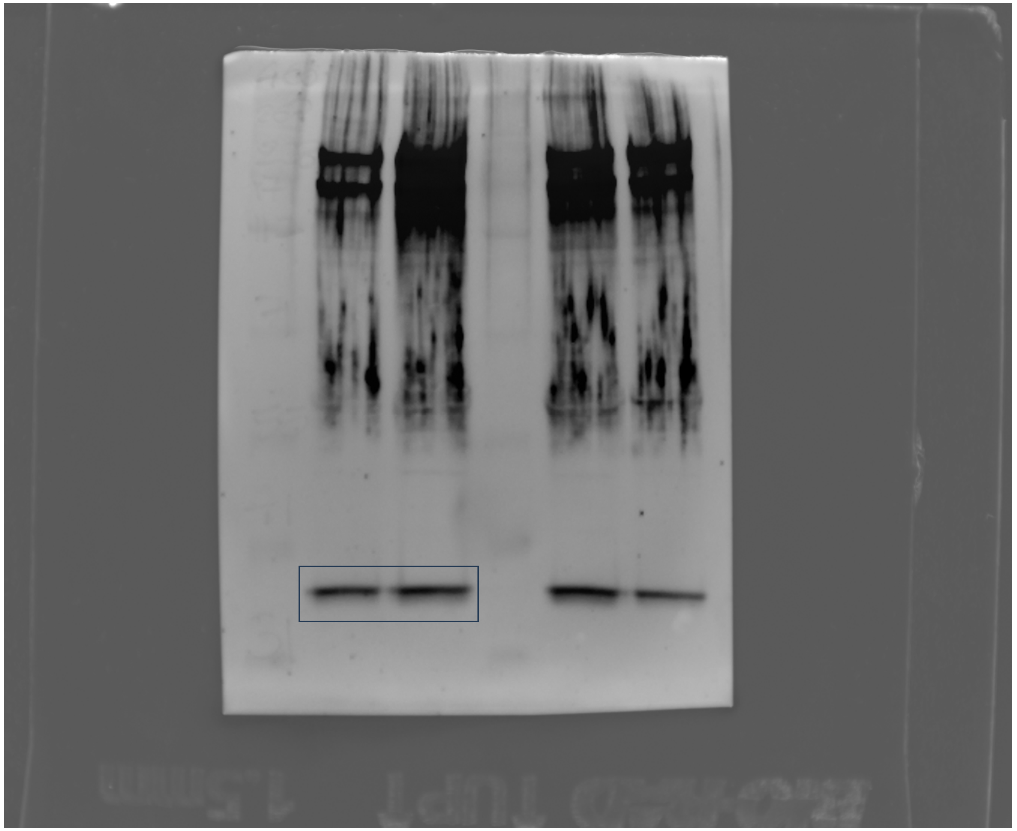

Figure 4M - SYP

Marker: Precision Plus Protein #161-0374

SYP – 38kDa

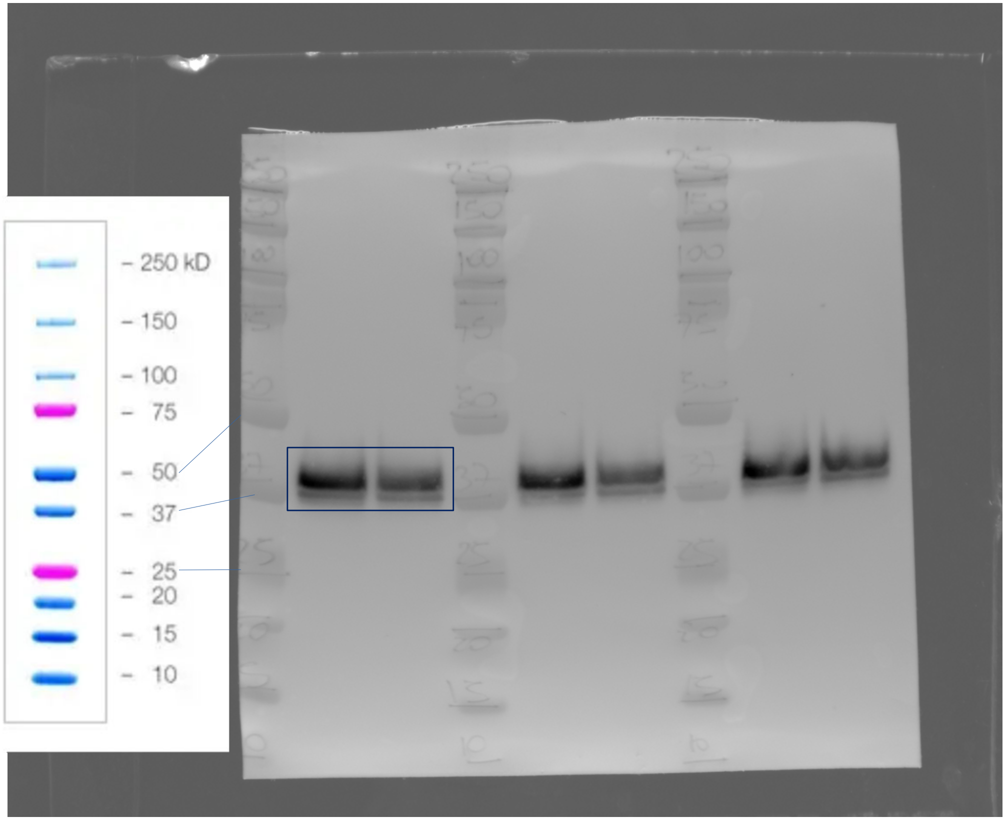

GAPDH - 37kDa

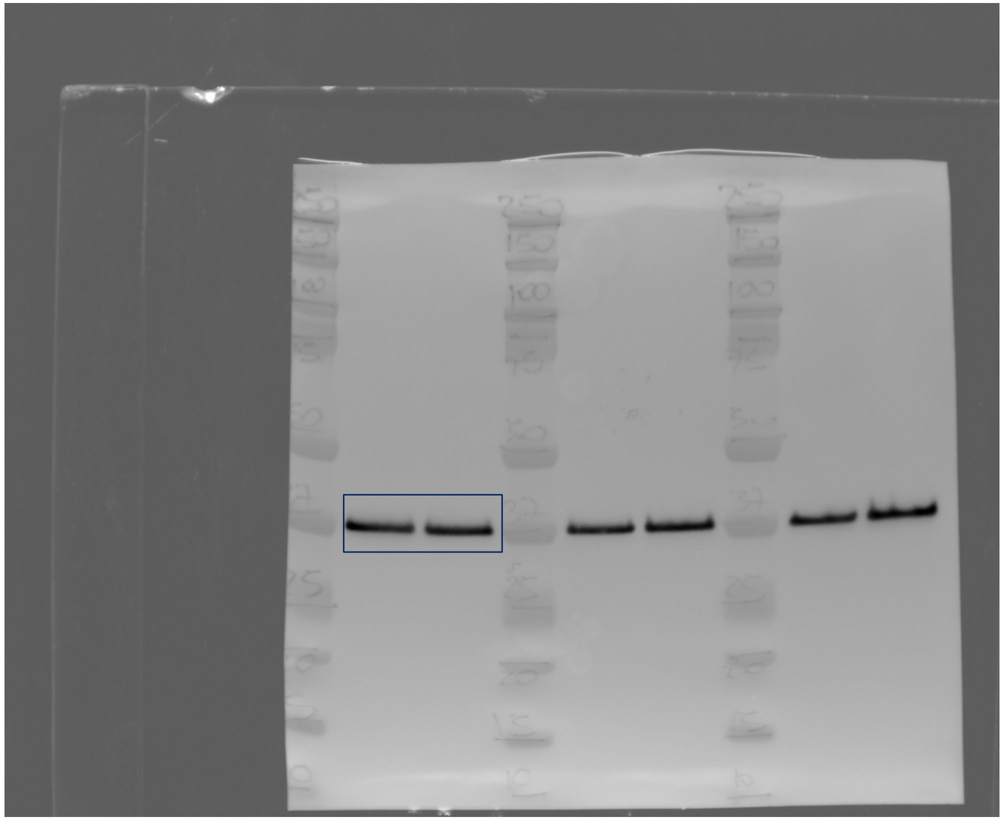

Figure 4M – PSD95

Marker: Precision Plus Protein #161-0374

PSD95 ~ 80kDa

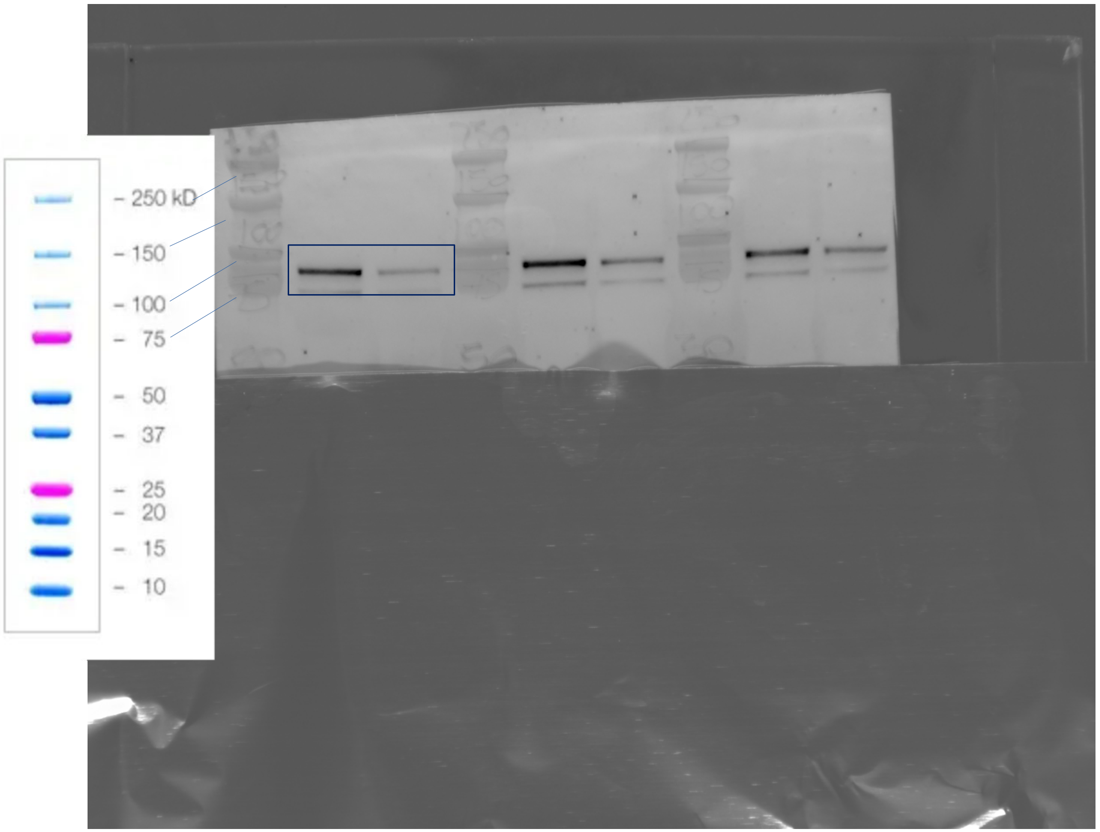

GAPDH - 37kDa

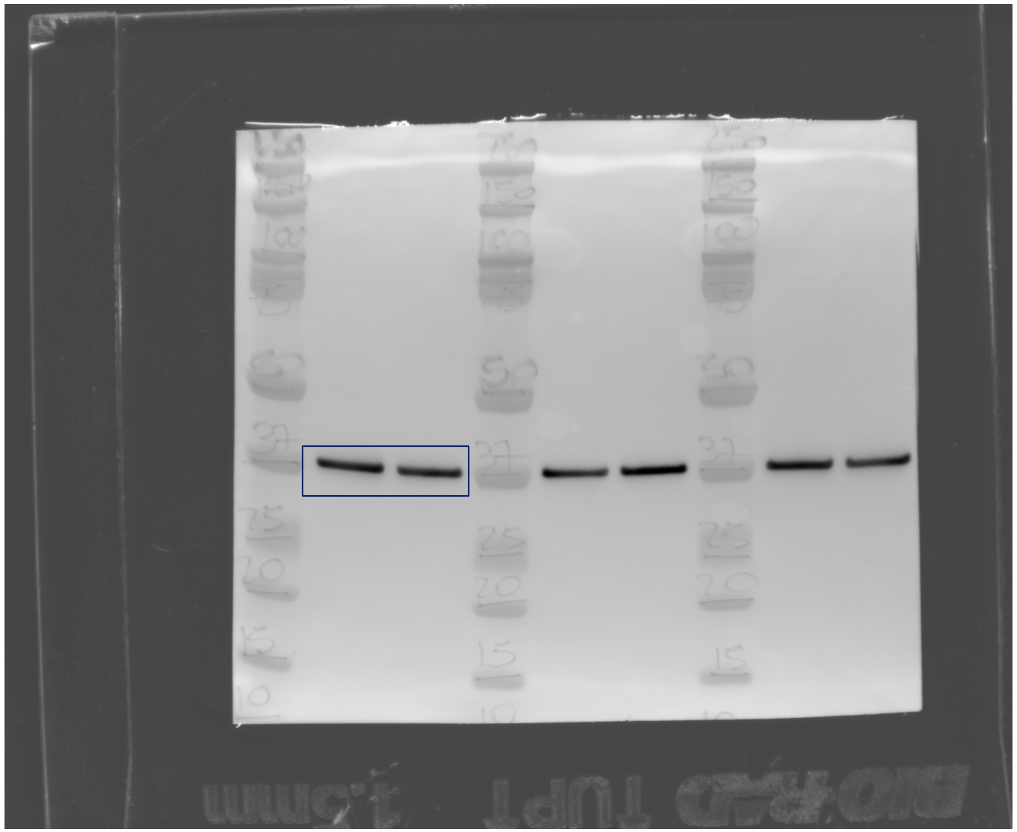

Figure 4N – VAMP2-STX1A

Marker: SeeBlue Plus2 #LC5925

VAMP2 – 17kDa

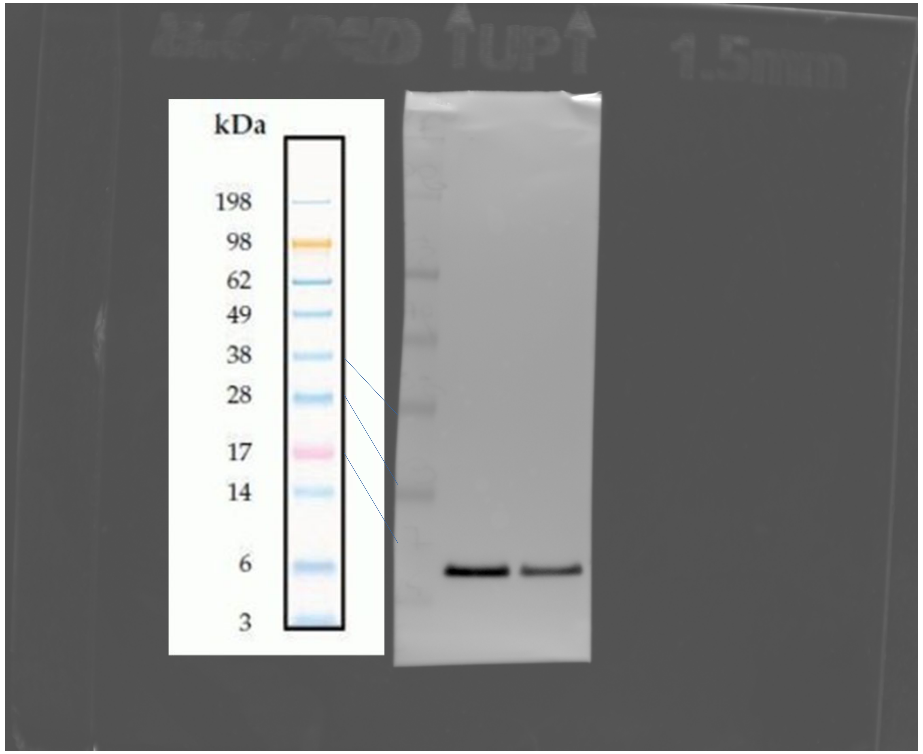

STX1A – 33kDa

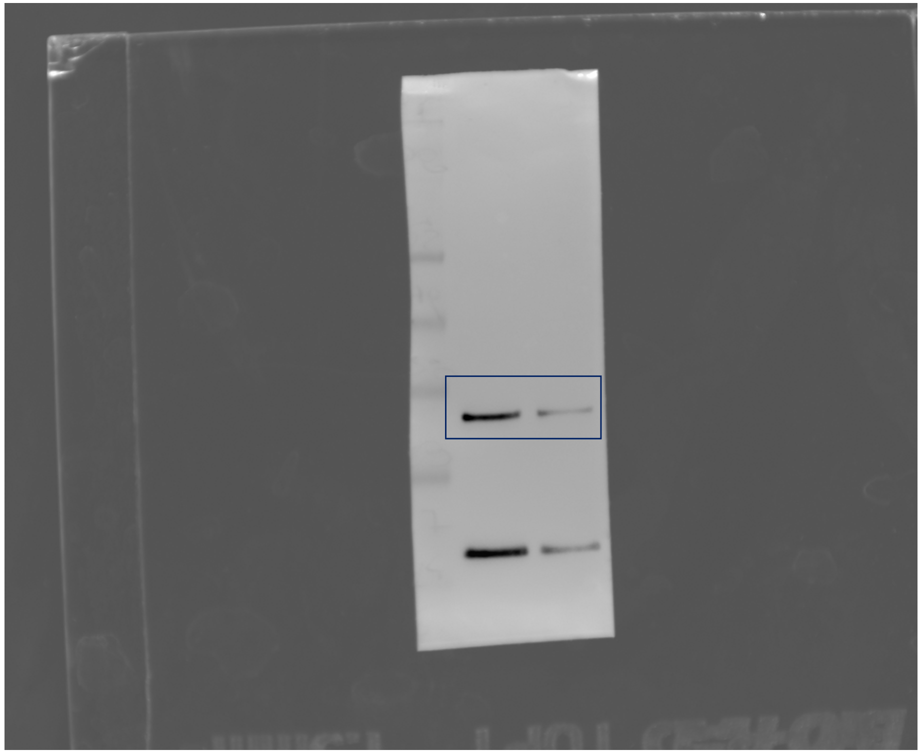

For figure 4N, VAMP2 and STX1A were detected on the same membrane; GAPDH is shared among targets.

Figure 4N – VAMP2-STX1A

Marker: SeeBlue Plus2 #LC5925

GAPDH – 37kDa

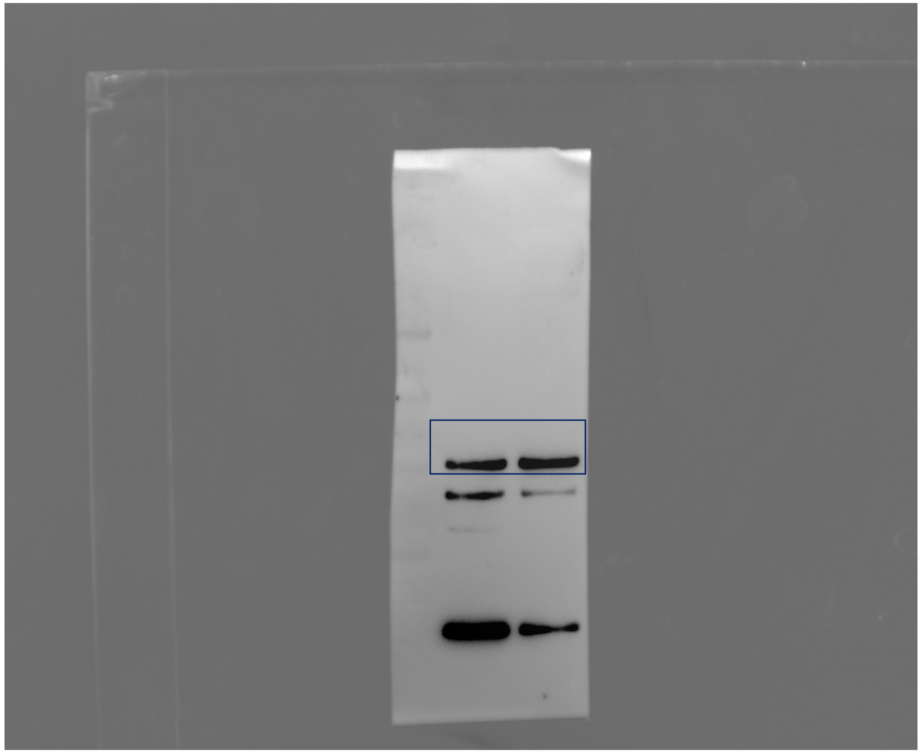

For figure 4N, VAMP2 and STX1A were detected on the same membrane; GAPDH is shared among targets.

Figure 5A – pmTOR - mTOR

Marker: Precision Plus Protein #161-0374

phospho-mTOR (Ser2448) – 250kDa

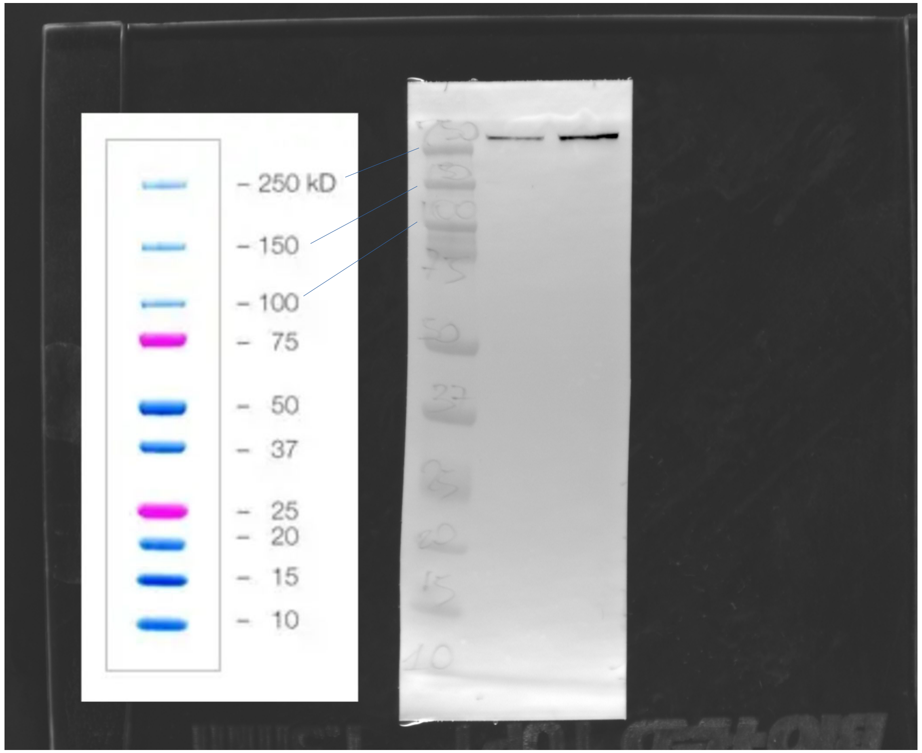

mTOR – 250kDa

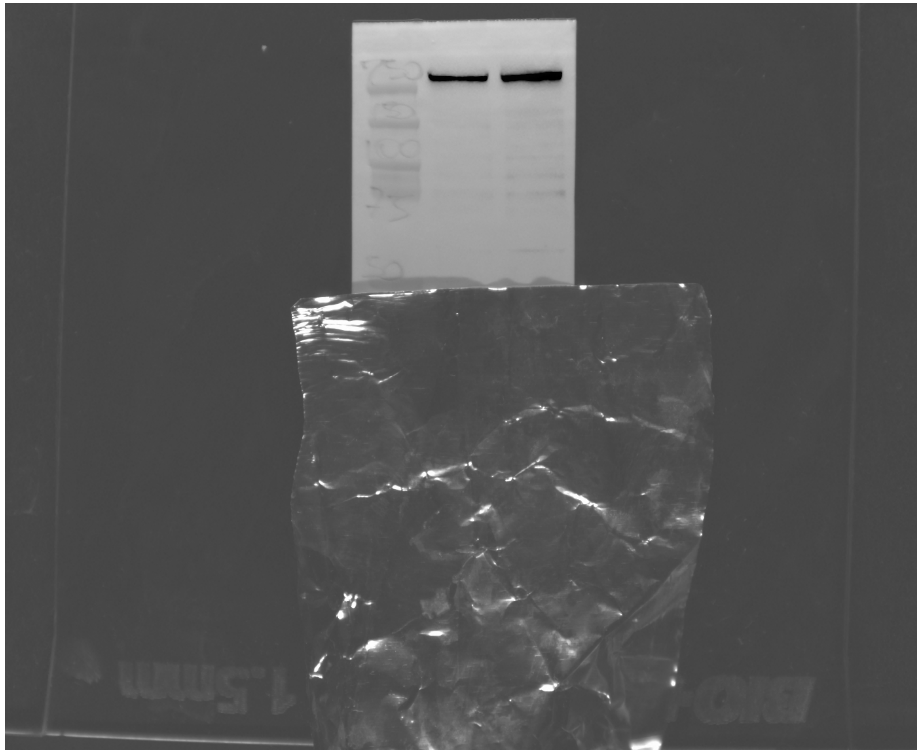

Figure 5A –mTOR - p-mTOR

Marker: Precision Plus Protein #161-0374

GAPDH - 37kDa

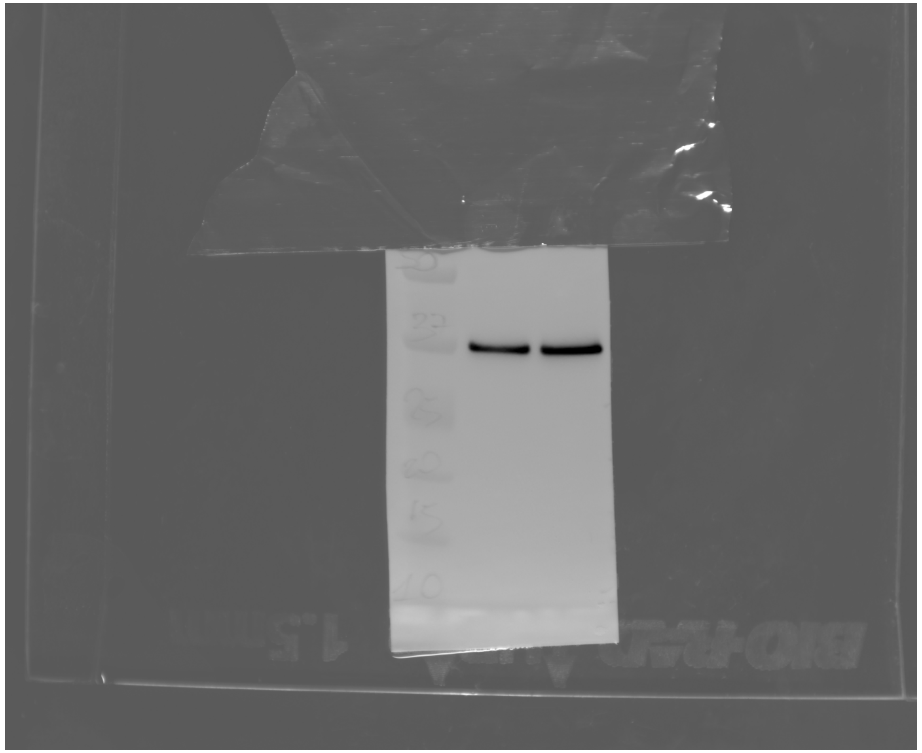

Figure 5C – NEFL

Marker: Precision Plus Protein #161-0374

NEFL – 63kDa

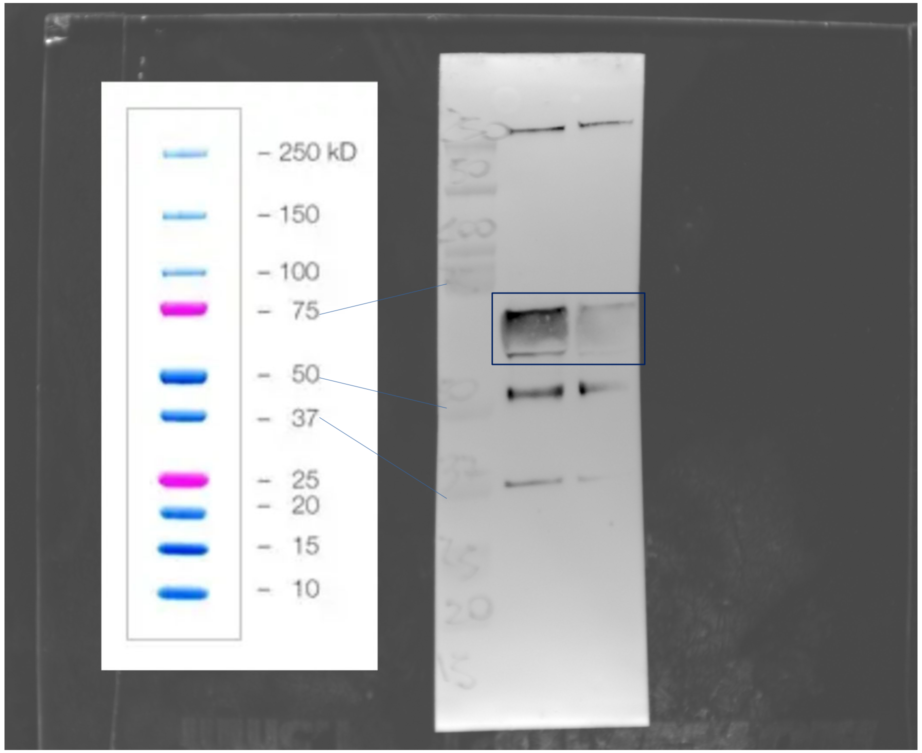

GAPDH - 37kDa

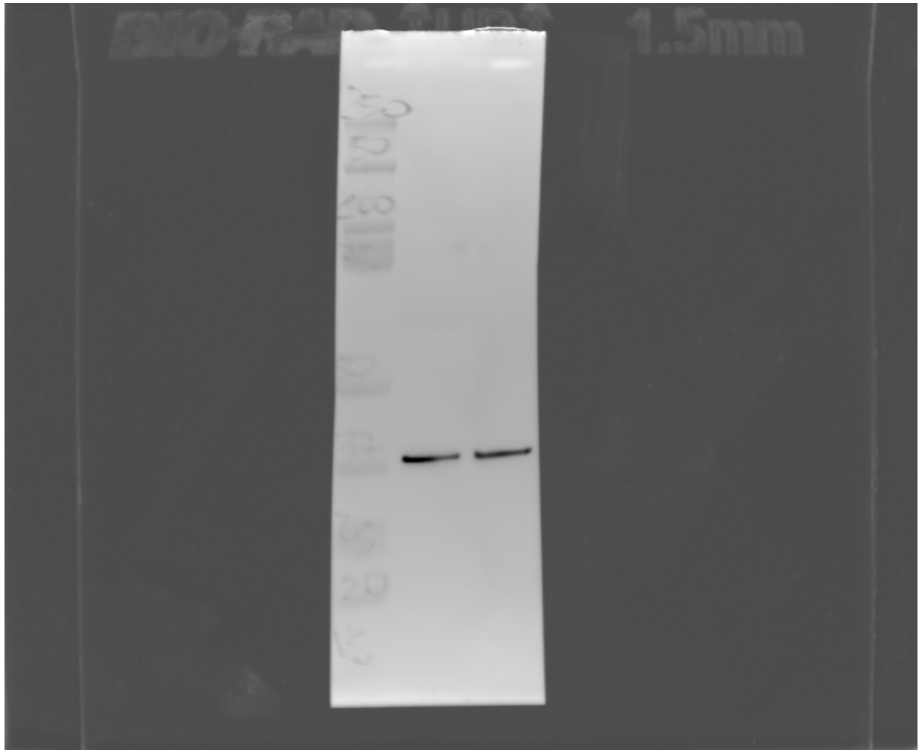

phospho-mTOR (Ser2448) and mTOR (Supplementary Figure S4A), p-Tau (Ser396) (Figure 5E) and NEFL (Figure 5C) were detected on the same membrane; GAPDH is shared among targets.

Figure 5C- NEFH

Marker: Himark pre-stained #LC5699

NEFH - 200kDa

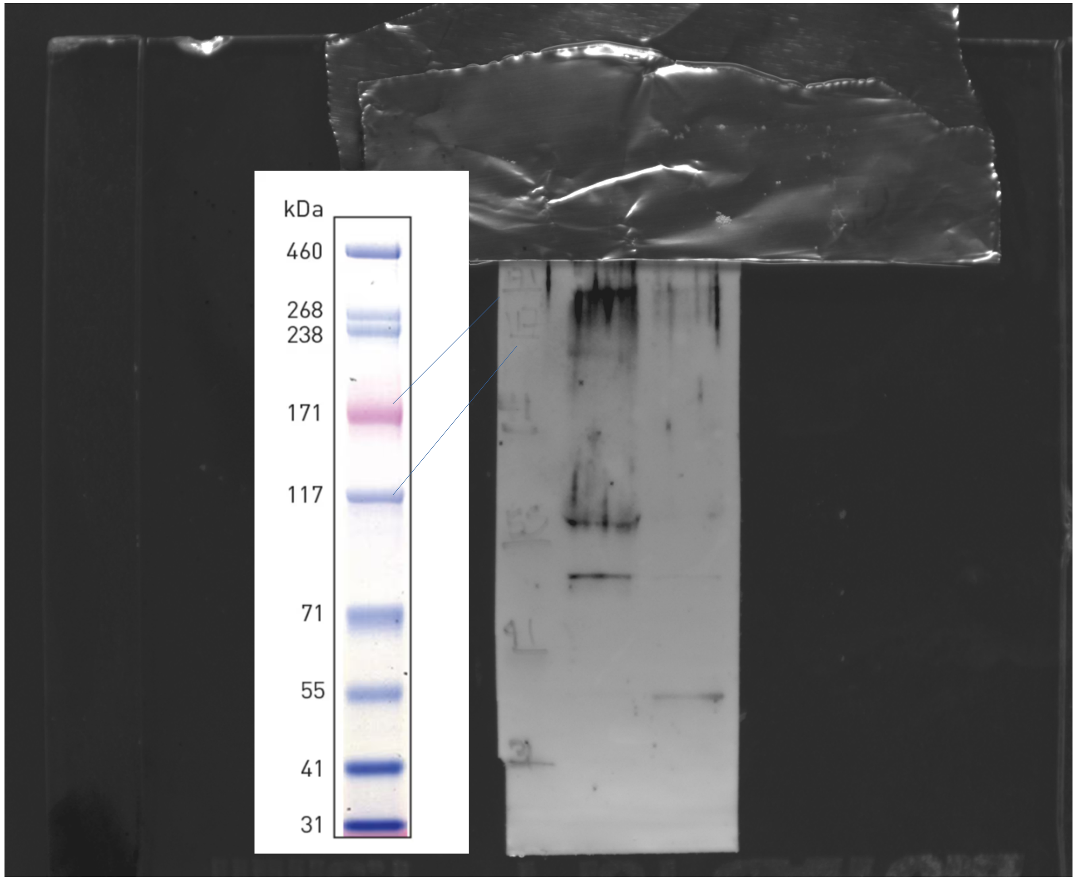

GAPDH - 37kDa

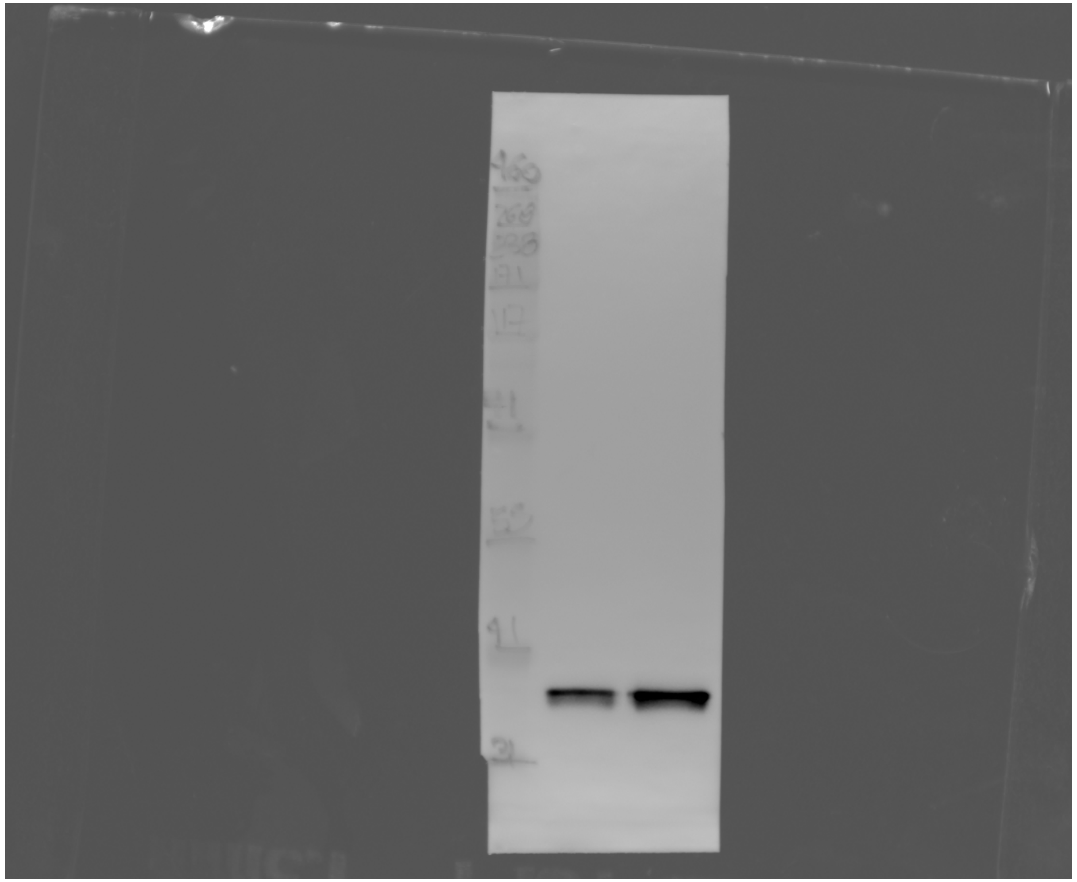

Detection of mTOR and p-mTOR for NEFH blot (Figure 5C)
Marker: Himark pre-stained #LC5699

phospho-mTOR (Ser2448)– 250kDa

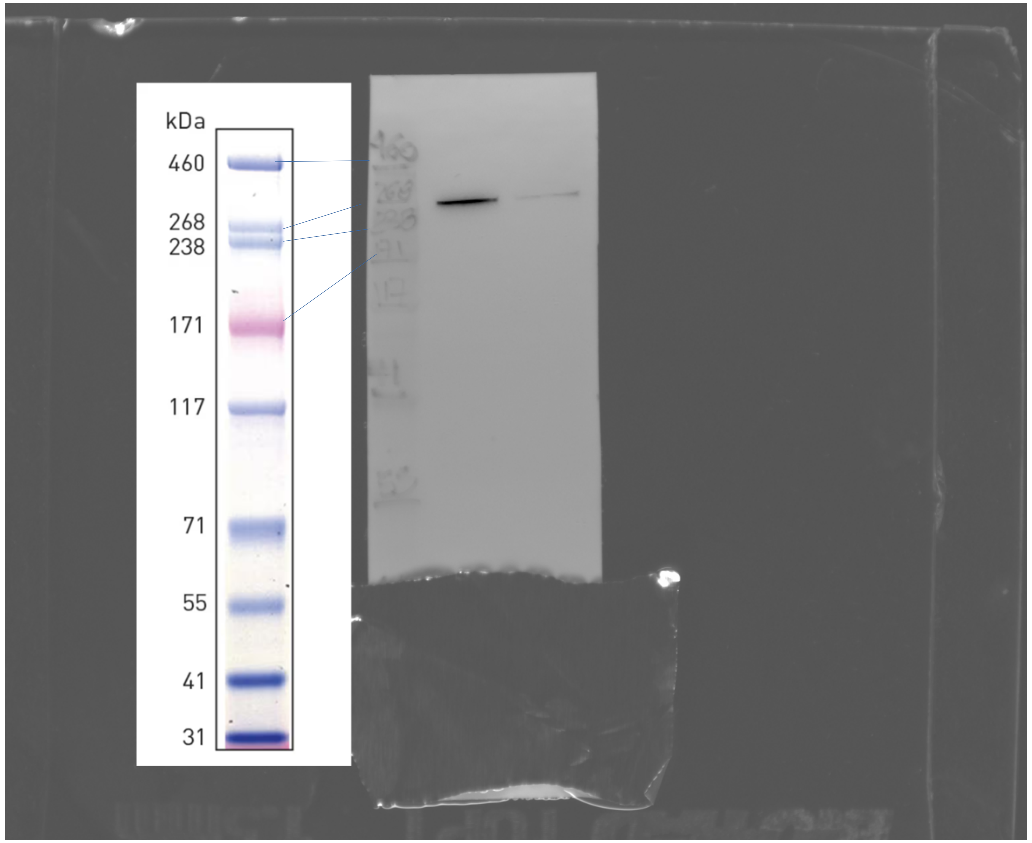

mTOR – 250kDa

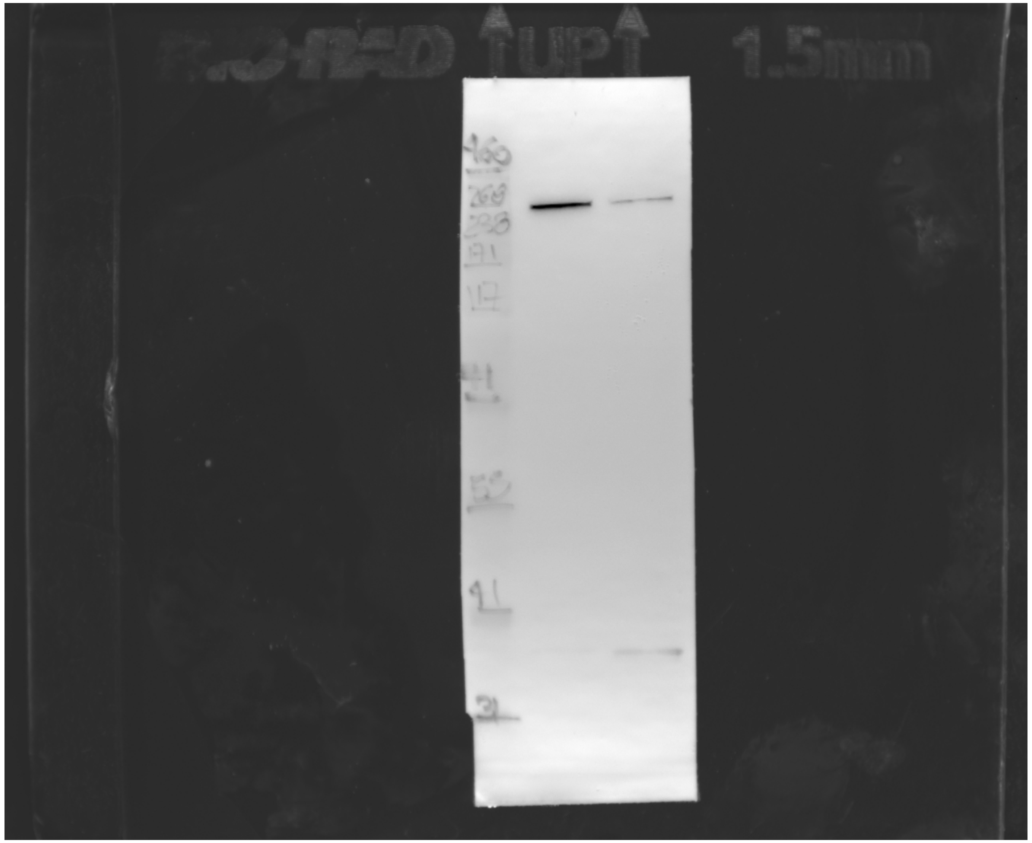

Uncropped blots of p-mTOR (Ser2448) and total mTOR in PSP-RS neurons following Torin-1 treatment (100 nM, 24 h), shown as quality control to confirm mTOR inhibition. These blots were not included in the main figures.

Figure 5E – p-Tau(ser396)

Marker: Precision Plus Protein #161-0374

p-TAU(Ser396) - 50kDa

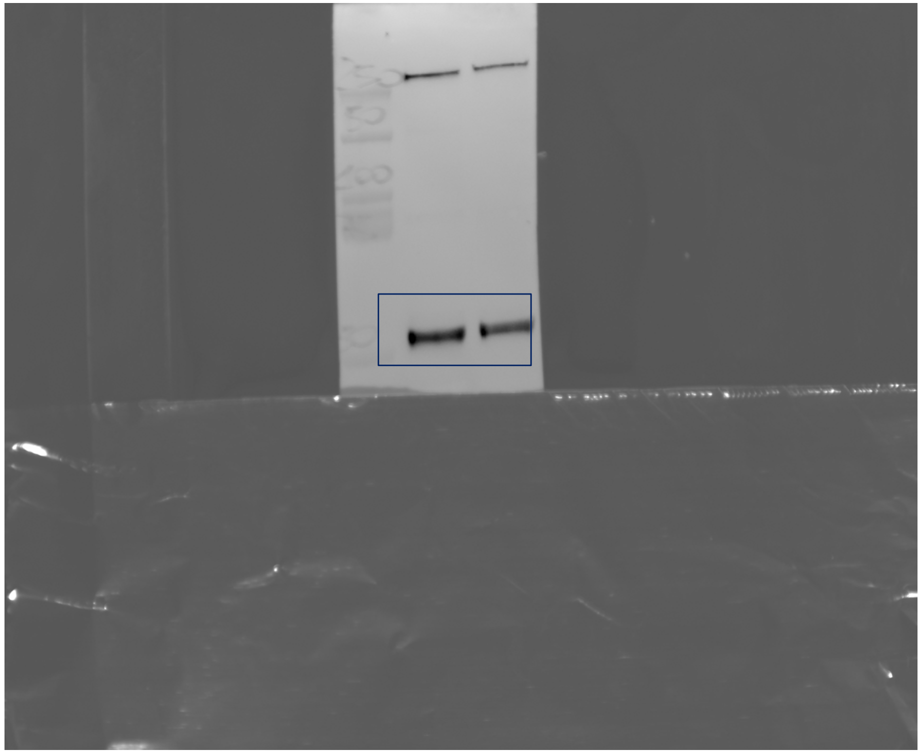

GAPDH - 37kDa

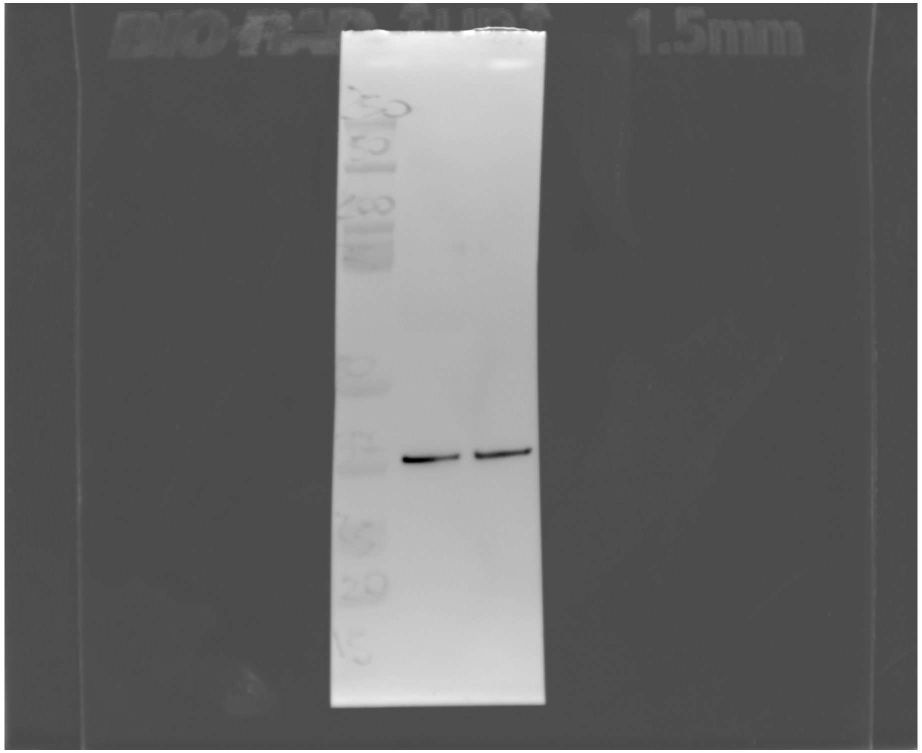

phospho-mTOR (Ser2448) and mTOR (Supplementary Figure S4A), p-Tau (Ser396) (Figure 5E) and NEFL (Figure 5C) were detected on the same membrane; GAPDH is shared among targets.

Figure 5E- p-Tau(AT8)

Marker: Precision Plus Protein #161-0374

pTau(AT8) - 50kDa

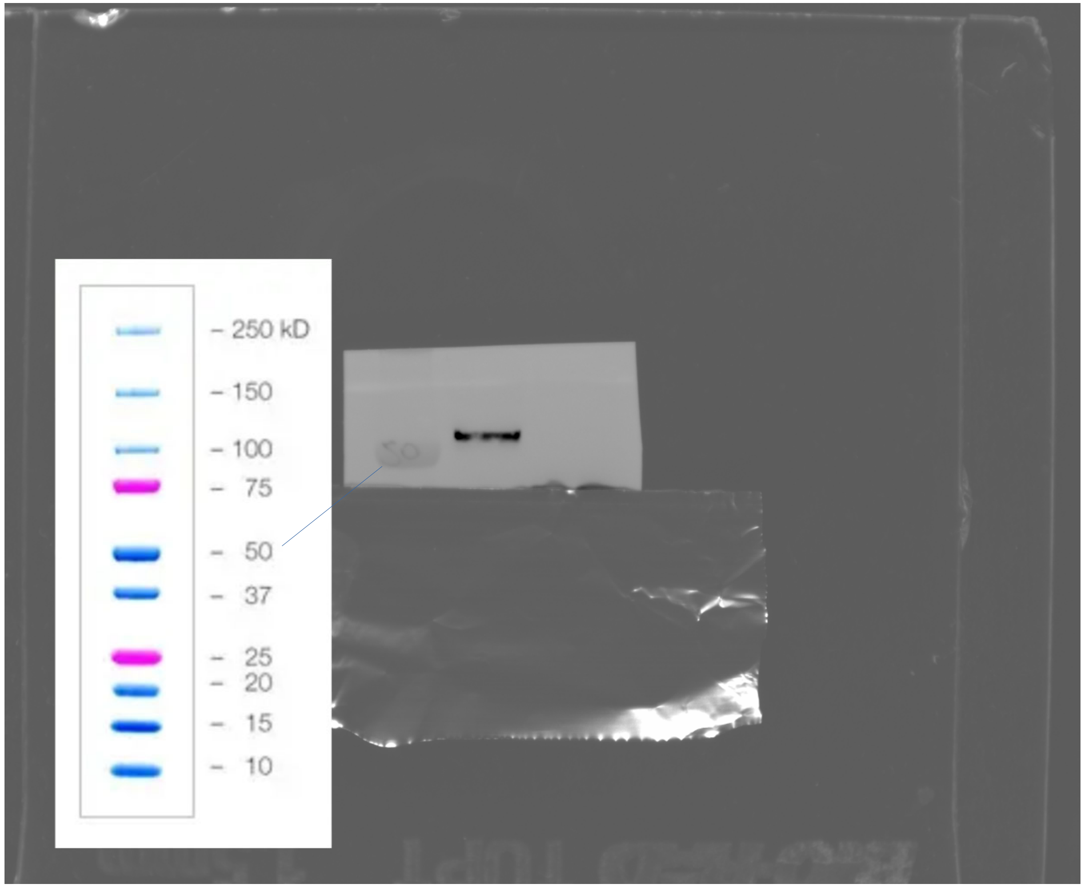

GAPDH - 37kDa

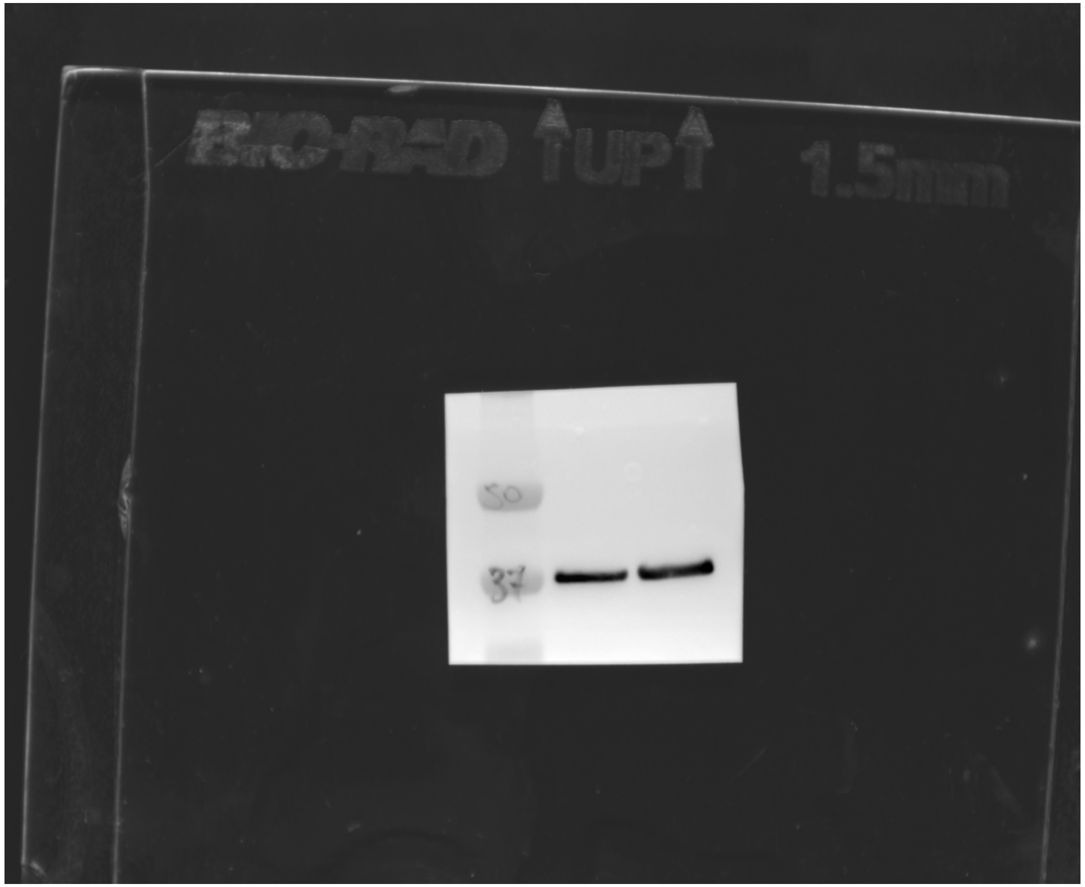

Detection of mTOR and p-mTOR for p-Tau(AT8) (Figure 5E)

Marker: Precision Plus Protein #161-0374

phospho-mTOR (Ser2448)– 250kDa

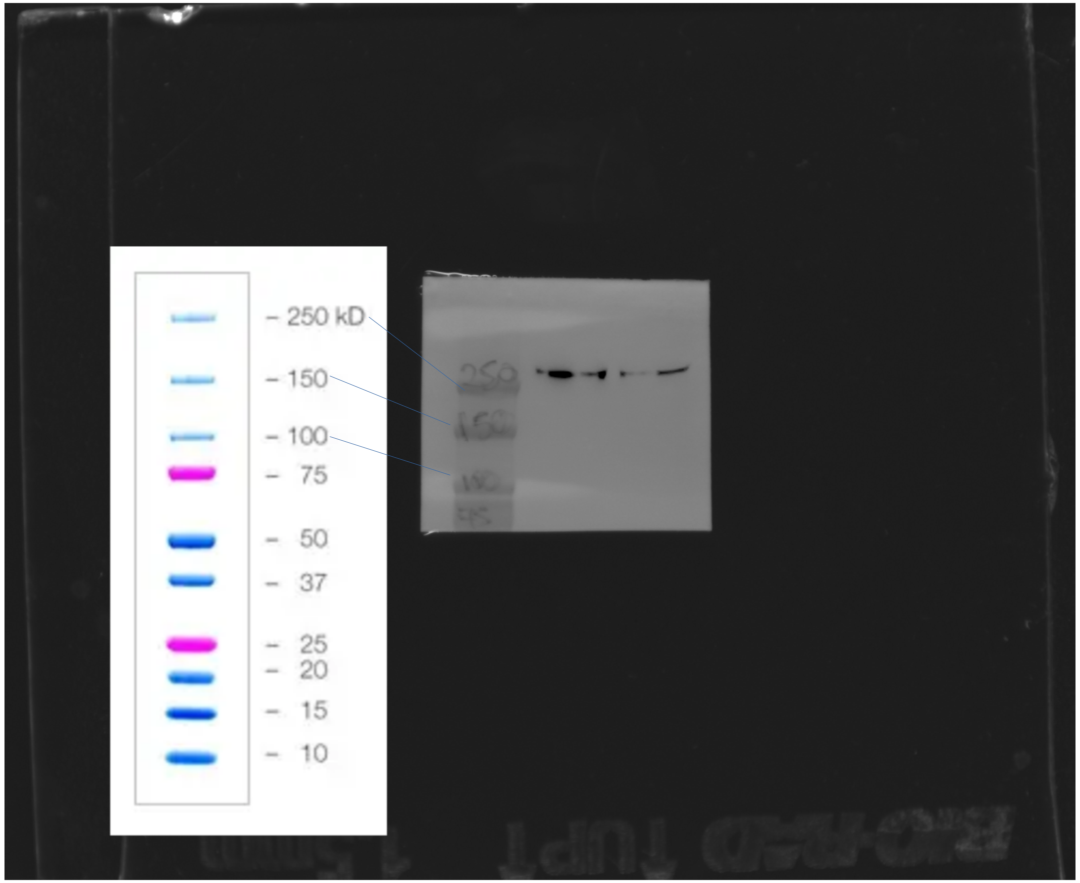

mTOR – 250kDa

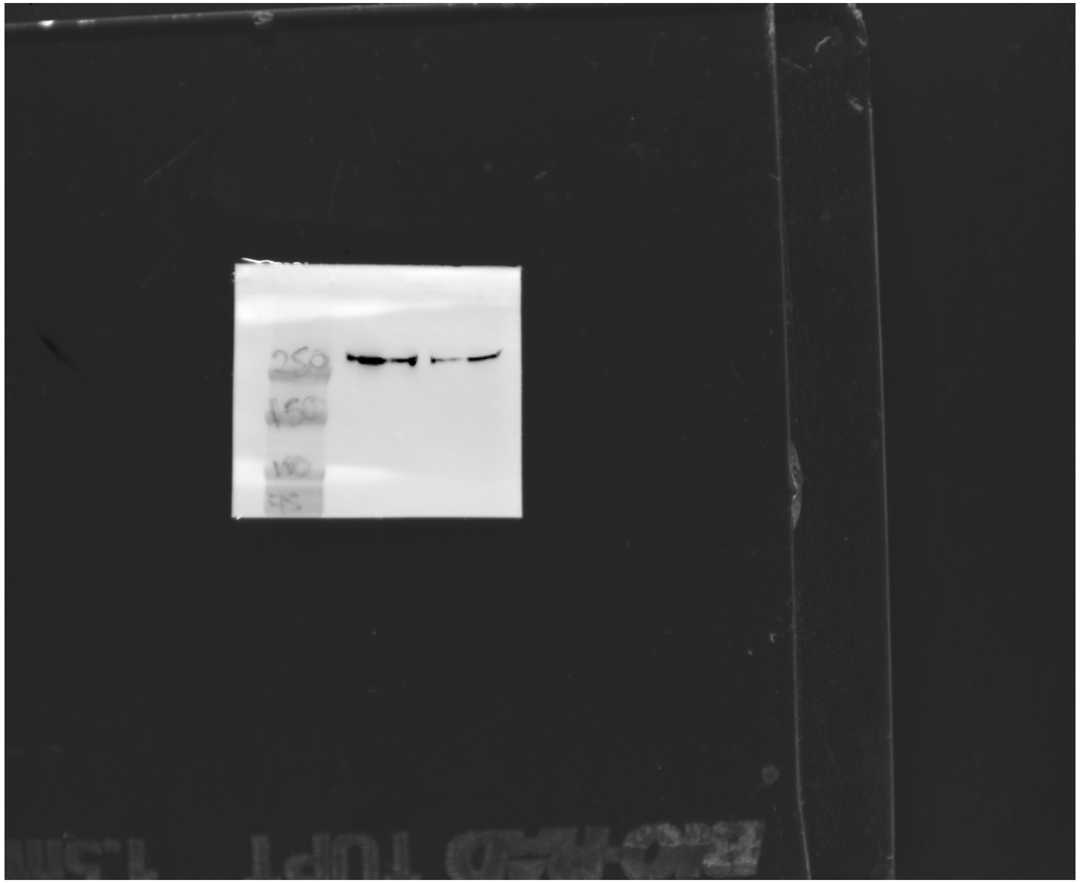

Uncropped blots of p-mTOR (Ser2448) and total mTOR in PSP-RS neurons following Torin-1 treatment (100 nM, 24 h), shown as quality control to confirm mTOR inhibition. These blots were not included in the main figures.

Figure 5E – total Tau

Marker: Precision Plus Protein #161-0374

Total Tau- 50kDa

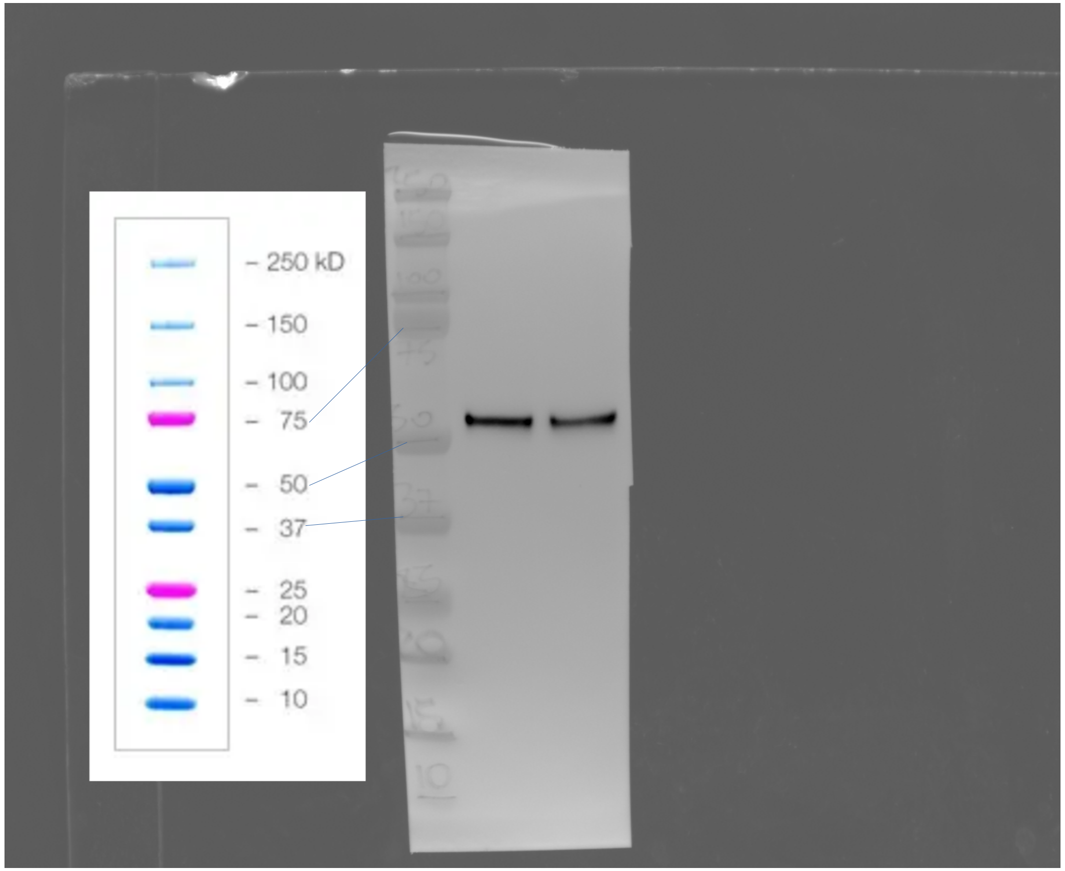

GAPDH - 37kDa

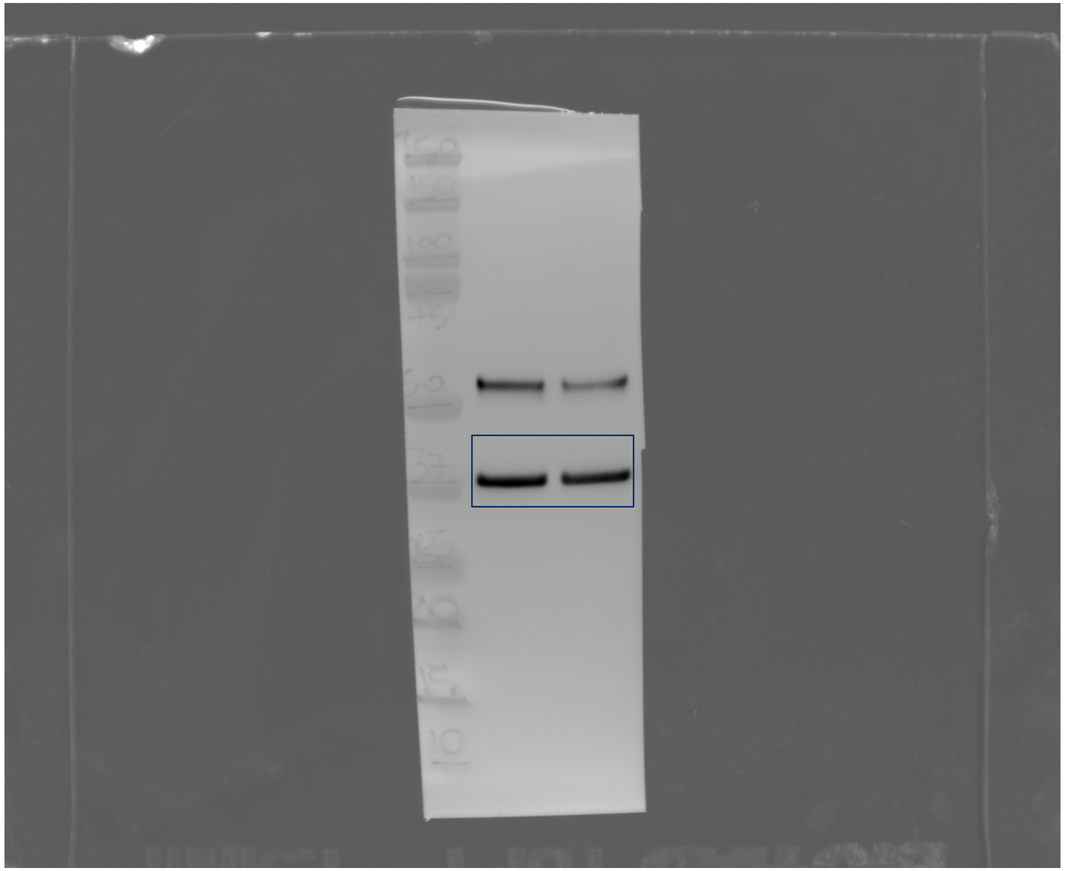

For figure 5E, Total TAU membrane used for normalization of p-Tau(Ser396) and p-Tau(AT8).

Supplementary FigureS4A- p-mTOR-mTOR

Marker: Precision Plus Protein #161-0374

phospho-mTOR (Ser2448)– 250kDa

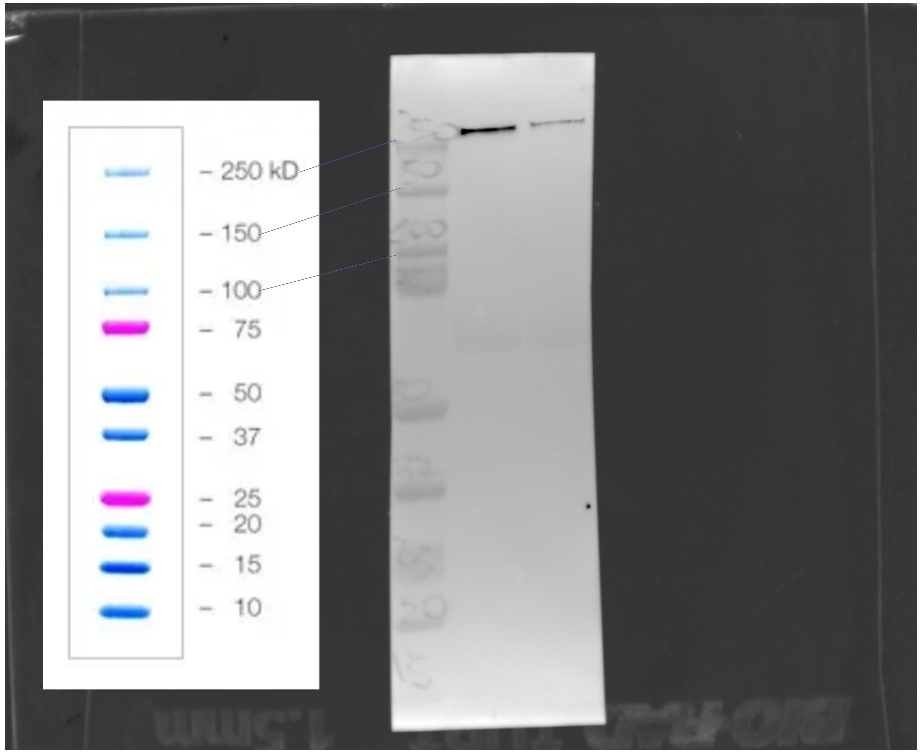

mTOR – 250kDa

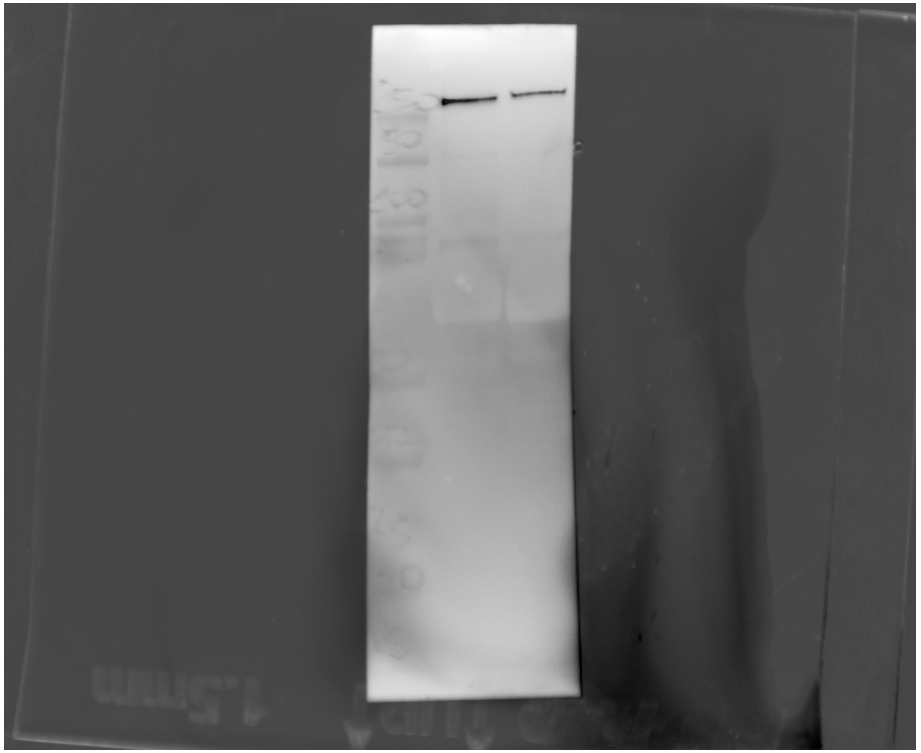

phospho-mTOR (Ser2448) and mTOR (Supplementary Figure S4A), p-Tau (Ser396) (Figure 5E) and NEFL (Figure 5C) were detected on the same membrane; GAPDH is shared among targets.

Supplementary FigureS4C- p-mTOR -mTOR

Marker: Precision Plus Protein #161-0374

phospho-mTOR (Ser2448) – 250kDa

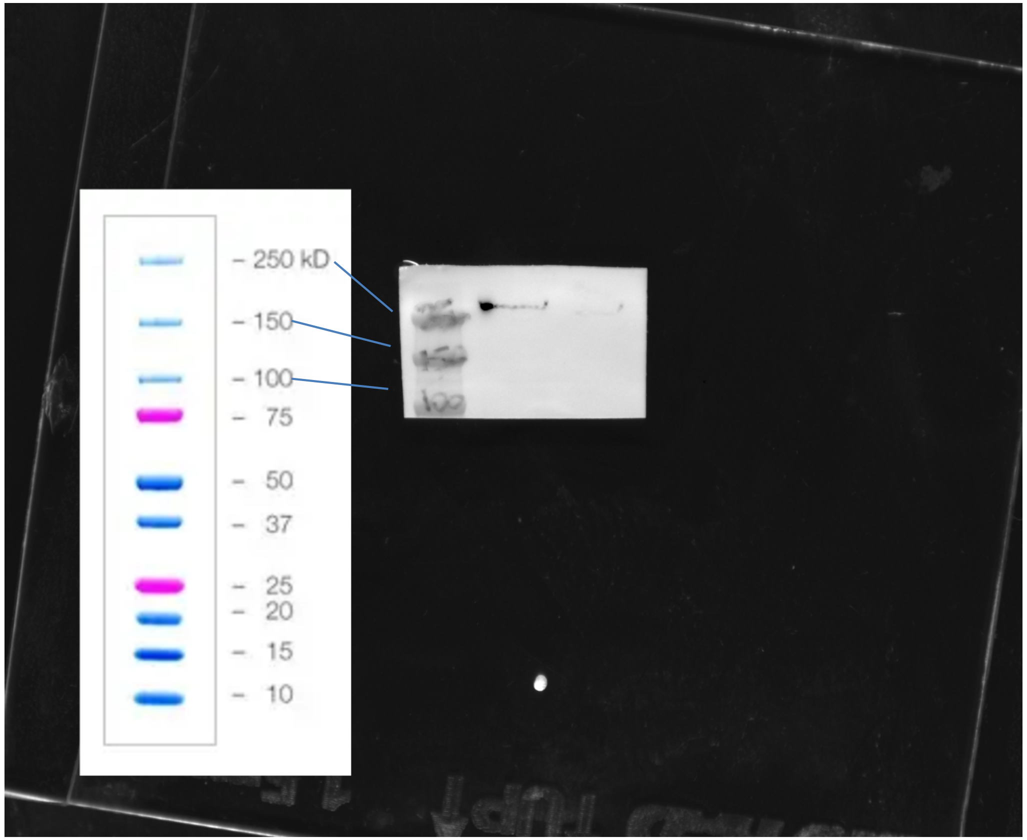

mTOR – 250kDa

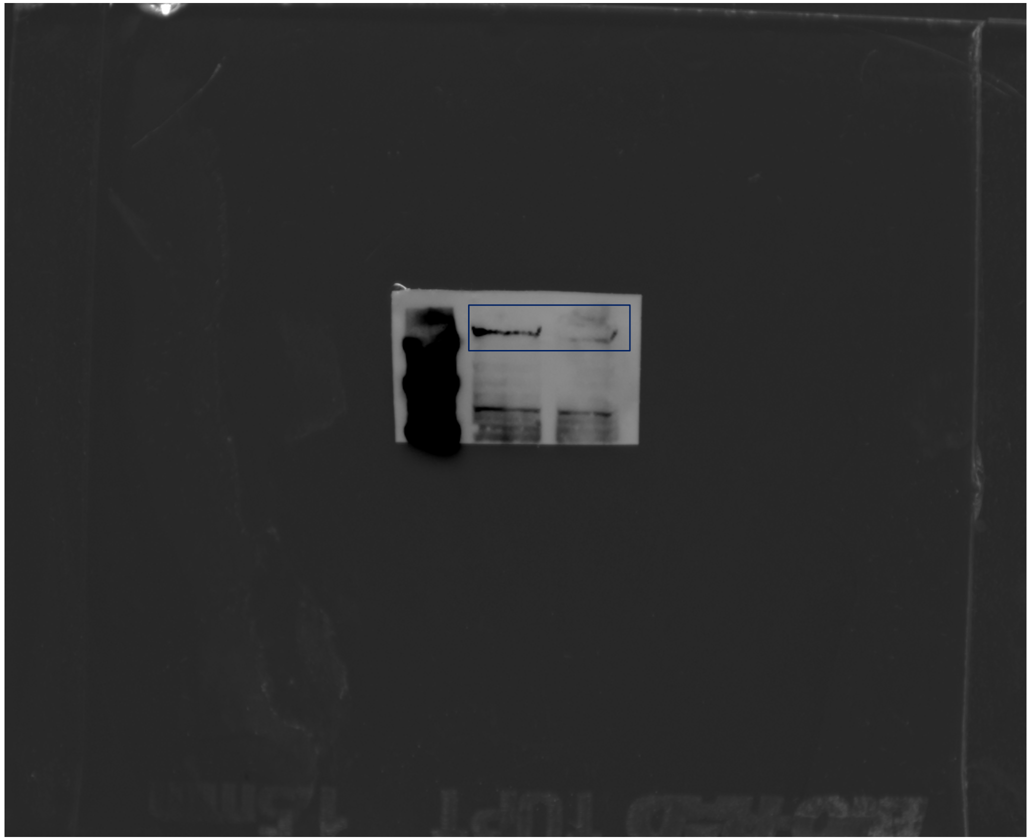

For supplementary figure S4C, phospho-mTOR (Ser2448), mTOR, p-Tau (Ser396) and NEFL were detected on the same membrane; GAPDH is shared among targets.

Supplementary Figure S4C- NEFL-p-Tau(Ser396)
Marker: Precision Plus Protein #161-0374

NEFL – 63kDa

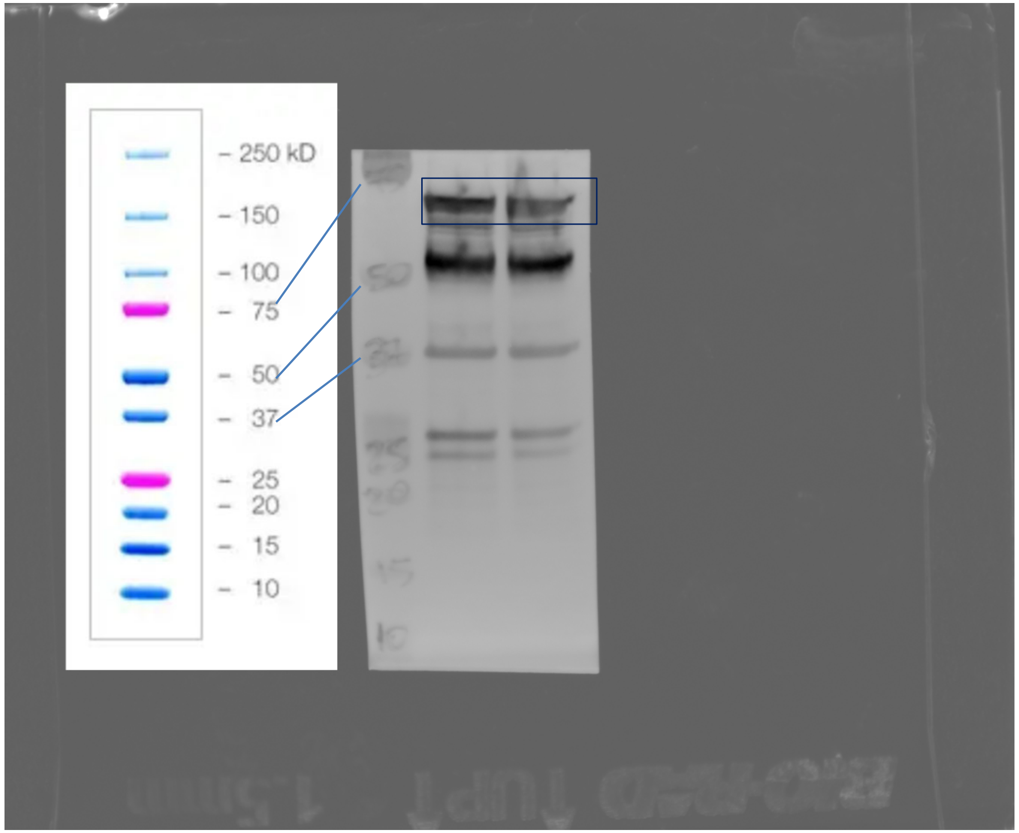

p-Tau(Ser396) - 50kDa

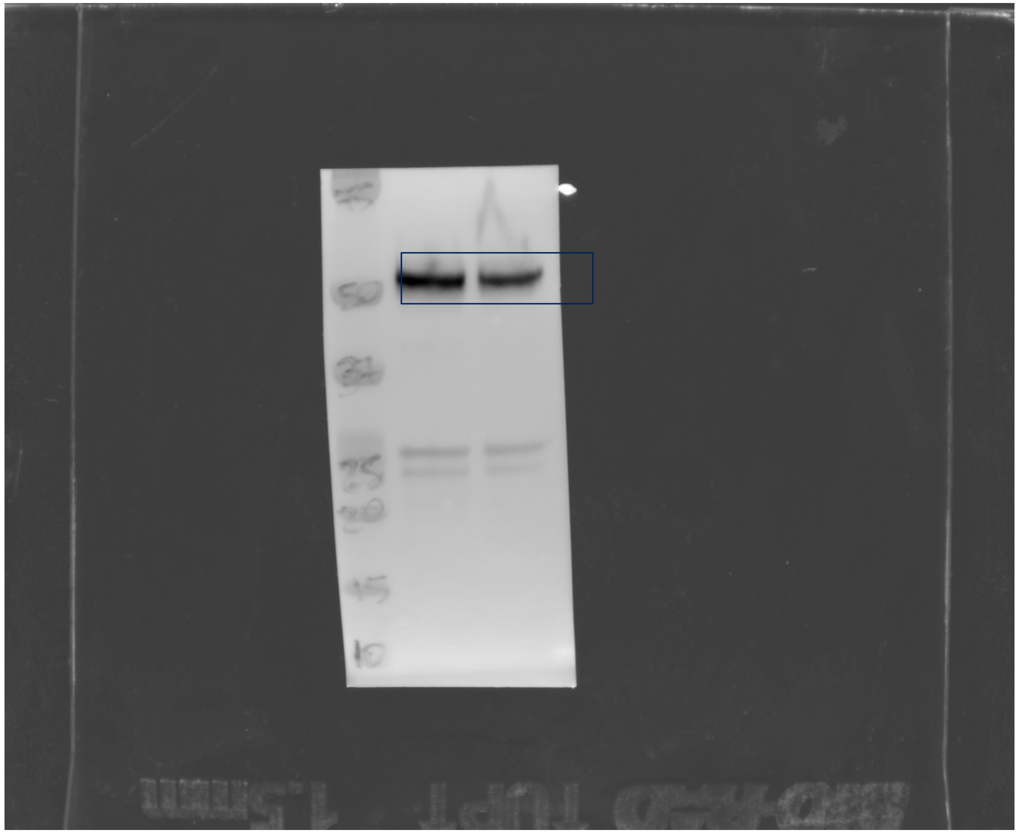

For supplementary figure S4C, phospho-mTOR (Ser2448), mTOR, p-Tau (Ser396) and NEFL were detected on the same membrane; GAPDH is shared among targets.

Supplementary Figure S4C

Marker: Precision Plus Protein #161-0374

GAPDH - 37 kDa

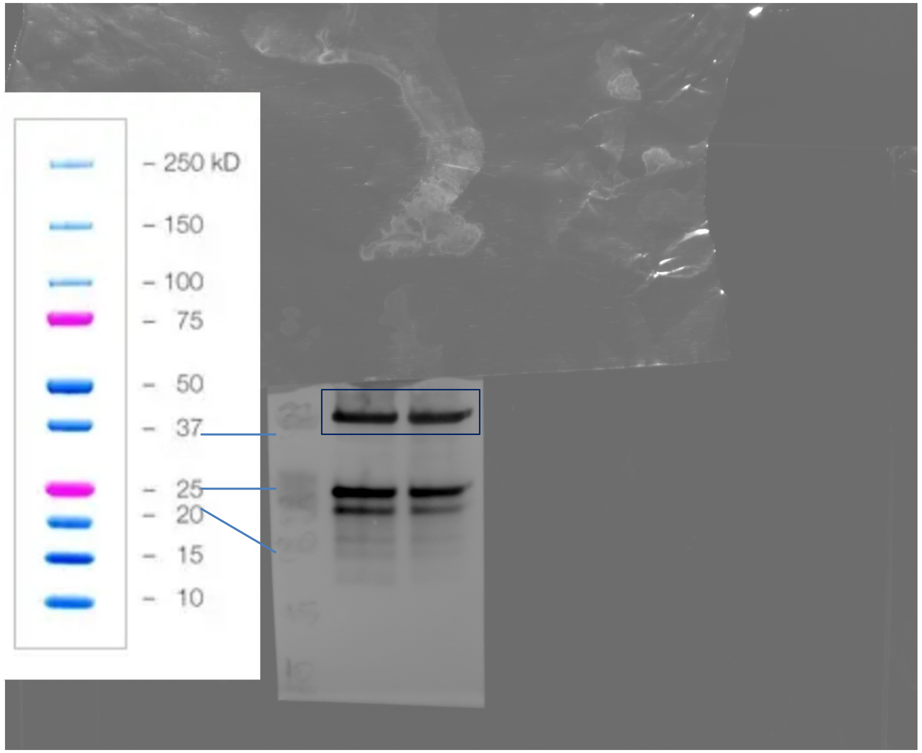

For supplementary figure S4C, phospho-mTOR (Ser2448), mTOR, p-Tau (Ser396) and NEFL were detected on the same membrane; GAPDH is shared among targets.

Supplementary Figure S4C -total Tau
Marker: Precision Plus Protein #161-0374

Total Tau- 50kDa

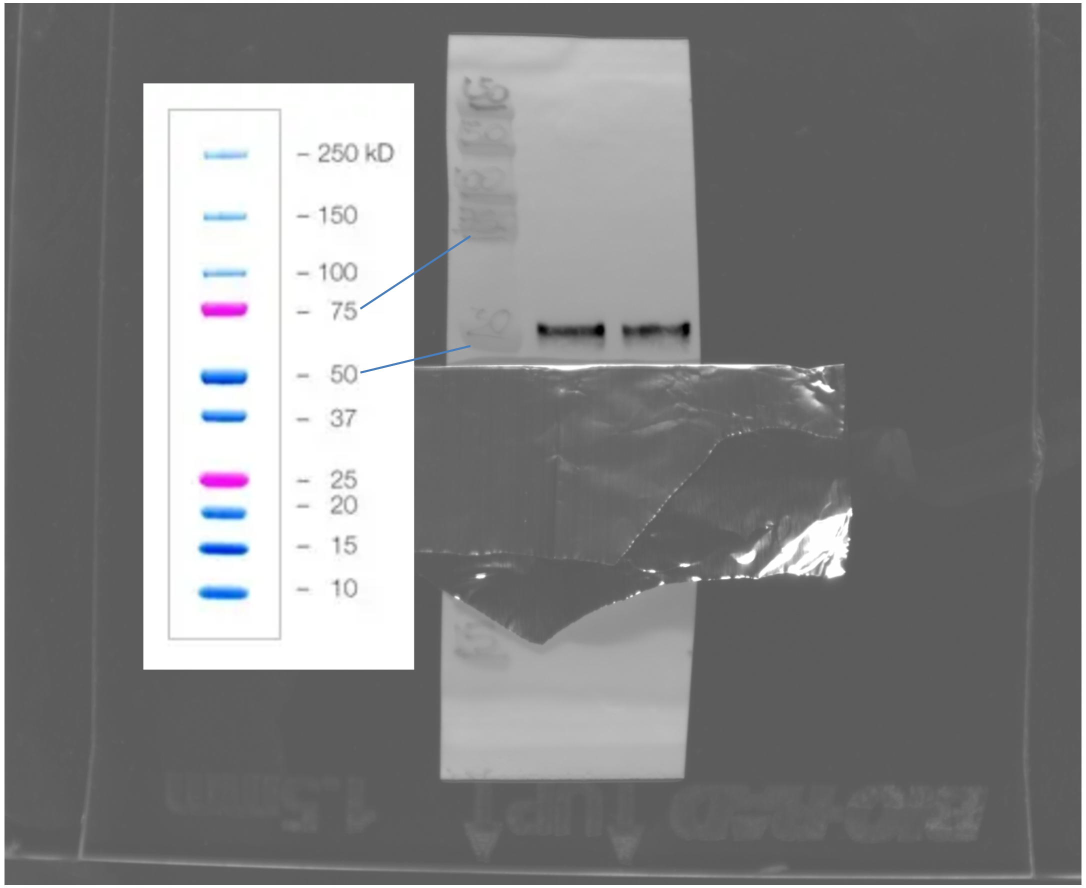

GAPDH - 37 kDa

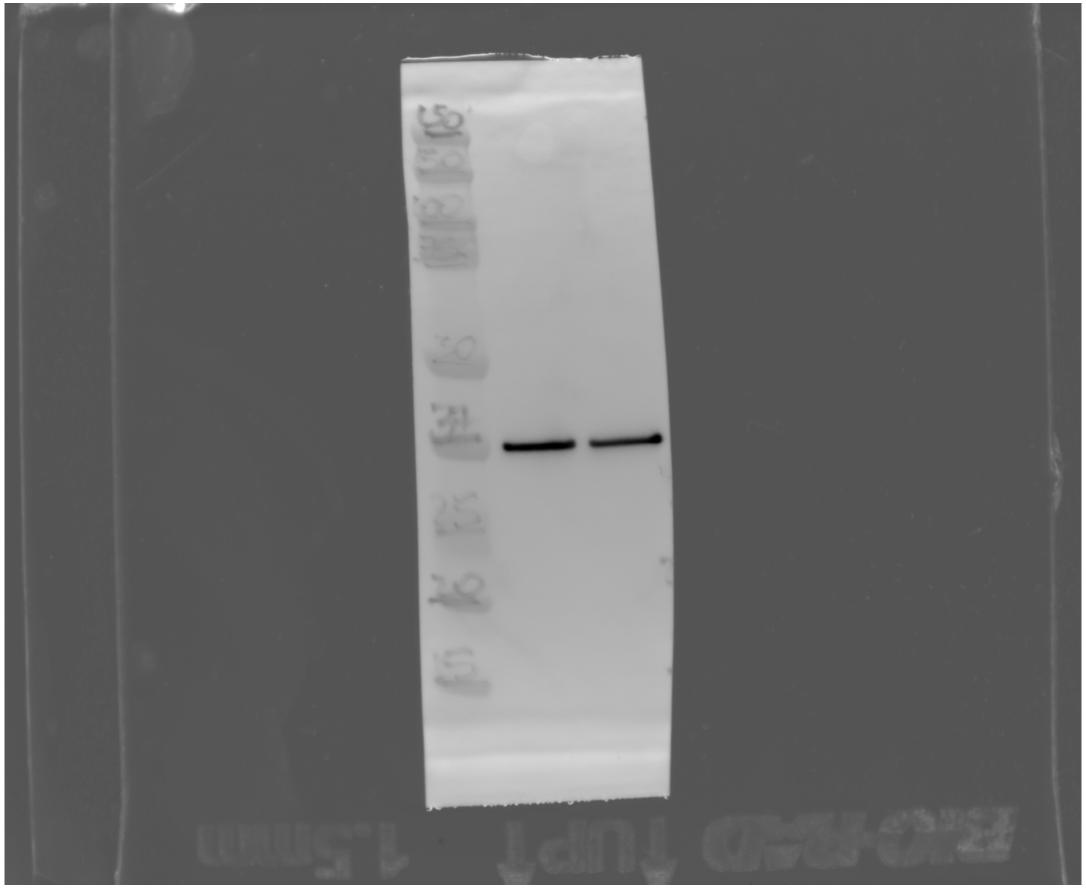

For Supplementary Figure S4C, Total Tau membrane used for normalization of p-Tau(Ser396).
