# Supplementary figures and images for: Cytoskeletal Imbalance and Axonal Vulnerability in Sporadic PSP-RS: Early Changes in a Human iPSC-Derived Neuronal Model with Altered mTOR Signaling
Source: Cells. 2026 Apr 23;15(9):754. doi: 10.3390/cells15090754 (PMC13162785; doi:10.3390/cells15090754)

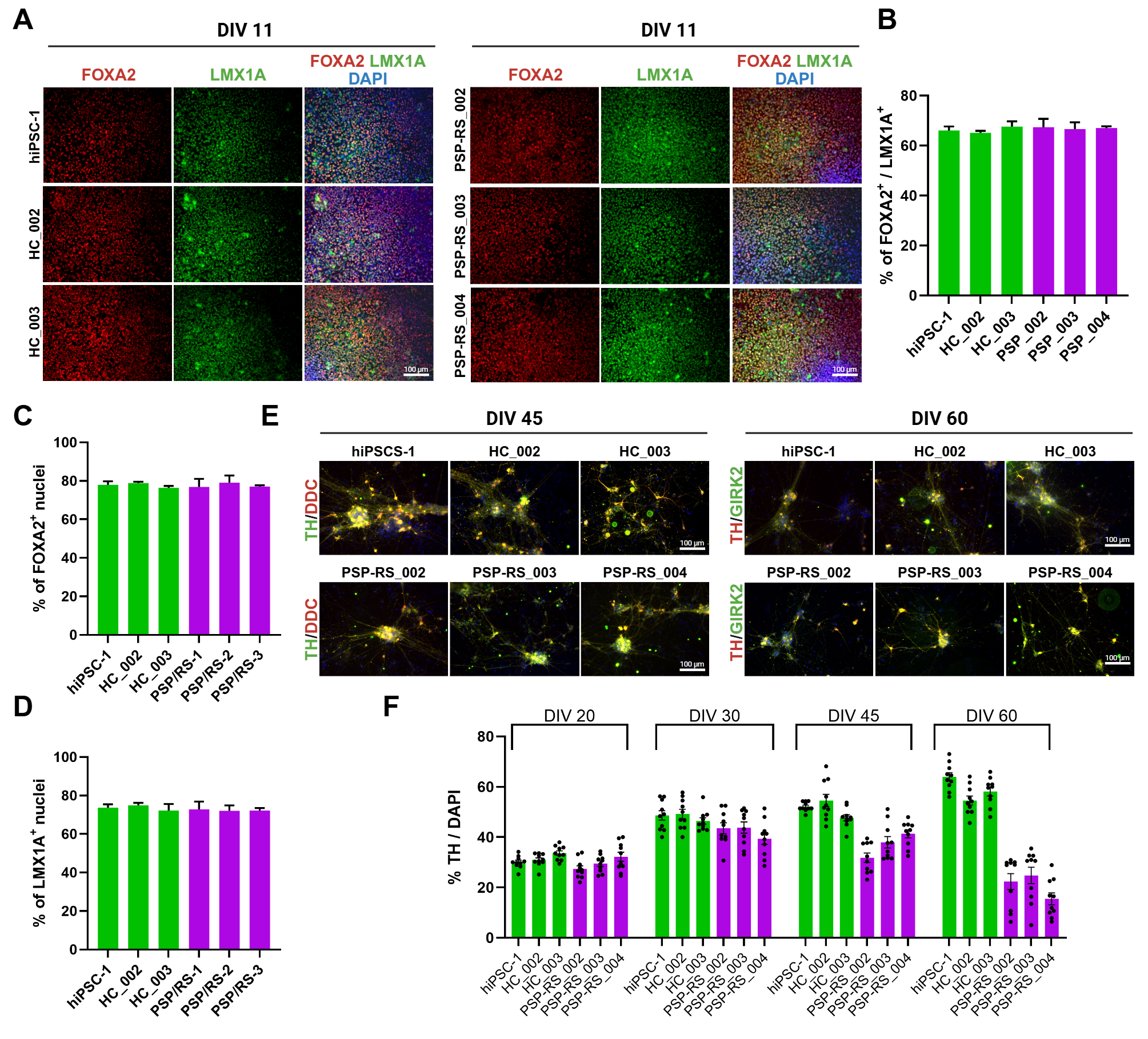

Supplement: Supplementary file 1 [file cells-15-00754-s001.zip › Supplementay Fig. S1.png]

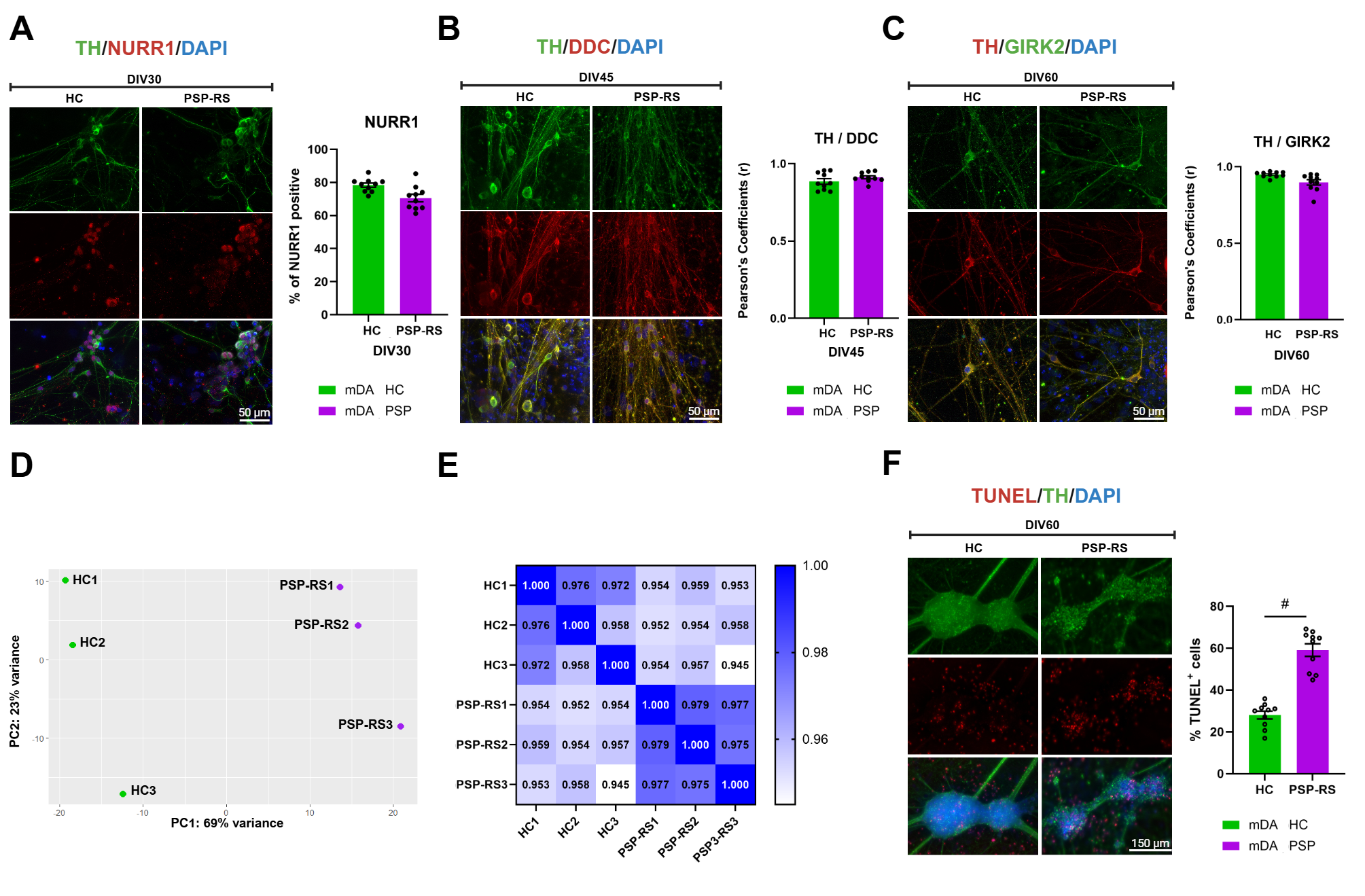

Supplement: Supplementary file 1 [file cells-15-00754-s001.zip › Supplementay Fig. S2.png]

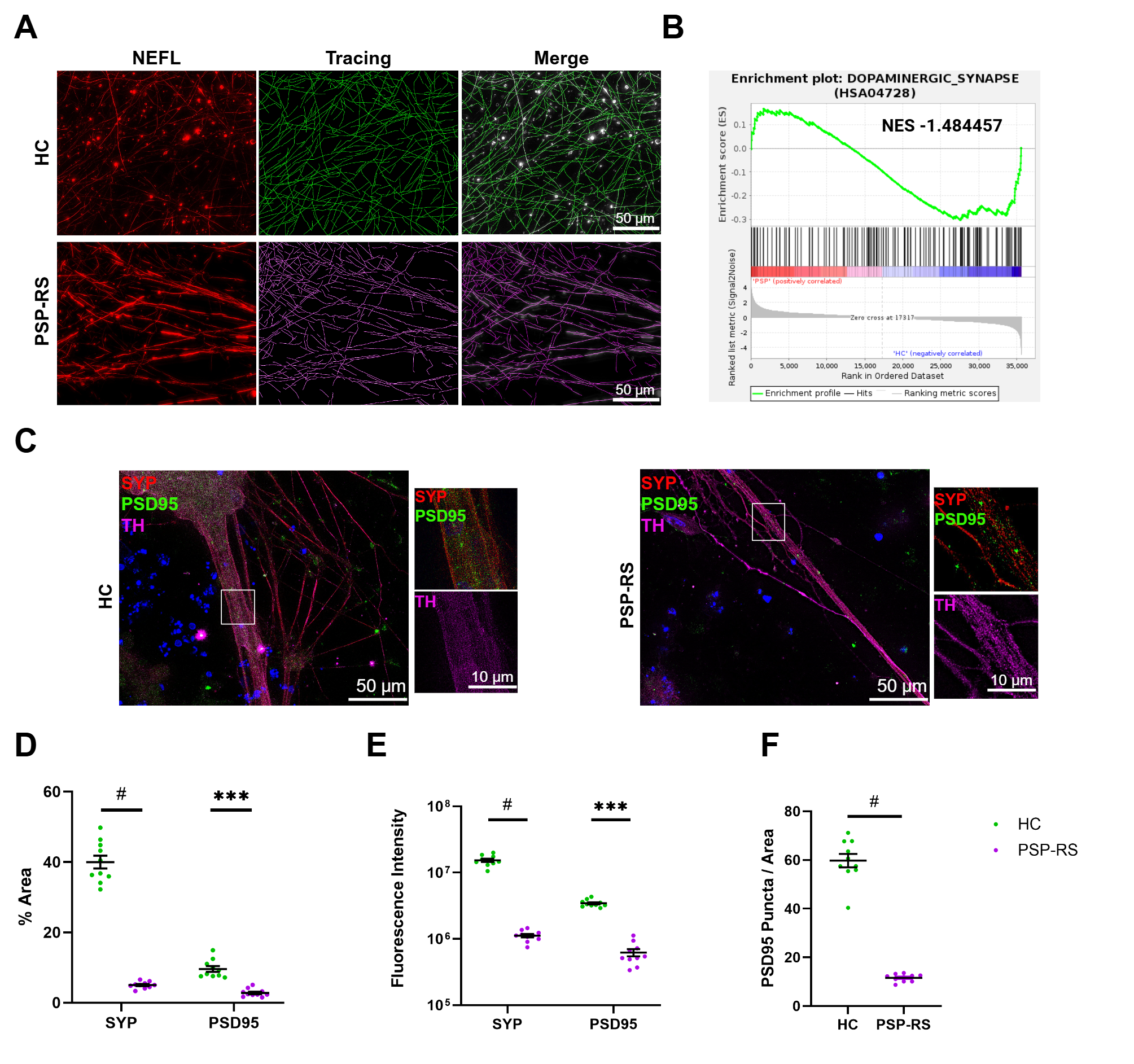

Supplement: Supplementary file 1 [file cells-15-00754-s001.zip › Supplementay Fig. S3.png]

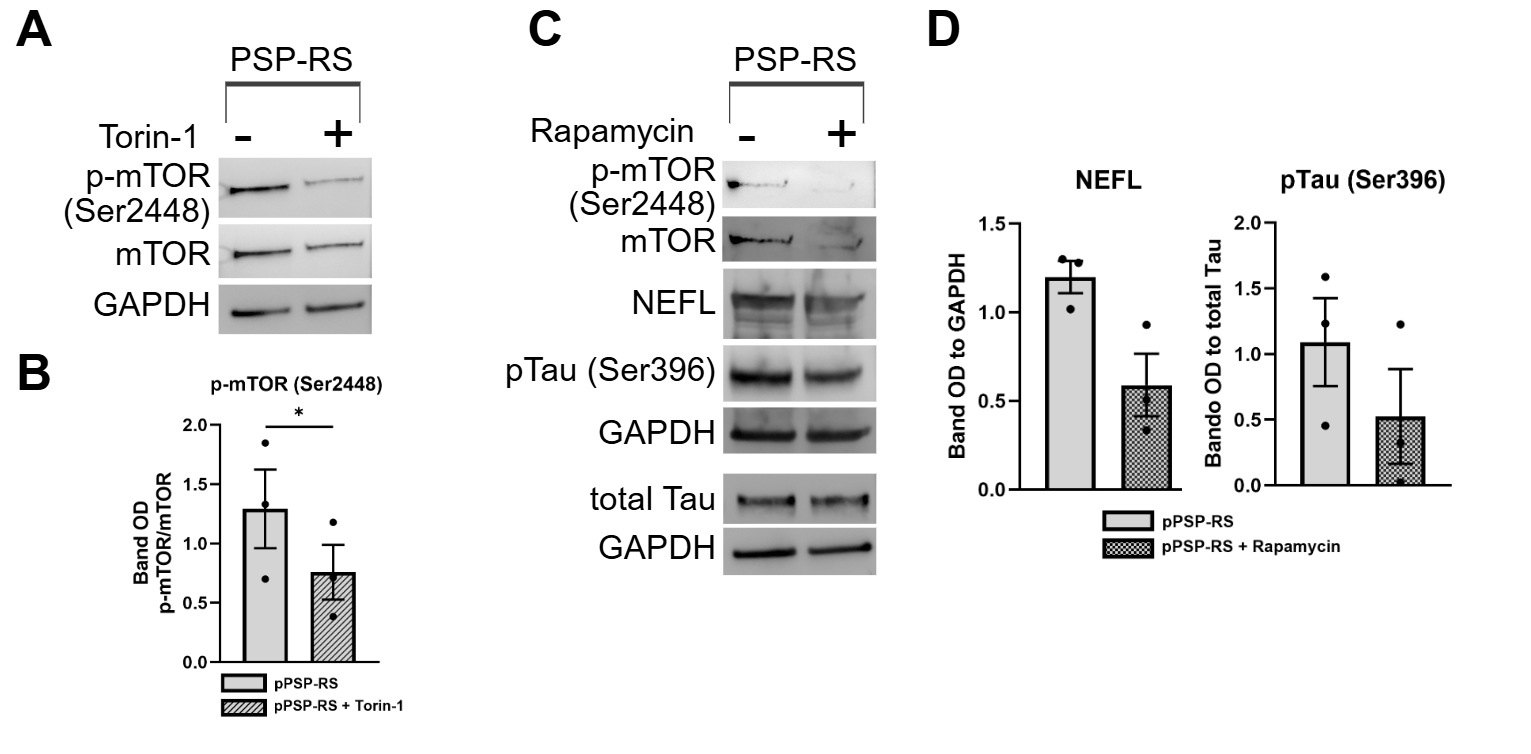

Supplement: Supplementary file 1 [file cells-15-00754-s001.zip › Supplementay Fig. S4.png]
